# Design of Benzyl-triazolopyrimidine-Based NADPH Oxidase Inhibitors Leads to the Discovery of a Potent Dual Covalent NOX2/MAOB Inhibitor

**DOI:** 10.1021/acs.jmedchem.4c02644

**Published:** 2025-03-05

**Authors:** Beatrice Noce, Sara Marchese, Marta Massari, Chiara Lambona, Joana Reis, Francesco Fiorentino, Alessia Raucci, Rossella Fioravanti, Mariana Castelôa, Alessandro Mormino, Stefano Garofalo, Cristina Limatola, Lorenzo Basile, Andrea Gottinger, Claudia Binda, Andrea Mattevi, Antonello Mai, Sergio Valente

**Affiliations:** a Department of Drug Chemistry and Technologies, Sapienza University of Rome, P.le Aldo Moro 5, Rome 00185, Italy; b Department of Biology and Biotechnology Lazzaro Spallanzani, University of Pavia, Via Adolfo Ferrata 9A, Pavia 27100, Italy; c CIQUP-IMS/Department of Chemistry and Biochemistry, Faculty of Sciences, University of Porto, Rua do Campo Alegre s/n, Porto 4169-007, Portugal; d Department of Physiology and Pharmacology, Sapienza University of Rome, P.le Aldo Moro 5, Rome 00185, Italy

## Abstract

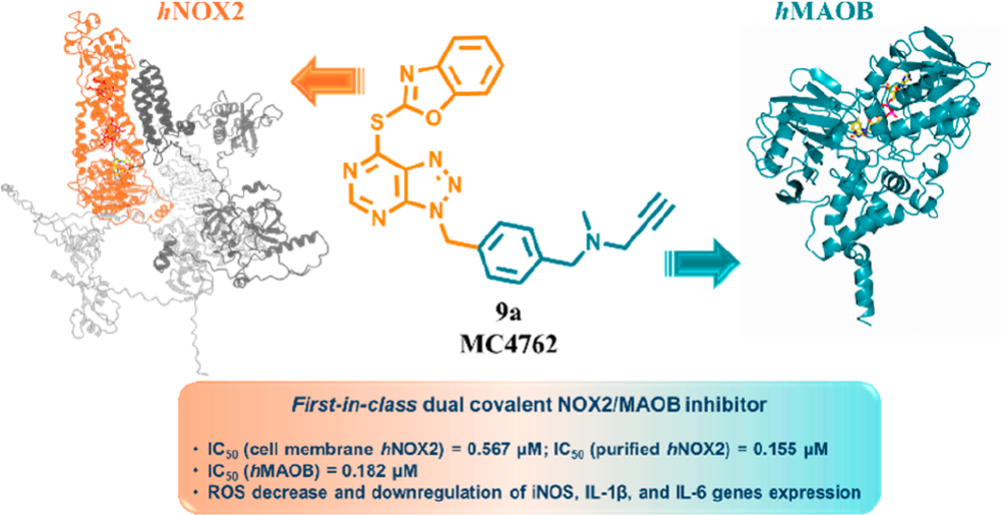

NADPH oxidases (NOXs) are enzymes dedicated to reactive oxygen species (ROS) production and are implicated in cancer, neuroinflammation, and neurodegenerative diseases. VAS2870 is a covalent inhibitor of mainly NOX2 and NOX5. It alkylates a conserved active-site cysteine, blocking productive substrate binding. To enhance potency and selectivity toward NOXs, we conducted some chemical modifications, leading to the discovery of compound **9a** that preferentially inhibits NOX2 with an IC_50_ of 0.155 μM, and only upon its preactivation. We found that **9a**, bearing a pargyline moiety, is also able to selectively inhibit MAOB over MAOA (465-fold) with an IC_50_ of 0.182 μM, being the first-in-class dual NOX2/MAOB covalent inhibitor. Tested in the BV2 microglia neuroinflammation model, **9a** decreased ROS production and downregulated proinflammatory cytokines as iNOS, IL-1β, and IL-6 expression more efficiently than the single target inhibitors (rasagiline for MAOB and VAS2870 for NOXs) but also, more importantly, than their combination.

## Introduction

NADPH oxidases (NOXs) generate extracellular reactive oxygen species (ROS) by transferring electrons across biological membranes, ultimately reducing molecular oxygen.^1−7^ NOX2, the microbial killer in phagocytic cells, was the first isoform to be discovered.^8,9^ Six other isoforms were then identified, for a total of seven distinct mammalian homologues (NOX1–5 and dual oxidases 1–2).^4^ These enzymes share a very similar catalytic subunit that comprises the binding sites for the flavin adenine dinucleotide (FAD) and nicotinamide adenine dinucleotide phosphate (NADPH) in the dehydrogenase domain and two noncovalent heme groups coordinated by two histidine pairs in the transmembrane domain.^10^ Besides these common features, each isoform presents specific items, differing for the tissue distribution, activation mechanism, and functions. NOX2 consists of a multiprotein complex comprising a flavocytochrome b_558_ (an assembly of the NOX2 and the p22^phox^ proteins), three cytosolic activators (p47^phox^, p67^phox^, and p40^phox^), and a small GTPase (Rac1).^11^ A similar subunit composition and cytosolic activating complexes characterize also NOX1 and NOX3.^12^ NOX5, DUOX1, and DUOX2 are instead regulated in a calcium-dependent manner, whereas NOX4 is constitutively active, though less efficient when compared to the other family members.^13−19^

NOXs have been implicated in a wide range of pathological conditions and are appealing pharmacological targets for immunomodulation and cancer as well as in the context of neuroinflammation. Notably, the nervous system is particularly sensitive to oxidative stress and understanding the mechanisms of ROS production and their impact is crucial for developing potential treatments or interventions for conditions like Parkinson’s disease, Alzheimer’s disease, and amyotrophic lateral sclerosis.^20,21^ For this reason, the function of NOXs as a source of ROS in the central nervous system has been extensively studied. NOX2 is the main isoform in the brain, and its activation is associated with neurodegeneration marks such as reduction in the number of brain capillaries, loss of neurons, and locomotor disorders.^22,23^ Overexpression of NOX2 leads to α-synuclein and amyloid β (Aβ) aggregates, distinctive hallmarks of Parkinson’s and Alzheimer’s diseases, respectively.^24,25^ In addition to NOXs, monoamine oxidases A and B (MAOA and MAOB) are also recognized as the main sources of ROS in neuroinflammation. MAOA and MAOB are mitochondrial outer-membrane enzymes that break the Cα–N bond of arylalkylamines, including neurotransmitters like dopamine, through a FAD-dependent oxidative deamination, and both enzymes play a role in age-related neurological disorders.^26^ In particular, MAOB is a validated drug target for Parkinson’s disease^27^ and multiple lines of evidence suggest that MAOB is involved also in Alzheimer’s disease.^28^ Therefore, targeting both NOXs and MAOs may be a promising pharmacological strategy to mitigate ROS-mediated neuroinflammation and neuronal damage.

**Figure 1:**
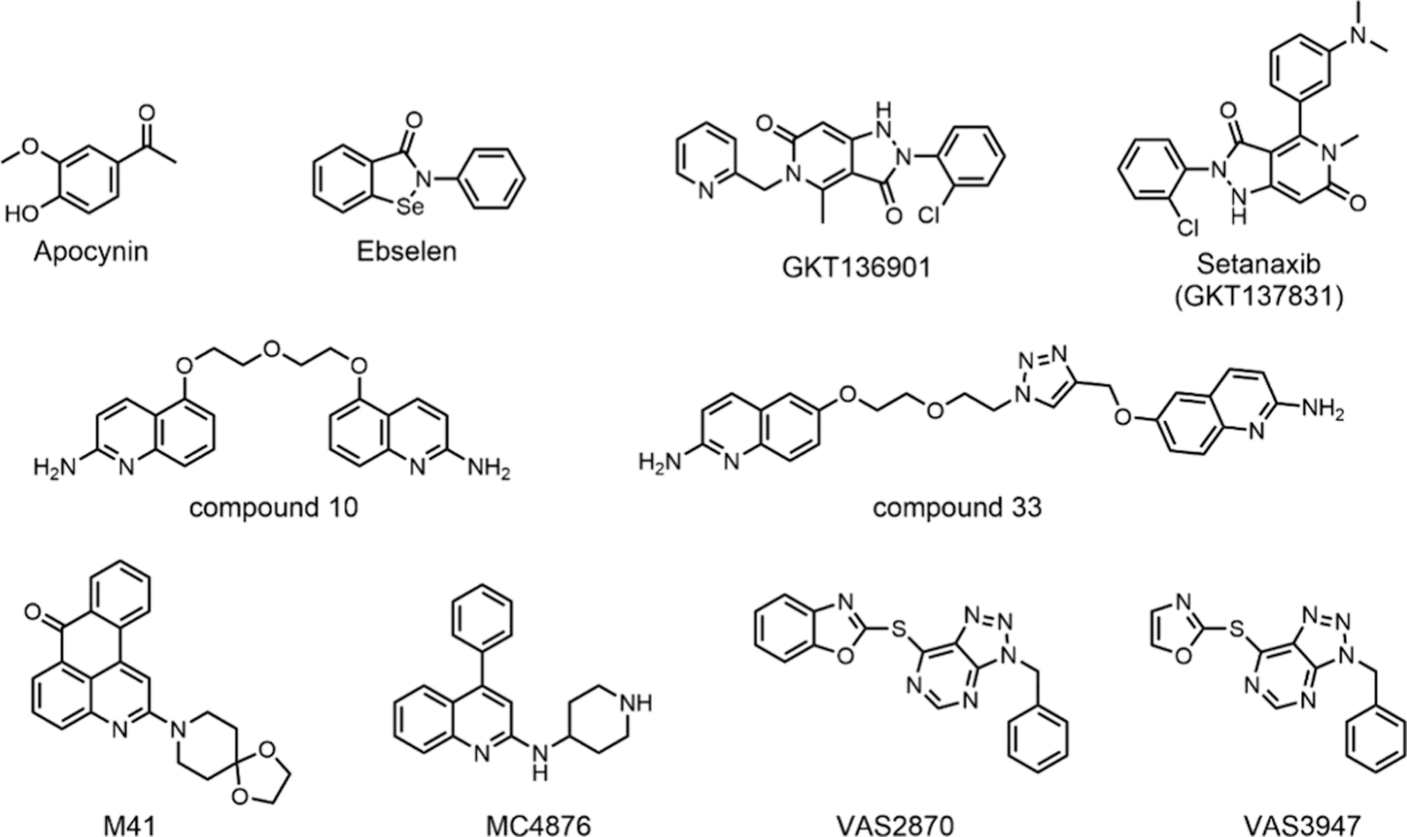
Chemical structures of reported NOX inhibitors.^29−40^

The World Health Organization has introduced the acronym “naxib” to define the class of NADPH oxidase inhibitors,^41^ and Setanaxib (known before as GKT137831) has been the first naxib to reach clinical trials. In general, NOX inhibitors known until now are not selective for the different homologues and, most critically, suffer from both assay-interfering and off-target activities (Figure 1).^29−38^ Hence, it has been difficult to discern between the nonspecific effects exerted by these compounds and their specific binding to NOXs (if any).^42,43^ Only recently, potent indirect inhibitors of NOX2 were identified that directly bind to p47^phox^ and hamper its interaction with NOX2 (compounds **10** and **33**, Figure 1).^39,44^ Furthermore, by establishing an extensive platform of biochemical and biophysical assays, we have recently reported the first direct NOX inhibitors, identified from an ultralarge *in silico* screening of a total of 350 million compounds and validated by structural, *in vitro*, and *in cellulo* assays.^40^ The most powerful compound is especially active against human NOX2 and NOX4 isoforms (IC_50_ NOX2 = 5.1 μM, IC_50_ NOX4 = 5.7 μM; M41 in Figure 1, compound **3** in ref (40)). We next designed MC4876 (compound **15** in ref (40)) that preferentially inhibits NOX2 (IC_50_ NOX2 = 7.7 μM) while retaining activities against other NOXs (IC_50_ NOX1 = 71.2 μM, IC_50_ NOX4 = 81.2 μM, and IC_50_ NOX5 = 28 μM). In cells, both M41 and MC4876 displayed stronger inhibition of NOX2 (EC_50_ = 2.1 μM and EC_50_ = 5.7 μM) over the other isoforms.^40^ Moreover, both M41 and MC4876 reduced the viability of monocytic U937 and other myeloid cancer lines, in agreement with the role of NOX2 in these cells.^40^

**Figure 2:**
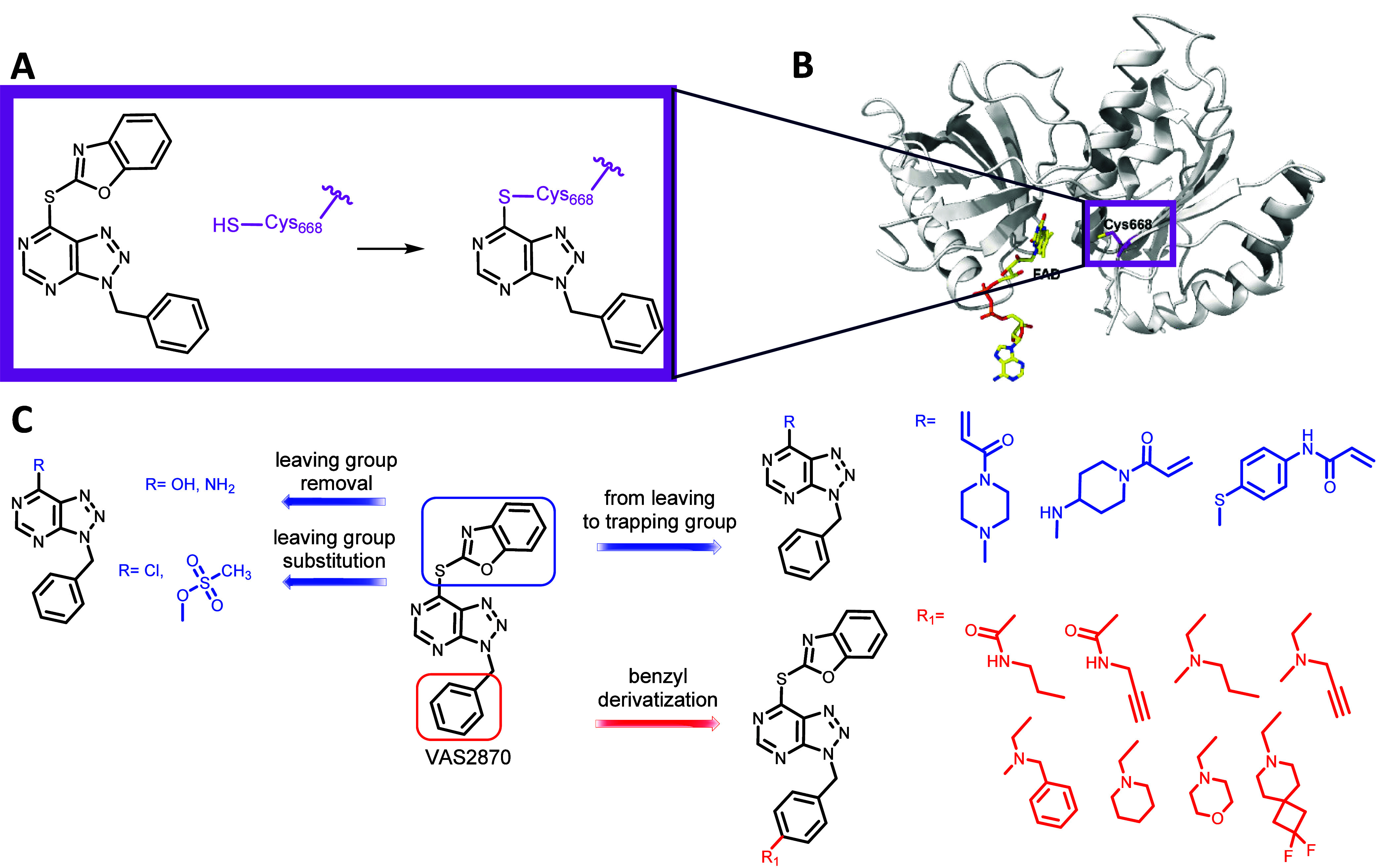
Outline of this work. (A) Structure of the VAS2870-cysteine adduct. (B) Three-dimensional structure of the *Cylindrospermum stagnale* NOX5 dehydrogenase domain highlighting the targeted Cys668 and its proximity to the flavin. (C) Design of triazolopyrimidine-containing NOX inhibitors.

Differently from the above-discussed molecules, VAS2870 and VAS3947 operate by forming a covalent adduct with an active-site cysteine, located in the proximity of the FAD’s isoalloxazine ring and conserved in all NOXs (Figure 1).^45,46^ VAS2870 is more potent than its oxazole analogue, VAS3947 (VAS2870: IC_50_ NOX1 = 72.6 μM, NOX2 = 1.1 μM, NOX4 = 12.3 μM, and NOX5 = 1.8 μM; VAS3947: IC_50_ NOX1 = 86.8 μM, NOX2 = 5.6 μM, NOX4 = 30.5 μM, and NOX5 = 39.2 μM).^42^ The 2-mercaptobenzoxazole and 2-oxazole moieties of these compounds act as outgoing groups, leaving a benzyl-triazolopyrimidine group covalently bound to the alkylated cysteine in the dehydrogenase domain (Figure 2A,B).^42^ Consistent with this mechanism, a cysteine-to-serine mutation makes the enzymes insensitive to the inhibitors. Having demonstrated the efficacy and mechanism of the VAS compounds, here, we describe a set of novel triazolopyrimidine-containing NOX inhibitors. Their enzymatic and cellular evaluations on the murine microglial cell model outline very promising activities. Critically, some of our new compounds are also active against human MAOB, thereby being able to ablate NOXs and MAOs, two of the most powerful enzymatic sources of ROS.

## Results

### Inhibitor Design

Compounds were designed from the VAS2870 scaffold using the following strategies (Figure 2C):

(i) removal of the leaving group to unveil any possible reversible, noncovalent binding of benzyl-triazolopyrimidine derivatives to NOXs (compounds **1** and **4**);
(ii) replacement of the 2-thiobenzoxazole with another leaving group (**2**–**3** and **5**–**6**);
(iii) substitutions of the leaving group with acrylamide moieties that are now established as pharmacologically powerful cysteine modifiers (**7a**–**7d**);^47^
(iv) introduction, at the *para* position of the benzyl group, of novel different cyclic and noncyclic tertiary amines aimed to obtain NOX2 selective inhibitors (**8a**–**b** and **9a**–**f**);
(v) introduction, at the *para* position of the benzyl group, of a propargylamine or propargylamide moieties, known to react with the isoalloxazine group of the FAD (**8b**–**9a**).

### Chemistry

**Scheme 1:**
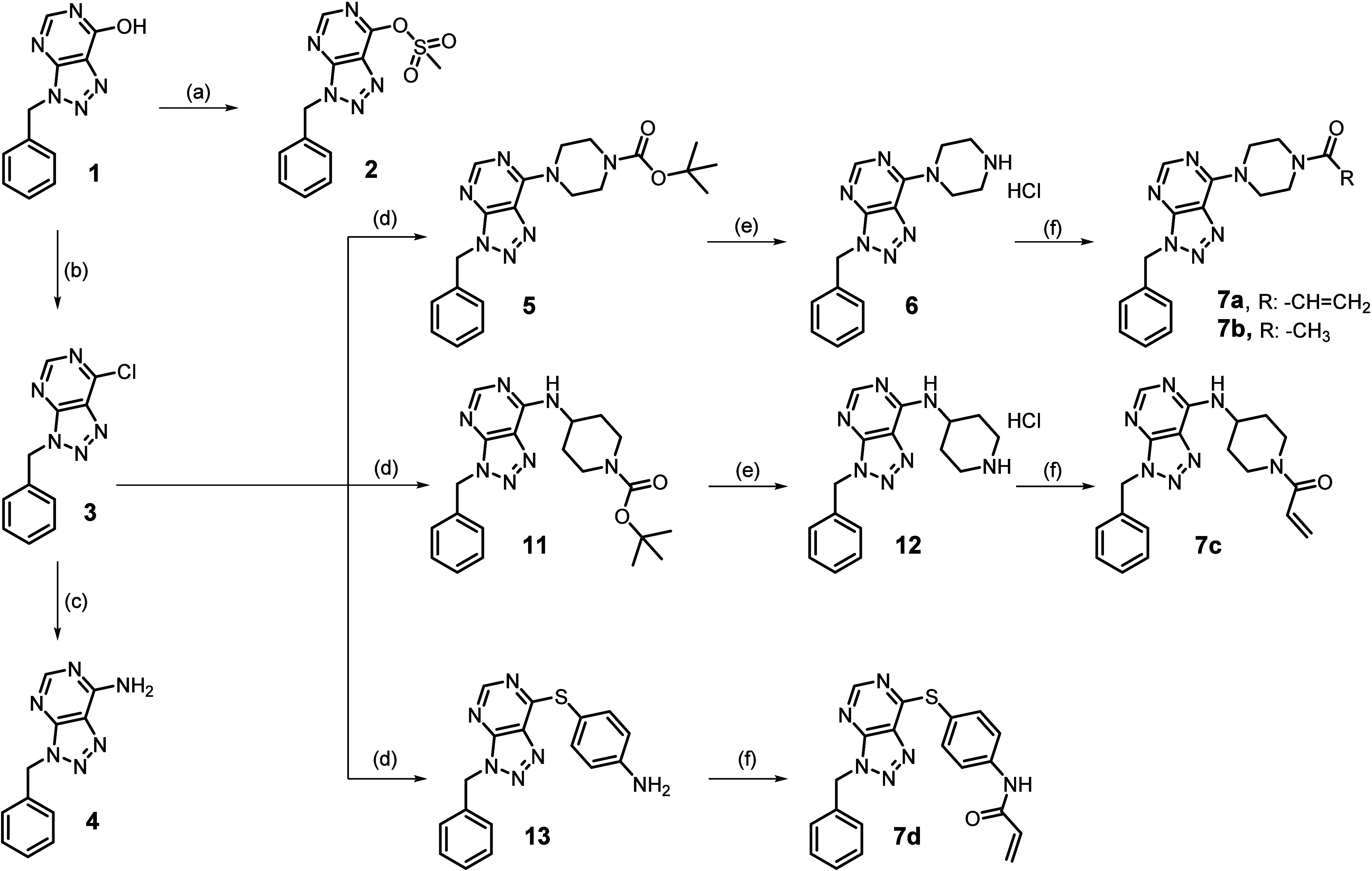
Synthesis of Compounds **2**–**6** and **7a**–**d** Reagents and conditions. (a) Methanesulfonyl chloride, TEA, dry DCM, 0 °C to RT, 10 min, yield: 33.5%; (b) SOCl_2_, dry CHCl_3_, dry DMF, 80 °C, 3 h, yield: 98.8%; (c) NH_3_ in MeOH, 1-butanol, 80 °C, 2 h, yield: 57.7%; (d) *tert*-butyl piperazine-1-carboxylate or *tert*-butyl 4-aminopiperidine-1-carboxylate or 4-aminobenzenethiol, TEA, dry EtOH, 0 °C to RT, 1 h, yield: 40.2–99.8%; (e) 4 N HCl in 1,4-dioxane, dry THF, 0 °C to RT, 3 h, yield: 75.5–77.9%; (f) acryloyl chloride or acetyl chloride, TEA, dry DCM, 0 °C to RT, 10 min, yield: 33.3–86.1%.

For the synthesis of compounds **2**–**6** and **7a**–**d**, the commercially available 3-benzyl-3*H*-[1,2,3]triazolo[4,5-*d*]pyrimidin-7-ol **1** was treated with methanesulfonyl chloride and triethylamine (TEA) in dry DCM, to obtain the mesylate derivative **2**. Alternatively, **1** was treated with thionyl chloride in dry chloroform and dry DMF to give the chloro derivative **3**, which was dissolved in 1-butanol and treated with 7 N ammonia solution in methanol to provide the amino derivative **4**. The compound **3** also underwent two different nucleophilic displacement reactions, with the *tert*-butyl piperazine-1-carboxylate and the *tert*-butyl 4-aminopiperidine-1-carboxylate, in the presence of TEA in dry ethanol to give compounds **5** and **11**, respectively. These same compounds were treated with 4 N hydrogen chloride in 1,4-dioxane/dry THF to afford the desired hydrochloride derivatives 3-benzyl-7-(piperazin-1-yl)-3*H*-[1,2,3]triazolo[4,5-*d*]pyrimidine **6** and 3-benzyl-*N*-(piperidin-4-yl)-3*H*-[1,2,3]triazolo[4,5-*d*]pyrimidin-7-amine **12**, which were isolated as colorless powders. Compounds **6** and **12** were treated with acryloyl chloride and TEA in dry DCM providing the final compounds **7a** and **7c**. The reaction of **6** with acetyl chloride, in the same conditions as before, led to **7b**. In addition, the chloro derivative **3** was treated with 4-aminothiophenol with TEA in dry ethanol to obtain derivative **13**, which was subjected to a reaction with acryloyl chloride and TEA in dry DCM to afford the final compound **7d** (Scheme 1).

**Scheme 2:**
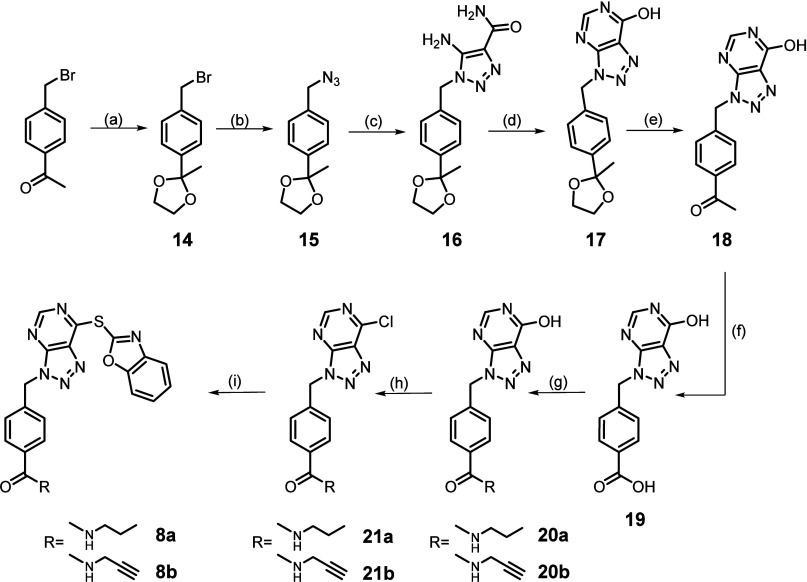
Synthesis of Compounds **8a**–**b** Reagents and conditions. (a) Ethylene glycol, PTSA, dry benzene, 145 °C, overnight, yield: 96.7%; (b) sodium azide, dry DMF, N_2_, overnight, yield: 74.8%; (c) cyanoacetamide, EtONa, dry EtOH, 80 °C, 3 h, yield: 70.4%; (d) ethyl formate, EtONa, dry EtOH, 80 °C, 2.5 h, yield: 99.8%; (e) 2 N HCl, EtOH, RT, 2 h, yield: 72.2%; (f) (i) Br_2_, glacial acetic acid, 50 °C, 4 h; (ii) 2N NaOH, 0 °C, 1 h; (iii) 2 N HCl, 0 °C, yield: 45.0%; (g) 1-propanamine or propargylamine, PyBOP, TEA, dry DMF, N_2_, RT, overnight, yield: 27.2–40.0%; (h) SOCl_2_, dry CHCl_3_, dry DMF, 40 °C, 48 h, yield: 90.0–98.1%; (i) 2-mercaptobenzoxazole, TEA, dry EtOH, 0°C, 30 min, yield: 30.0–32.2%.

For the synthesis of compounds **8a**–**b**, the commercially available 1-(4-(bromomethyl)phenyl)ethan-1-one was treated with ethane-1,2-diol and *p*-toluenesulfonic acid in dry benzene obtaining the ketal **14**, which was then treated with sodium azide in dry DMF overnight. The obtained 2-(4-(azidomethyl)phenyl)-2-methyl-1,3-dioxolane **15** was treated with sodium ethoxide and 2-cyanoacetamide in dry ethanol to provide the 5-amino-1-(4-(2-methyl-1,3-dioxolan-2-yl)benzyl)-1*H*-1,2,3-triazole-4-carboxamide **16**, which was treated with sodium ethoxide and ethyl formate in dry ethanol affording the 3-(4-(2-methyl-1,3-dioxolan-2-yl)benzyl)-3*H*-[1,2,3]triazolo[4,5-*d*]pyrimidin-7-ol derivative **17**. This last compound underwent an acidic hydrolysis with 2 N hydrogen chloride in ethanol to give the corresponding ketone **18** and subsequently subjected to bromination in glacial acetic acid, followed by a basic treatment with 2 N sodium hydroxide and then by an acidic one with 2 N hydrogen chloride, thus giving the 4-((7-hydroxy-3*H*-[1,2,3]triazolo[4,5-*d*]pyrimidin-3-yl)methyl)benzoic acid **19**. The reaction of this acid with benzotriazol-1-yloxytripyrrolidinophosphonium hexafluorophosphate (PyBOP) and TEA followed by 1-propylamine and propargylamine in dry DMF yielded the respective amides **20a** and **20b**. After the chlorination reaction of these derivatives with thionyl chloride in dry chloroform/DMF, the chloro derivatives **21a** and **21b** were treated with benzo[*d*]oxazole-2-thiol and TEA in dry ethanol to furnish the corresponding compounds **8a** and **8b** (Scheme 2).

**Scheme 3:**
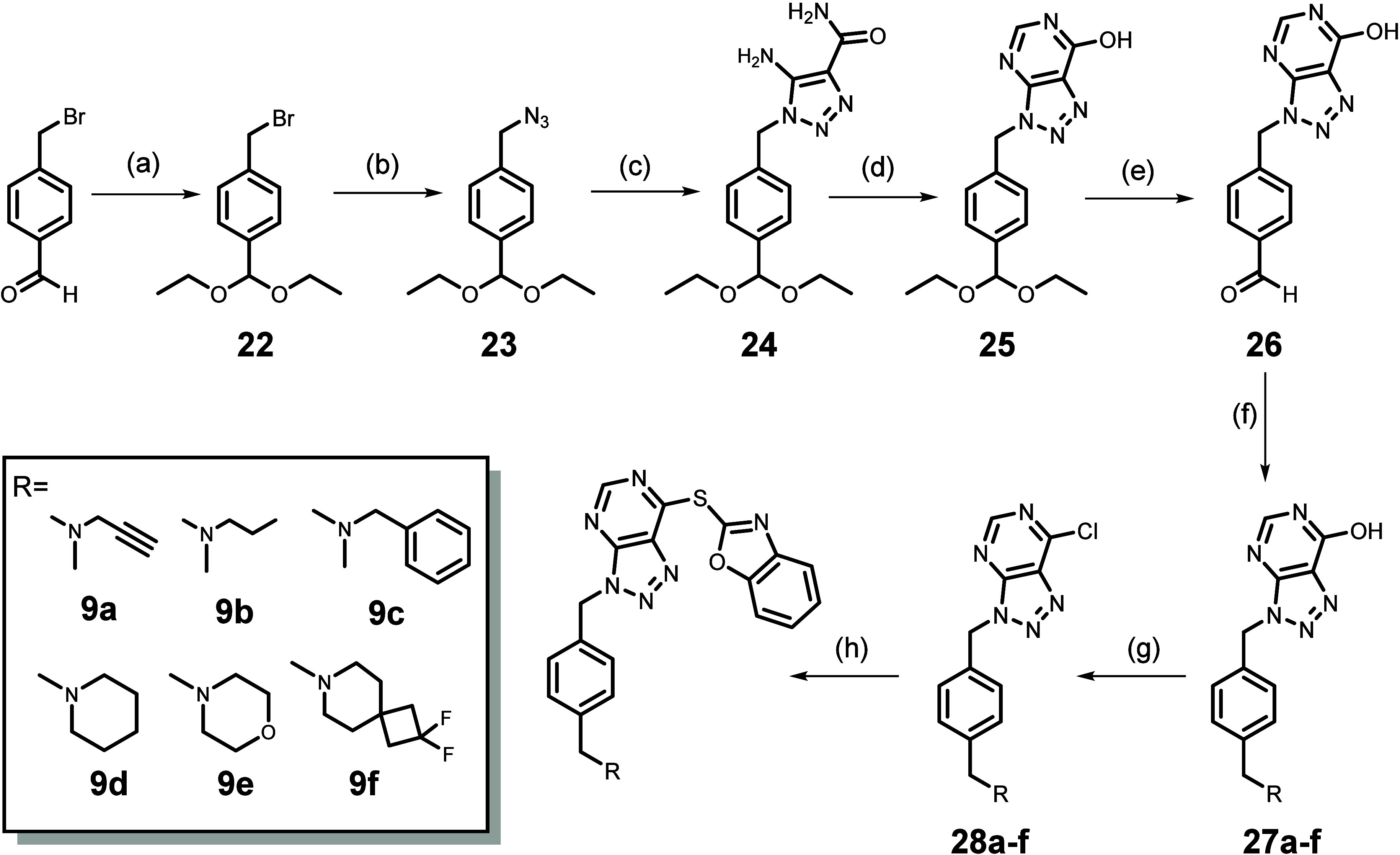
Synthesis of Compounds **9a**–**f** Reagents and conditions. (a) Triethyl orthoformate, Dowex 50W X8, dry EtOH, RT, 3 h, yield: 95.5%; (b) sodium azide, dry DMF, N_2_, RT, overnight, yield: 88.7%; (c) cyanoacetamide, EtONa, dry EtOH, 80 °C, 3 h, yield: 87.4%; (d) ethyl formate, EtONa, dry EtOH, 80 °C, 2.5 h, yield: 99.5%; (e) 2 N HCl, EtOH, 0 °C to RT, 2.5 h, yield: 97.7%; (f) (CH_3_COO)_3_BHNa, dry DMF, N_2_, RT, 2 h, yield: 61.1–89.9%; (g) SOCl_2_, dry DMF, dry CHCl_3_, 50 °C, overnight, yield: 38.2–75.3%; (h) 2-mercaptobenzoxazole, TEA, dry EtOH, 0 °C, 10 min, yield: 11.0–89.9%.

For the synthesis of compounds **9a**–**f**, the commercially available 4-(bromomethyl)benzaldehyde was treated with triethyl orthoformate in the presence of Dowex 50W X8 (HCR-W) in dry ethanol obtaining the 1-(bromomethyl)-4-(diethoxymethyl)benzene **22** that was treated with sodium azide in dry DMF. The obtained 1-(azidomethyl)-4-(diethoxymethyl)benzene derivative **23** was treated with sodium ethoxide and 2-cyanoacetamide in dry EtOH providing the 5-amino-1-(4-(diethoxymethyl)benzyl)-1*H*-1,2,3-triazole-4-carboxamide **24**. The latter was treated with sodium ethoxide and ethyl formate in dry ethanol to afford the 3-(4-(diethoxymethyl)benzyl)-3*H*-[1,2,3]triazolo[4,5-*d*]pyrimidin-7-ol derivative **25** that was subsequently subjected to acidic hydrolysis to remove the acetal group with 2 N hydrogen chloride in ethanol. The obtained aldehyde **26** underwent a reductive amination with sodium triacetoxyborohydride in dry DMF, using the appropriate amine, thus obtaining the hydroxy derivatives **27a**–**f**. These derivatives were subjected to a chlorination reaction with thionyl chloride in dry CHCl_3_/DMF, giving the chloro derivatives **28a**–**f**, which underwent nucleophilic displacement with benzo[*d*]oxazole-2-thiol and TEA in dry ethanol to yield the amine derivatives **9a**–**f** (Scheme 3).

### Initial Evaluation of the Biochemical Activity of Compounds **1**–**6**, **7a**–**d**, **8a**–**b**, and **9a**–**f**

First, we probed the role of the 2-thiobenzoxazole leaving group of VAS2870. A series of derivatives were synthesized and tested at 10 μM against the dehydrogenase domain of NOX5 from *Cylindrospermum stagnale* (*C. stagnale*, *Cs*NOX5; Figure 2B). With a sequence identity of 43% to the human NOX5, this protein is an experimentally convenient tool for the rapid and effective screening of antiNOX compounds because it can be expressed in *Escherichia coli* (*E. coli*), is stable, and its activity can be directly assayed by spectroscopically monitoring substrate consumption (Table 1).^40,42^ These experiments showed that the replacement of the 2-thiobenzoxazole with hydroxy (**1**), mesylate (**2**), chloro (**3**), or amino (**4**) groups essentially abolishes inhibition (note that **3** also gave a marginal 23.4% inhibition at 100 μM). Clearly, the inhibitory activity requires covalent attachment and the 2-thiobenzoxazole is critical for effective cysteine alkylation.

**Table 1:**
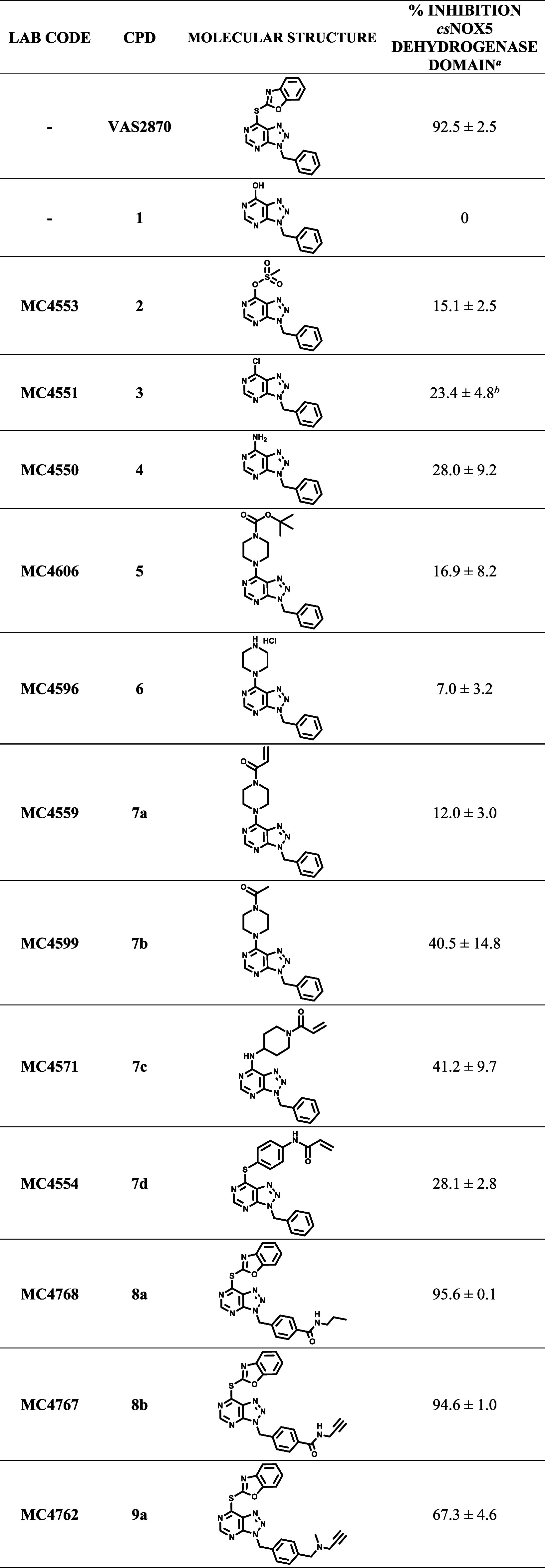
Inhibition the Dehydrogenase Domain of *cs*NOX5 by **1**–**9a**^c^

a 10 μM inhibitor.

b 100 μM inhibitor.

c Enzymatic activities were determined using the NADPH depletion assay with 1 μM purified protein and 250 μM NADPH.

For this reason, we next decided to replace the leaving group with a trapping one, by connecting an acrylamide portion to the triazolopyrimidine nucleus through a piperazine, 4-aminopiperidine, and 4-mercaptoaniline ring, thus obtaining **7a**, **7c**, and **7d**, respectively. Moreover, we also tested the synthetic piperazine intermediates **5** and **6** and the acetylpiperazine derivative **7b** as nontrapping agents (e.g., putative negative controls). All of these compounds proved to be far less powerful than VAS2870 (Table 1). Notably, although piperidine acrylamide **7c** displayed the highest inhibition percentage, no covalent binding to the active-site cysteine was observed (Figure S1). Moreover, the acrylamide-lacking **7b** displayed a potency very similar to that of **7c**, further demonstrating that our “Cys-trapping” plan of action was ineffective.

**Figure 3:**
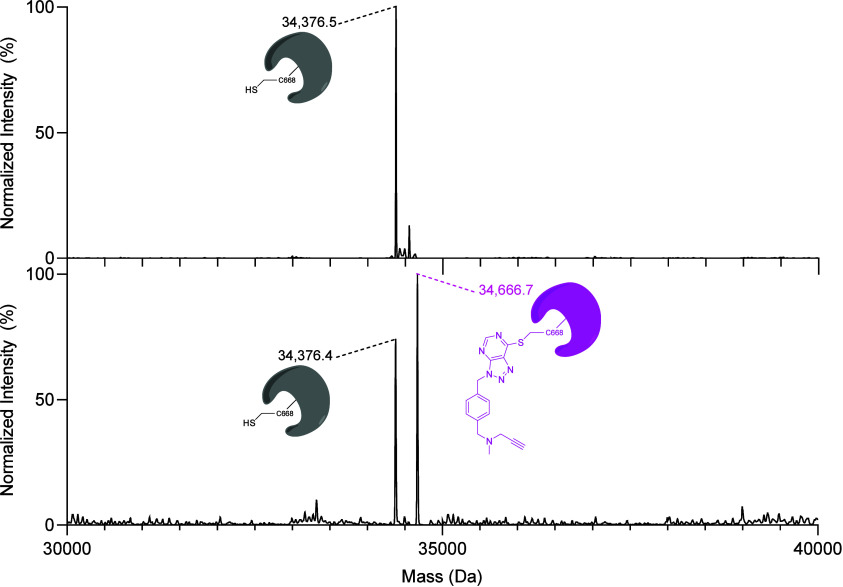
Intact protein mass spectrometry of *cs*NOX5 dehydrogenase after the reaction with **9a**. Normalized deconvoluted mass spectra of the DMSO control and the inhibited protein are shown in the upper and lower panel, respectively. The mass difference between the inhibited and DMSO-treated protein peaks is 290.3 (theoretical 290.34 Da), matching the value expected for the cysteine adduct with a mass error of −4.04 ppm (Figure 2A). This result implies that the inhibitor propargyl moiety does not form a covalent adduct with the flavin.

From the above results, we decided to maintain the 2-thiobenzoxazole leaving group and act on the triazolopyrimidine nucleus. Our approach was also intended to explore the feasibility of dual, simultaneous targeting of the active-site cysteine and nearby FAD (Figure 2B). As an additional benefit, such an approach could potentially deliver dual inhibitors, able to bind other flavoenzymes in addition to NOXs. We prepared the *p*-propargylamide (**8b**) and *p*-propargylamine (**9a**) derivatives of VAS2870 as well as saturated compound **8a**, a negative control for FAD covalent binding. The assays against the dehydrogenase domain of *cs*NOX5 highlighted that all three new compounds retained the same high potency of VAS2870. These data substantiated the notion that the presence of 2-thiobenzoxazole as the leaving group is required for strong inhibition (Table 1). Indeed, the ESI-qTOF-HRMS analysis of the intact *cs*NOX5 dehydrogenase preincubated with compound **9a** exhibited the expected 290 Da increase in molecular weight, thus validating the covalent binding to the protein (Figure 3). Instead, mass spectrometry provided no evidence for modification of FAD that is indeed not retained by the inhibited protein in gel filtration. We therefore conclude that the propargyl group does not covalently bind to the FAD’s isoalloxazine ring of NOXs.

### Inhibition of Human NOXs and Enzyme Selectivity

Based on these encouraging results on *cs*NOX5 dehydrogenase, we tested the new compounds toward human NOX1, NOX2, NOX4, and NOX5 using membrane preparations obtained from their respective NOX-overexpressing cells (Table 2). We found **8b** to be active on NOX2 (IC_50_ = 17.3 μM) and, to a lower extent, on NOX4 (IC_50_ = 57.9 μM) while being inactive on NOX1 and NOX5. Critically, **9a**, bearing a propargylamine group, proved to be 2-fold more powerful than VAS2870, with an IC_50_ value against NOX2 in the high nanomolar range (IC_50_ = 0.567 μM). It also displayed some degree of selectivity over the other NOX isoforms, especially when compared with NOX1 (IC_50_ = 22.0 μM) and NOX4 (IC_50_ = 42.0 μM) and to a lesser extent over NOX5 (IC_50_ = 5.2 μM; selectivity index NOX2/NOX1 = 39, NOX2/NOX4 = 74, and NOX2/NOX5 = 9). For comparison, VAS2870 is equally potent against NOX2 and NOX5 (IC_50_ = 1.1 and 1.8 μM, respectively) while displaying some degree of selectivity mainly against NOX1. Next, given that **9a** was the most potent NOX2 inhibitor, we designed and synthesized the other five amine analogues (**9b**–**f**). Only the cyclic amines piperidine (**9d**) and morpholine (**9e**) maintained a single-digit micromolar inhibition potency and selectivity for NOX2 and NOX4 while being less potent than **9a** against NOX2 (Table 2). Although there are limitations related to varying expression levels and protein stabilities, these results demonstrate that achieving NOX-selective covalent inhibition is a viable approach.

**Table 2:**
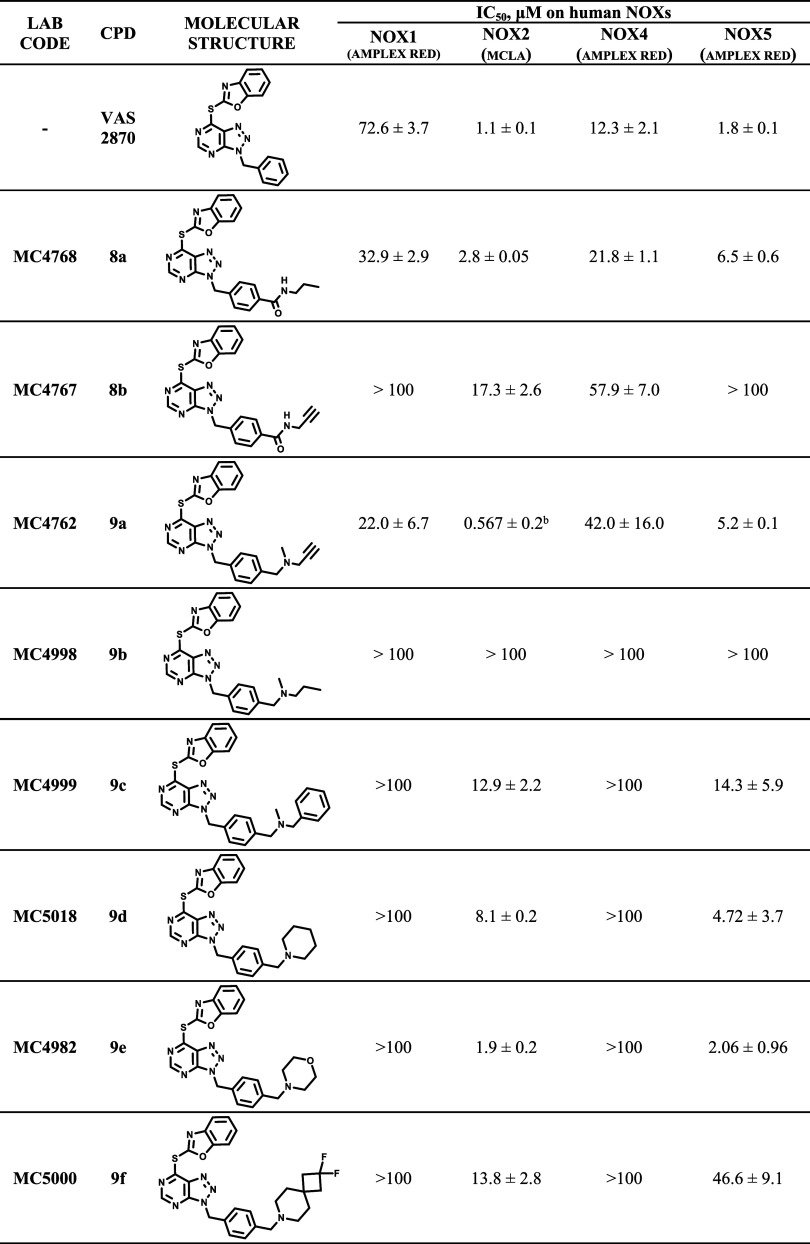
IC_50_ Values for Compounds **8a**–**b** and **9a**–**f** against Membrane-Embedded Human NOXs^a^

a Data are shown in Figures S2–S5 and S11 in the Supporting Information. NOX1, NOX4, and NOX5 activities were determined using the Amplex Red/horseradish peroxidase coupled assay and 40 μM NADPH. NOX2 activities were determined using the MCLA assay and 240 μM NADPH.

b The IC_50_ value measured with the cytochrome c assay is 0.770 μM.

The data were further corroborated by biochemical and cellular assays using highly purified, activated NOX2 and intact NOX2-overexpressing PLB-985 cells (Table 3). Compounds **8a** and **8b** showed lower potency than VAS2870 yet manifested a good inhibition with IC_50_ values in the single- (**8a**) and double-digit (**8b**) micromolar range. Above all, **9a** confirmed to be over 20-fold more potent than VAS2870 on the purified NOX2 (IC_50_ = 0.155 μM vs 3.5 μM) and 2-fold more potent against NOX2 *in cellulo* (IC_50_ = 0.135 μM vs 0.304 μM).

**Table 3:**
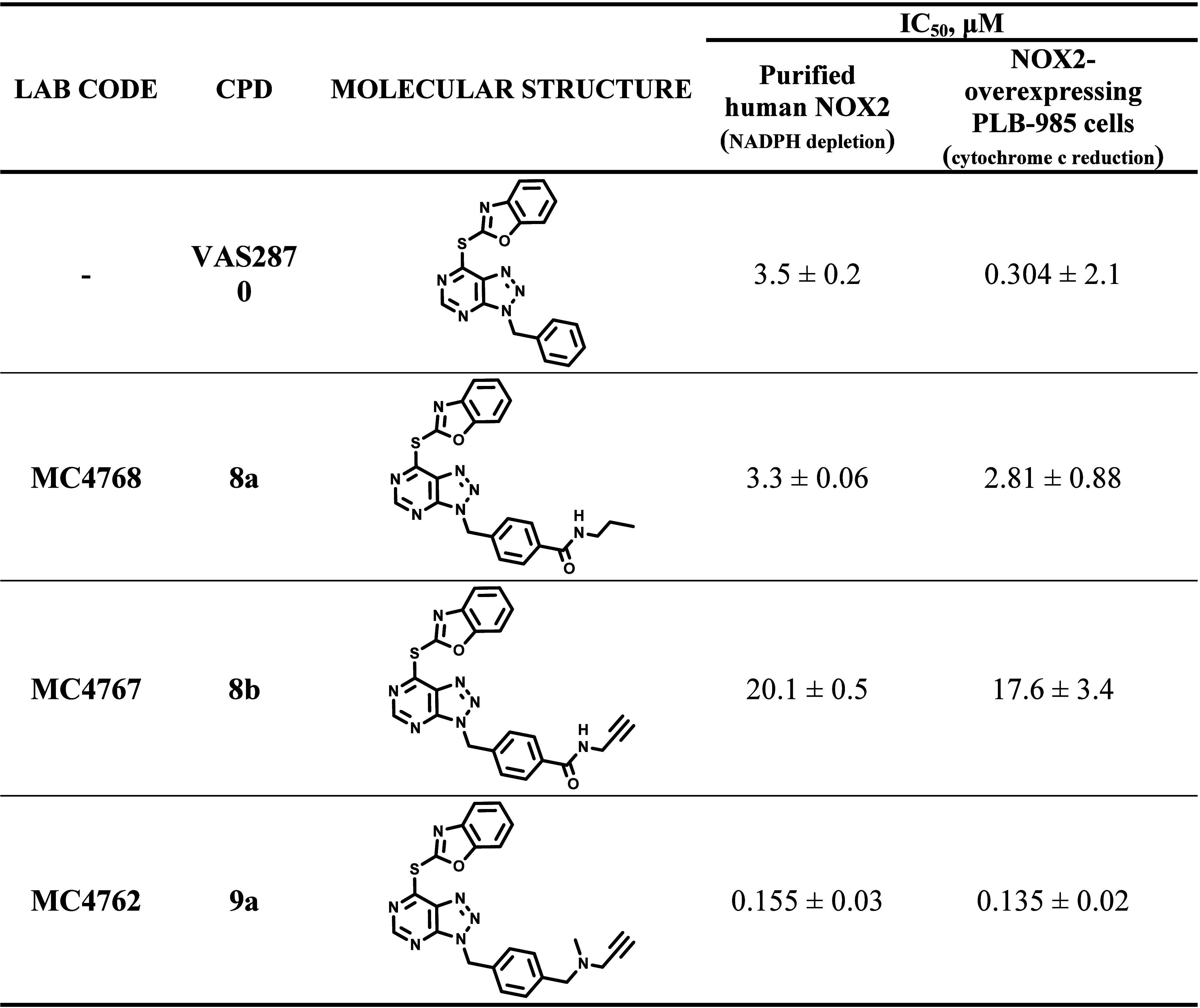
Inhibition by **8a**–**b** and **9a** on Purified NOX2 and on NOX2-Overexpressing PLB-985 Cells^a^

a Data are shown in Figures S6 and S7 in the Supporting Information. Enzymatic activities were determined using the NADPH depletion assay with 200 nM NOX2 and 240 μM substrates. Cellular NOX2 activities were followed by cytochrome c (200 μM) reduction.

### VAS2870 and **9a** Bind to Activated NOX2

**Figure 4:**
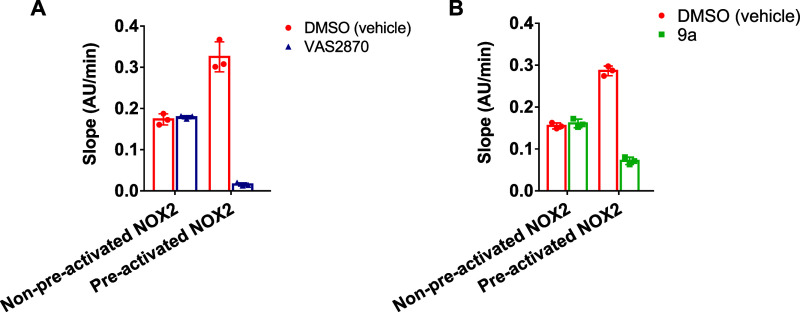
The NOX2 active site is accessible to the ligand only upon protein preactivation. *In vitro* activity (cytochrome c reduction assay) of isolated NOX2-overexpressing membranes after 60 min of incubation with VAS2870 (A) and **9a** (B) in the absence or presence of the cytosolic activators followed by ligand washout and enzyme activation by the addition of a fresh p47^phox^/p67^phox^/Rac1 complex.

The experimental NOX2 structures revealed that enzyme activation by the cytosolic p47^phox^/p67^phox^/Rac1 complex involves a conformational change in the dehydrogenase domain.^48^ This feature raised the possibility that VAS binding might selectively occur only in the resting or activated states of the enzyme. To address this issue, we devise three-step experiments: (i) NOX2-overexpressing cell membranes were incubated with an excess of VAS2870 or **9a** in the absence (resting state) or presence (activated state) of the cytosolic activators; (ii) the unbound inhibitor was then washed out; finally, (iii) the activity of NOX2 was measured after adding fresh cytosolic activators. Results show that VAS inhibitors are retained only by the activated, p47^phox^/p67^phox^/Rac1-bound form of NOX2. The inhibitors are instead unable to alkylate NOX2 in its resting state, which is normally activated after washout of the inhibitor (Figure 4A,B). From these results, we conclude that the VAS compounds are conformationally selective and sense the activation state of the protein.

### Compound **9a** Is a Submicromolar, Covalent Inhibitor of Human MAOB

With their acute neurotransmitter- and cathecolamine-oxidizing activities, MAOA and MAOB are among the principal enzymatic sources of ROS and are implicated in various neurodegenerative diseases. A feature common to most MAO inhibitors is a single- or two-ring aromatic scaffold decorated by a warhead that forms a covalent adduct with the flavin.^26−28^ As **8b** and **9a** are endowed with these chemical features, we reasoned that they might inhibit human MAOs.^49^ The hypothesis proved to be correct: activity assays on the purified enzymes revealed that **9a** is a strong and selective inhibitor of human MAOB (IC_50_ = 0.182 μM vs IC_50_ = 59.0 μM for MAOA; selectivity index MAOA/MAOB = 465) though 10-fold weaker than rasagiline (Table 4). Moreover, the shift in absorbance of the MAOB-bound flavin absorbance spectrum (from two peaks at 370 and 456 nm to a single peak at 410 nm) clearly indicated formation of a covalent *N*-methyl-*N*-propargylamine-flavin adduct, which is typical for the propargyl-containing MAO inhibitors (Figure S10). Importantly, VAS2870 and **8a**, both lacking the propagylamine, are ineffective against MAOs (data not shown).

**Table 4:**
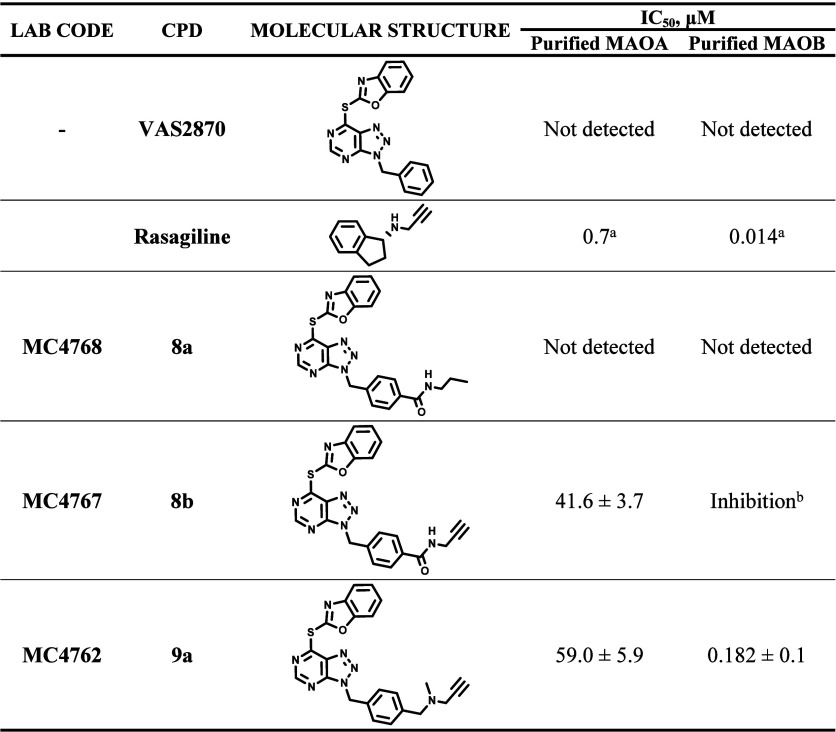
MAOA and MAOB Inhibition^c^

a Data are from ref (50).

b At high concentrations, the compound slightly interferes with the assay, preventing the determination of the exact IC_50_ value.

c Data are shown in Figures S8 and S9 in the Supporting Information. Enzymatic activities were determined using the Amplex Red/horseradish peroxidase coupled assay with 70 nM MAOA or 10 nM MAOB and 1 mM substrates.

However, to investigate on the potential side effects of these compounds possibly elicited by the alkylation of thiol-containing metabolites and/or other protein targets, we evaluated the capability of **9a** to bind glutathione, as already shown by VAS2870,^51^ or cysteine-containing human myoglobin and bovine albumin. The results have clearly proven that even though **9a** was able to form an adduct with glutathione (Figure S12), it did not bind to neither protein (Figures S13 and S14).

### Crystal Structure of Compound **9a** in Complex with MAOB

**Figure 5:**
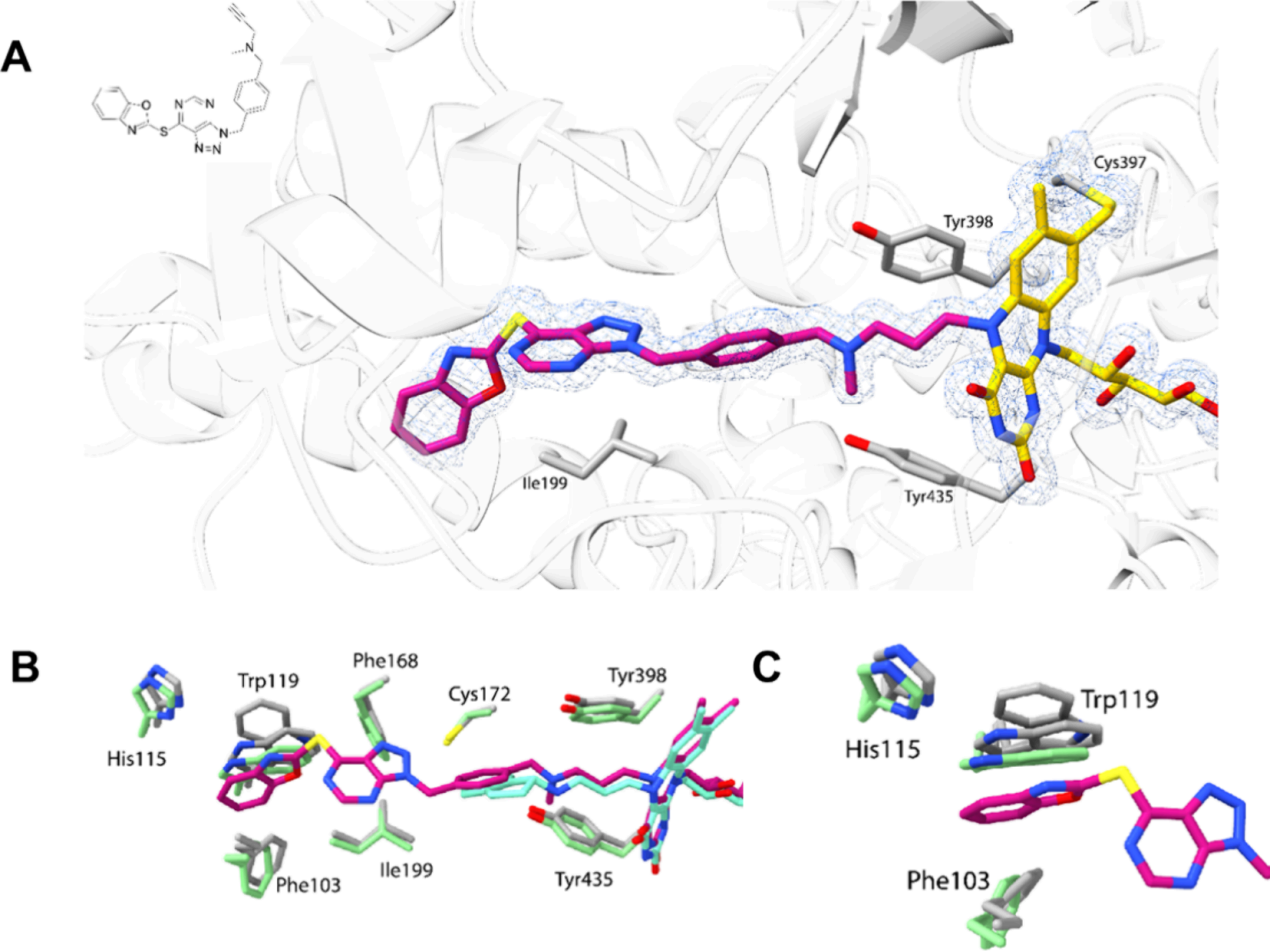
Crystal structure of human MAOB in complex with the inhibitor **9a** at a 1.4 Å resolution. (A) Close-up view of the active site of chain B. Active-site residues, FAD, and the inhibitor are shown with gray, yellow, and magenta carbons; oxygen is in red, nitrogen in blue, and sulfur in light yellow. The backbone trace is shown as a light gray transparent ribbon. The refined 2F_O_–F_C_ electron density for **9a** and FAD is shown as a blue mesh and is contoured at 1.5σ. (B) Superposition with MAOB in complex with rasagiline, a clinically used anti-Parkinson drug (PDB 1S2Q). The FAD-**9a** adduct is shown in magenta with protein residues in gray. The FAD-rasagiline adduct is shown in cyan with protein residues in light green. (C) Zoomed-in view of the terminal benzoxazole ring of **9a** and its surrounding amino acids (Table S3 in the Supporting Information).

Crystallographic studies were carried out to elucidate the binding mode of **9a** to human MAOB at a resolution of 1.4 Å (Table S3). The *N*-methyl-propargylamine moiety forms a delocalized double bond adduct with the N5 of the flavin and lies between the “aromatic sandwich” (Tyr398–Tyr435 pair; Figure 5A). The *para* substitution of the central aromatic ring perfectly fits the narrow MAOB’s active site. As typically observed in MAOB structure-bound bulky elongated inhibitors, the gating Ile199 is in an open conformation, allowing binding of the terminal benzoxazole moiety.^52^ Indeed, superposition with the structure in complex with rasagiline, a clinically used propargylamine-based anti-Parkinson’s disease drug, shows an identical active-site conformation, except for some differences at the entrance cavity (Phe103, His115, and Trp119; Figure 5B,C). Collectively, these data demonstrate that **9a** is a dual, NOX2 and MAOB, inhibitor with balanced IC_50_ values against the two targets.

### Dual NOX2 and MAOB Inhibition Effects in the Microglia BV2 Cell Line

**Figure 6:**
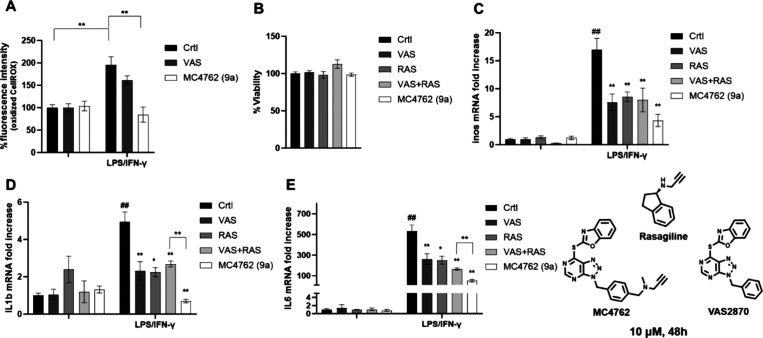
Activity of NOX and MAOB inhibitors in BV2 cells before and after LPS/IFN-γ-driven proinflammatory stimuli and 48 h compound (10 μM) treatment. (A, B) ROS levels and cell viability. (C–E) qRT-PCR analysis for the indicated transcripts. CTRL (control) represents the cells treated with the vehicle (DMSO). The values are expressed as fold of expression versus the control (arbitrary value = 1) and shown as means ± SD. Statistically significant differences are reported (*p < 0.05; **p < 0.01) for four independent experiments.

NOX2 regulates cytokine induction in neuroinflammation processes. Its deletion resulted in clinical improvement of multiple sclerosis symptoms by preventing astrocyte activation and impairing mRNA expression of proinflammatory cytokines such as interleukin-1beta (IL-1β), interleukin-6 (IL-6), and monocyte chemoattractant protein-1 in both the striatum and motor cortex.^53^ In a very recent study, the NOX2-specific deletion was shown to effectively attenuate retinal oxidative stress, immune dysregulation, internal blood–retinal barrier injury, and neurovascular unit dysfunction. In the same work, NOX2-dependent ROS were found to drive proinflammatory signaling, activate the ERK1/2 pathway, and contribute to the shift of microglia activation to a proinflammatory M1 phenotype, by triggering a neuroinflammatory flare.^54^ Likewise, a variety of inflammatory models have been reported in which MAO inhibition afforded a reduction of cytokine expression. For instance, in an epithelial cell culture model, LPS-induced IL-6 and IL-1β cytokine expressions were downregulated by MAOB inhibition through the impairment of cAMP-PKA/EPAC signaling.^55^ Given these findings, we reasoned that **9a**, with its dual activity, may represent an insightful chemical tool for studying the role of NOX2^53^ and MAOB activities in inflammatory or neurodegenerative diseases. We focused on proinflammatory microglia as it plays a detrimental role in the progression of several neurodegenerative diseases.^56,57^ The experiments were performed with VAS2870, rasagiline, and **9a**. We first investigated ROS production and activity in BV2 cells (a murine microglial model) measuring changes in fluorescence intensity due to CellROX Deep Red reagent oxidization and found that, upon LPS/IFN-γ stimulation, all three compounds (10 μM) were able to decrease the ROS levels with negligible effects on cells viability (Figure 6A,B). Notably, the strongest ROS-dumping activity was exerted by **9a**, even if it has approximately the same potency of VAS2870 as the NOX2 inhibitor and is 10-fold weaker than rasagiline as the MAOB inhibitor (Tables 3 and 4). Next, after LPS/IFN-γ-driven proinflammatory stimuli and 48 h of compound treatment, the effects on the mRNA expression of inducible nitric oxide synthase (iNOS), IL-1β, and IL-6 mRNA were measured (Figures 6C, 6D, and 6E, respectively). Again, **9a** proved to be the most effective as it elicits a substantial decrease in the mRNA levels of all three cytokines: iNOS (over 10-fold), IL-1β (4-fold), and IL-6 (4-fold), being more potent than the single target inhibitors and their combination. However, these effects were negligible without NOX2 inhibitor (both VAS2870 and **9a**) pretreatment (Figure S16).

Altogether, our data demonstrated that our dual NOX2/MAOB inhibitor **9a** is able to shape the microglial proinflammatory phenotype, with a greater effect than VAS2870, rasagiline, and their combination, reducing the impact of LPS/IFN-γ stimulation *in vitro*.

## Discussion and Conclusions

As NOXs have the specific role of ROS generation, their overexpression or deregulation can lead to various degenerative and hyperproliferative pathological conditions. The redox reactivity of many chemicals has confounded the search and validation of NOX inhibitors that are often confused with ROS-scavenging compounds. The known validated inhibitors interfere with p47^phox^ binding to NOX2 (indirect inhibition) or target the NADPH binding site in the NOXs’ dehydrogenase domain. Reported 20 years ago,^45^ VAS2870 and its analogues are covalent inhibitors that alkylate an active-site cysteine. In this work, we find that replacement of their mercaptobenzoxazole leaving group with an acrylamide cysteine modifier abolishes inhibition. Their benzyl-triazolopyrimidine group instead tolerates modifications, offering valuable opportunities for crafting more isoform-specific inhibitors or compounds endowed with additional activities. Following this route, we generated compounds that discriminate among NOX enzymes and gain in potency. For instance, **8b** is active only on NOX2 and NOX4 whereas **9a** is potent mainly against NOX2. An interesting feature revealed by our studies is that the VAS compounds are conformationally selective, as they covalently attack NOX2 only when it is in the activated state. We speculate that the cysteine targeted by these compounds is only accessible and/or adequately reactive when the enzyme is stabilized in the catalytically active form. The operability of the triazolopyrimidine scaffold of the VAS inhibitors can enable the design of dual-activity inhibitors. This concept was proven by **9a**, a submicromolar inhibitor of both NOX2 and MAOB. Its activity in a cellular model of neuroinflammation provides proof-of-principle data that inhibiting two strong enzymatic sources of ROS can be beneficial. Moreover, dual-activity compounds such as **9a** can be most valuable for the interrogation of the cellular and biochemical roles selectively played by ROS-generating enzymes as opposed to nonspecific and nonlocalized ROS sources. However, considering the potential side effects of these compounds due to the alkylation of thiol-containing metabolites but not of cysteine-containing proteins, our research group will undertake studies addressed to the design and development of more specific VAS2870-derived molecules and novel chemical scaffolds as NOX inhibitors endowed with better drug-like properties.

## Experimental Section

### Chemistry

Melting points were determined on a Buchi 530 melting point apparatus and are uncorrected. ^1^H NMR and ^13^C NMR spectra were recorded at 400 MHz on a Bruker AC 400 spectrometer; chemical shifts are reported in δ (ppm) units relative to the internal reference tetramethyl silane (Me_4_Si). All compounds were routinely checked by TLC, ^1^H NMR, and ^13^C NMR spectra. TLC was performed on aluminum-backed silica gel plates (Merck DC, Alufolien Kieselgel 60 F254) with spots visualized by UV light. All solvents were of reagent grade and, when necessary, were purified and dried by standard methods. The concentration of solutions after reactions and extractions involved using a rotary evaporator operating at a reduced pressure of ca. 20 Torr. Organic solutions were dried over anhydrous sodium sulfate. Elemental analysis and HPLC analysis were used to determine the purity of the described compounds, which is >95%. Analytical results are within ±0.40% of the theoretical values (Tables S1 and S2 in the Supporting Information). All chemicals were purchased from Sigma-Aldrich, Milan (Italy), Alfa Aesar, Karlsruhe (Germany), Fluorochem, Manchester (UK), or BLD-Pharma, Kaiserslautern (Germany) and were of the highest purity.

### General Procedure for the Synthesis of Azido Derivatives **15** and **23**

#### Example: Synthesis of 2-(4-(Azidomethyl)phenyl)-2-methyl-1,3-dioxolane (**15**)

To a solution of 2-(4-(bromomethyl)phenyl)-2-methyl-1,3-dioxolane (2.18 g, 8.48 mmol) in dry DMF, sodium azide (1.10 g, 169.6 mmol) was added under a nitrogen flow. After stirring at RT for 14 h, water was added, and the resulting reaction mixture was extracted with diethyl ether (3 × 20 mL). The organic extracts were collected, washed with brine, dried with sodium sulfate, and concentrated under reduced pressure. The remaining residue was purified by column chromatography (SiO_2_, eluted with ethyl acetate/*n*-hexane 1:20) to provide the pure compound **15**.

Mp liquid; yield: 74.8%; ^1^H NMR (CDCl_3_, 400 MHz, δ; ppm): 1.68 (s, 3H, −C*H*_*3*_), 3.80 (t, 2H, *J* = 2 Hz, ketal protons), 4.07 (t, 2H, *J* = 2 Hz, ketal protons), 4.37 (s, 2H, −C*H*_*2*_−), 7.32 (d, 2H, *J* = 8 Hz, aromatic protons), 7.53 (d, 2H, *J* = 8 Hz, aromatic protons).

### General Procedure for the Synthesis of the Amino Carboxamide Derivatives **16** and **24**

#### Example: Synthesis of 5-Amino-1-(4-(diethoxymethyl)benzyl)-1*H*-1,2,3-triazole-4-carboxamide (**24**)

To a solution of sodium ethoxide, the obtained solubilizing sodium (298.37 mg, 12.97 mmol) in dry EtOH was added with the previously synthesized intermediate 1-(azidomethyl)-4-(diethoxymethyl)benzene (**23**) (1.11 g, 6.48 mmol). After stirring at 80 °C for 1 h and 30 min, EtOH was evaporated, and the reaction was quenched with ammonium chloride saturated solution. The pure white precipitate **24** was filtered off.

Mp 240–242 °C, recrystallization solvent: acetonitrile/methanol; yield: 73.5%; ^1^H NMR (DMSO, 400 MHz, δ; ppm): 1.23 (t, 6H, *J* = 4 Hz, −O–CH_2_–C*H*_*3*_), 3.65 (m, 4H, −O–C*H*_*2*_–CH_3_), 5.47 (s, 2H, −C*H*_*2*_−), 5.49 (s, 1H, −C*H*−), 7.27 (d, 2H, *J* = 6 Hz, aromatic protons), 7.38 (d, 2H, *J* = 6 Hz, aromatic protons), 7.92 (s, 2H, −CON*H*_*2*_), 8.59 (s, 2H, −N*H*_*2*_).

### General Procedure for the Synthesis of the Triazolo-pyrimidinol derivatives **17** and **25**

#### Example: Synthesis of 3-(4-(2-Methyl-1,3-dioxolan-2-yl)benzyl)-3*H*-[1,2,3]triazolo[4,5-*d*]pyrimidin-7-ol (**17**)

To a solution of sodium ethoxide, the obtained solubilizing sodium (515.24 mg, 22.4 mmol) in dry EtOH was added with ethyl formate (1.44 mL, 17.92 mmol) and the previously synthesized 5-amino-1-(4-(2-methyl-1,3-dioxolan-2-yl)benzyl)-1*H*-1,2,3-triazole-4-carboxamide (**16**) (1.36 g, 4.48 mmol). After stirring at 80 °C for 3 h, EtOH was evaporated, and the reaction was quenched with ammonium chloride saturated solution. The pure white precipitate **17** was filtered off.

Mp >250 °C, recrystallization solvent: methanol; yield: 98.8%; ^1^H NMR (DMSO, 400 MHz, δ; ppm): 1.15 (s, 3H, −C*H*_*3*_), 3.66 (t, 2H, *J* = 2 Hz, ketal protons), 3.96 (t, 2H, *J* = 2 Hz, ketal protons), 5.75 (s, 2H, −C*H*_*2*_−), 7.32 (d, 2H, *J* = 8.4 Hz, aromatic protons), 7.40 (d, 2H, *J* = 8.4 Hz, aromatic protons), 7.88 (s, 1H, −OH), 8.27 (s, 1H, pyrimidine proton).

#### Synthesis of Mesylate Derivative **2**, MC4553

To a solution of commercially available 3-benzyl-3*H*-[1,2,3]triazolo[4,5-*d*]pyrimidin-7-ol (40 mg, 0.17 mmol) in dry DCM, TEA (0.04 mL, 0.26 mmol) and, at 0 °C, methanesulfonyl chloride (0.02 mL, 0.25 mmol) were added. The reaction was stirred at RT for 10 min. DCM was evaporated, and the resulting reaction mixture was purified by column chromatography (SiO_2_, eluted with ethyl acetate/*n*-hexane 1:1.5) to provide the pure compound **2**.

Mp 170–171 °C, recrystallization solvent: toluene/acetonitrile; yield: 33.5%; ^1^H NMR (CDCl_3_, 400 MHz, δ; ppm): 3.69 (s, 3H, −CH_3_), 5.73 (s, 2H, −CH_2_−), 7.36 (t, 3H, *J* = 2 Hz, aromatic protons), 7.44 (d, 2H, *J* = 2 Hz, aromatic protons), 8.70 (s, 1H, pyrimidine protons).

### General Procedure for the Synthesis of the Chloro Derivatives **3**, **21a**, **21b**, and **28a**–**f**

#### Example: Synthesis of 3-Benzyl-7-chloro-3*H*-[1,2,3]triazolo[4,5-*d*]pyrimidine, **3**, MC4551

To a solution of commercially available 3-benzyl-3*H*-[1,2,3]triazolo[4,5-*d*]pyrimidin-7-ol (50 mg, 0.22 mmol) in dry CHCl_3_, dry *N*,*N*-DMF (0.05 mL, 0.26 mmol) and, at 0 °C, SOCl_2_ (0.2 mL, 0.24 mmol) were added. The reaction was stirred at 80 °C for 3 h. After this time, the reaction was quenched with sodium bicarbonate and extracted with CHCl_3_ (3 × 20 mL). The organic extracts were collected, washed with brine, dried with sodium sulfate, and concentrated under reduced pressure. The resulting reaction mixture was purified by column chromatography (SiO_2_, eluted with ethyl acetate/*n*-hexane 1:1) to provide pure compound **3**.

Mp 88–91 °C, recrystallization solvent: cyclohexane/toluene; yield: 98.8%; ^1^H NMR (CDCl_3_, 400 MHz, δ; ppm): 5.82 (s, 2H, −CH_2_−), 7.30 (t, 3H, *J* = 2 Hz, aromatic protons), 7.40 (d, 2H, *J* = 2 Hz, aromatic protons), 8.86 (s, 1H, pyrimidine protons).

#### Synthesis of 3-Benzyl-3*H*-[1,2,3]triazolo[4,5-*d*]pirimidin-7-amina, **4**, MC4550

To a solution of 3-benzyl-7-chloro-3*H*-[1,2,3]triazolo[4,5-*d*]pyrimidine (**9**) (117 mg, 0.47 mmol) in 1-butanol, a solution of methanolic ammonia 7 N (0.68 mL, 4.76 mmol) was added. The reaction was stirred at 110 °C for 2 h. The reaction was quenched with distilled water, and the pure white precipitate **4** was filtered off.

Mp 255–256 °C, recrystallization solvent: methanol; yield: 57.7%; ^1^H NMR (DMSO, 400 MHz, δ; ppm): 5.76 (s, 2H, −CH_2_−), 7.33–7.34 (m, 5H, aromatic protons), 8.25 (s, 1H, −NH_2_), 8.31 (s, 1H, pyrimidine protons), 8.45 (s, 1H, −NH_2_).

### General Procedure for the Synthesis of the **5**, **11**, and **13** Derivatives

#### Example: Synthesis of 4-((3-Benzyl-3*H*-[1,2,3]triazolo[4,5-*d*]pyrimidin-7-yl)thio)aniline (**13**)

To a solution of 3-benzyl-7-chloro-3*H*-[1,2,3]triazolo[4,5-*d*]pyrimidine (**3**) (23.5 mg, 0.09 mmol) in dry EtOH, TEA (0.01 mL, 0.09 mmol) and, at 0 °C, 4-aminothiophenol (11.97 mg, 0.09 mmol) were added. The reaction was stirred at RT for 1 h. After this time, the reaction was quenched with brine. The white precipitate **13** was filtered off.

Mp 178–180 °C, recrystallization solvent: acetonitrile; yield: 99.8%; ^1^H NMR (DMSO, 400 MHz, δ; ppm): 5.72 (s, 2H, −NH_2_), 5.96 (s, 2H, −CH_2_−), 6.72 (d, 2H, *J* = 8.4 Hz, aromatic protons), 7.31 (d, 2H, *J* = 8.8 Hz, aromatic protons), 7.38–7.40 (m, 5H, aromatic protons), 8.85 (s, 1H, pyrimidine protons).

#### **5**, MC4606, *tert*-Butyl 4-(3-benzyl-3*H*-[1,2,3]triazolo[4,5-*d*]pyrimidin-7-yl)piperazine-1-carboxylate

Mp 133–136 °C, recrystallization solvent: toluene; yield: 66.7%; ^1^H NMR (DMSO, 400 MHz, δ, ppm): 1.44 (s, 9H, −C–(CH_3_)_3_), 3.54 (d, 4H, *J* = 19.2, piperazine protons), 4.01 (s, 2H, piperazine protons), 4.56 (s, 2H, piperazine protons), 5.80 (s, 2H, −CH_2_−), 7.28–7.37 (m, 5H, aromatic protons), 8.42 (s, 1H, pyrimidine proton).

### General Procedure for the Synthesis of the Acrylamide Derivatives **7a**, **7c**, and **7d**

#### Example: Synthesis of *N*-(4-((3-Benzyl-3*H*-[1,2,3]triazolo[4,5-*d*]pyrimidin-7-yl)thio)phenyl)acrylamide, **7d**, MC4554

To a solution of 4-((3-benzyl-3*H*-[1,2,3]triazolo[4,5-*d*]pyrimidin-7-yl)thio)aniline (**13**) (10 mg, 0.03 mmol) in dry DCM, TEA (0.005 mL, 0.04 mmol) and, at 0 °C, acryloyl chloride (0.003 mL, 0.03 mmol) were added. The reaction was stirred at RT for 10 min, and after this time, the reaction was quenched with distilled water and extracted with DCM (3 × 20 mL). The organic extracts were collected, washed with brine, dried with sodium sulfate, and concentrated under reduced pressure to obtain **7d**.

Mp 190–193 °C, recrystallization solvent: acetonitrile; yield: 86.1%; ^1^H NMR (DMSO, 400 MHz, δ; ppm): 5.79 (dd, 1H, −CH=C*H*_*2*_), 5.99 (s, 2H, −CH_2_−), 6.31 (dd, 1H, −CH=C*H*_*2*_), 6.5 (q, 1H, −C*H*=CH_2_), 7.33–7.36 (m, 5H, aromatic protons), 7.65 (d, 2H, *J* = 8.8, aromatic protons), 7.83 (d, 2H, *J* = 8.4 Hz, aromatic protons), 8.83 (s, 1H, pyrimidine protons), 10.36 (s, 1H, −NH).

#### **7a**, MC4559, 1-(4-(3-Benzyl-3*H*-[1,2,3]triazolo[4,5-*d*]pyrimidin-7-yl)piperazin-1-yl)prop-2-en-1-one

Mp 140–143 °C, recrystallization solvent: toluene; yield: 33.3%; ^1^H NMR (DMSO, 400 MHz, δ; ppm): 3.80 (m, 4H, piperazine protons), 4.05 (s, 2H, piperazine protons), 4.60 (s, 2H, piperazine protons), 5.77 (dd, 1H, −CH=C*H*_*2*_), 5.81 (s, 2H, −CH_2_−), 6.18 (dd, 1H, −CH=C*H*_*2*_), 6.88 (q, 1H, −C*H*=CH_2_), 7.29–7.38 (m, 5H, aromatic protons), 8.45 (s, 1H, pyrimidine protons).

#### **7c**, MC4571, 1-(4-((3-Benzyl-3*H*-[1,2,3]triazolo[4,5-*d*]pyrimidin-7-yl)amino)piperidin-1-yl)prop-2-en-1-one

Mp 72–73 °C, recrystallization solvent: cyclohexane/toluene; yield: 49.5%; ^1^H NMR (DMSO, 400 MHz, δ; ppm): 1.55–1.58 (m, 2H, piperidine protons), 1.93–1.99 (m, 2H, piperidine protons), 2.78–2.84 (m, 1H, piperidine protons), 3.30–3.33 (m, 1H, piperidine protons), 3.36 (s, 2H, piperidine protons), 4.11–4.14 (d, 1H, *J* = 12.8, piperidine protons), 5.67–5.69 (dd, 1H, −CH=C*H*_*2*_), 5.68 (s, 2H, −CH_2_−), 6.12–6.14 (dd, 1H, −CH=C*H*_*2*_), 6.80–6.86 (q, 1H, −C*H*=CH_2_−), 7.29–7.44 (m, 5H, aromatic protons), 8.42 (s, 1H, pyrimidine proton), 8.97 (d, 1H, −NH).

### General Procedure for the Synthesis of the **6** and **12** Derivatives

#### Example: Synthesis of 3-Benzyl-*N*-(piperidin-4-yl)-3*H*-[1,2,3]triazolo[4,5-*d*]pyrimidin-7-amino Hydrochloride (**12**)

To a solution of *tert*-butyl 4-((3-benzyl-3*H*-[1,2,3]triazolo[4,5-*d*]pyrimidin-7-yl)amino)piperidine-1-carboxylate (**11**) (43 mg, 0.01 mmol) in dry THF, 4 N HCl in 1,4-dioxane was added (0.13 mL, 1.1 mmol) at 0 °C. The reaction was stirred at RT for 3 h, and after this time, the reaction was quenched with diethyl ether. The pure white precipitate of **12** was filtered off.

Mp >250 °C, recrystallization solvent: methanol; yield: 77.9%; ^1^H NMR (DMSO, 400 MHz, δ; ppm): 1.84–1.92 (m, 2H, piperidine protons), 2.07 (d, 2H, *J* = 12 Hz, piperidine protons), 3.01–3.09 (q, 2H, piperidine protons), 3.35 (d, 2H, *J* = 12 Hz, piperidine protons), 4.46–4.48 (m, 1H, piperidine protons), 5.79 (s, 2H, *J* = 6.8 Hz, −CH_2_−), 7.22–7.38 (m, 5H, aromatic protons), 8.43 (s, 1H, pyrimidine proton), 8.67 (d, 1H, *J* = 6.8 Hz, −NH−), 8.93–9.06 (m, 1H, *H*Cl), 9.19 (d, 1H, *J* = 7.2, −NH−).

#### **6**, MC4596, 3-Benzyl-7-(piperazin-1-yl)-3*H*-[1,2,3]triazolo[4,5-*d*]pyrimidine Hydrochloride

Mp 151–152 °C, recrystallization solvent: toluene; yield: 75.5%; ^1^H NMR (DMSO, 400 MHz, δ; ppm): 3.31 (s, 4H, piperazine protons), 4.26 (s, 2H, piperazine protons), 4.78 (s, 2H, piperazine protons), 5.83 (s, 2H, −CH_2_−), 7.29–7.48 (m, 5H, aromatic protons), 8.50 (s, 1H, pyrimidine proton), 9.47 (s, 2H, N*H·H*Cl).

### Synthesis of 1-(4-(3-Benzyl-3*H*-[1,2,3]triazolo[4,5-*d*]pyrimidin-7-yl)piperazin-1-yl)etan-1-one (**7b**)

To a solution of 3-benzyl-7-(piperazin-1-yl)-3*H*-[1,2,3]triazolo[4,5-*d*]pyrimidine hydrochloride (**6**) (27 mg, 0.08 mmol) in dry DCM, TEA (0.03 mL, 0.19 mmol) and, at 0 °C, acetyl chloride (0.01 mL, 0.11 mmol) were added. The reaction was stirred at RT for 30 min. After this time, the reaction was quenched with distilled water and extracted with DCM (3 × 20 mL). The organic extracts were collected, washed with brine, dried with sodium sulfate, and concentrated under reduced pressure. The obtained solid **7b** was triturated with diethyl ether and, after 30 min, filtered off.

Mp 148–150 °C, recrystallization solvent: toluene; yield: 85.6%; ^1^H NMR (DMSO, 400 MHz, δ; ppm): 2.08 (s, 3H, −CH_3_), 3.66 (d, 4H, *J* = 18.4 Hz, piperazine protons), 4.03 (d, 2H, *J* = 24.4 Hz, piperazine protons), 4.61 (d, 2H, *J* = 24.4 Hz, piperazine protons), 5.81 (s, 2H, −CH_2_−), 7.33–7.34 (m, 5H, aromatic protons), 8.44 (s, 1H, pyrimidine proton).

### Synthesis of 2-(4-(Bromomethyl)phenyl)-2-methyl-1,3-dioxolane (**14**)

To a solution of the commercially available 1-(4-(bromomethyl)phenyl)ethanone (1.50 g, 703.9 mmol) in dry benzene, *p*-toluenesulfonic acid (20.6 mg, 0.12 mmol) and ethylene glycol were added (0.79 mL, 14.08 mmol), using a Dean–Stark apparatus. The reaction was stirred at 120 °C overnight. After this time, the reaction was quenched with sodium bicarbonate and extracted with ethyl acetate (3 × 20 mL). The organic extracts were collected, washed with brine, dried with sodium sulfate, and concentrated under reduced pressure, giving **14** as a pure white solid.

Mp 148–150 °C, recrystallization solvent: toluene; yield: 96.7%; ^1^H NMR (DMSO, 400 MHz, δ; ppm): 1.66 (s, 3H, −CH_3_), 3.80 (t, 2H, *J* = 2.4 Hz, ketal protons), 4.06 (t, 2H, *J* = 2.4 Hz, ketal protons), 4.52 (s, 2H, −CH_2_−), 7.39 (d, 2H, *J* = 8.4 Hz, aromatic protons), 7.48 (d, 2H, *J* = 8.4 Hz, aromatic protons).

### General Procedure for the Synthesis of the **18** and **26** Derivatives

#### Example: Synthesis of 1-(4-((7-Hydroxy-3*H*-[1,2,3]triazole[4,5-*d*]pyrimidin-3-yl)methyl)phenyl)ethan-1-one (**18**)

To a solution of 3-(4-(2-methyl-1,3-dioxolan-2-yl)benzyl)-3*H*-[1,2,3]triazolo[4,5-*d*]pyrimidin-7-ol (**17**) (1.54 g, 4.91 mol) in EtOH, 2 N HCl (36.8 mL, 73.63 mol), at 0 °C, was added. The reaction was stirred at RT for 2 h. After this time, EtOH was evaporated, and distilled water was added. The pure white solid was filtered off to obtain **18**.

Mp 249–250 °C, recrystallization solvent: methanol; yield: 72.2%; ^1^H NMR (DMSO, 400 MHz, δ; ppm): 2.56 (s, 3H, −COCH_3_−), 5.86 (s, 2H, −CH_2_−), 7.44 (d, 2H, *J* = 8.4 Hz, aromatic protons), 7.94 (d, 2H, *J* = 8.4 Hz, aromatic protons), 8.27 (s, 1H, pyrimidine proton), 12.68 (s, 1H, −OH).

### Synthesis of 4-((7-Hydroxy-3*H*-[1,2,3]triazole[4,5-*d*]pyrimidin-3-yl)methyl)benzoic Acid (**19**)

To a solution of 1-(4-((7-hydroxy-3*H*-[1,2,3]triazole[4,5-*d*]pyrimidin-3-yl)methyl)phenyl)ethan-1-one (**18**) (200 mg, 0.76 mmol) in glacial acetic acid, bromine (0.23 mL, 4.56 mmol) was added. The reaction was stirred at 50 °C for 4 h. After this time, acetic acid was evaporated, and the reaction was quenched with 2 N NaOH (3.04 mL, 6.07 mmol) dropwise, at 0 °C, until basic pH was reached. The reaction was stirred at RT for 1 h. Afterward, 2 N HCl was added dropwise at 0 °C, until acidic pH was reached. The formed precipitate was filtered off and dried to afford the pure white compound **19**.

Mp >250 °C, recrystallization solvent: methanol; yield: 45.0%; ^1^H NMR (DMSO, 400 MHz, δ; ppm): 5.86 (s, 2H, −CH_2_−), 7.41 (d, 2H, *J* = 8 Hz, aromatic protons), 7.92 (d, 2H, *J* = 8 Hz, aromatic protons), 8.27 (s, 1H, pyrimidine proton), 12.75 (s, 1H, −COOH), 12.99 (s, 1H, −OH).

### General Procedure for the Synthesis of the **20a** and **20b** Derivatives

#### Example: Synthesis of (4-((7-Hydroxy-3*H*-[1,2,3]triazole[4,5-*d*]pyrimidin-3-yl)methyl)-*N*-(prop-2-in-1-yl)benzamide (**20b**)

To a solution of 4-((7-hydroxy-3*H*-[1,2,3]triazole[4,5-*d*]pyrimidin-3-yl)methyl)benzoic acid (**19**) (110 mg, 0.41 mmol) in dry DMF, TEA (0.283 mL, 2.03 mmol) and benzotriazol-1-yloxytripyrrolidinophosphonium hexafluorophosphate (PyBOP) (253.25 mg, 0.49 mmol) were added, under a nitrogen atmosphere. The reaction was stirred at RT for 45 min to afford the activation of the acid. Afterward, at 0 °C, propargylamine (0.11 mL, 1.62 mmol) was added, and then, the reaction was stirred at RT overnight. The reaction was quenched with brine. Afterward, 2 N HCl was added dropwise, at 0 °C, until acidic pH was reached and extracted with ethyl acetate (3 × 20 mL). The organic extracts were collected, washed with brine, dried with sodium sulfate, and concentrated under reduced pressure. The resulting reaction mixture was purified by column chromatography (SiO_2_, eluted with chloroform/methanol 12:1) to provide pure compound **20b**.

Mp 220–223 °C, recrystallization solvent: acetonitrile/methanol; yield: 40.0%; ^1^H NMR (DMSO, 400 MHz, δ; ppm): 3.12 (t, 1H, *J* = 2.4 Hz, propargylic protons), 4.04 (q, 2H, −C*H*_*2*_–C≡CH), 5.83 (s, 2H, −CH_2_−), 7.41 (d, 2H, *J* = 5.6 Hz, aromatic protons), 7.83 (d, 2H, *J* = 5.6 Hz, aromatic protons), 8.27 (s, 1H, pyrimidinic proton), 8.93 (t, 1H, *J* = 5.6 Hz, −CONH−), 12.64–12.75 (s, 1H, −OH).

### General Procedure for the Synthesis of the Final Compounds **8a**, **8b**, and **9a**–**f**

#### Example: Synthesis of (4-((7-Benzo[*d*]oxazol-2-yl-thio)-3*H*-[1,2,3]triazole[4,5-*d*]pyrimidin-3-yl)methyl)-*N*-(prop-2-in-1-yl)benzamide, **8b**, MC4767

To a solution of 4-((7-chloro-3*H*-[1,2,3]triazole[4,5-*d*]pyrimidin-3-yl)methyl)-*N*-(prop-2-in-1-yl)benzamide (**21b**) (52 mg, 0.16 mmol) in dry EtOH, TEA (0.02 mL, 0.16 mmol) and, at 0 °C, 2-mercaptobenxozaole (24 mg, 0.16 mmol) were added. The reaction was stirred at RT for 30 min. The reaction was quenched with distilled water and extracted with DCM (3 × 20 mL). The organic extracts were collected, washed with brine, dried with sodium sulfate, and concentrated under reduced pressure. The resulting reaction mixture was purified by column chromatography (SiO_2_, eluted with ethyl acetate/chloroform 1:6) to provide pure compound **8b**.

Mp 136–139 °C, recrystallization solvent: toluene; yield: 30.0%; ^1^H NMR (CDCl_3_, 400 MHz, δ; ppm): 2.21 (s, 1H, propargylic protons), 4.17 (q, 2H, −C*H*_*2*_–C≡CH), 5.80 (s, 2H, −CH_2_−), 7.35–7.79 (m, 8H, aromatic protons), 8.74 (s, 1H, −NH−), 8.87 (S, 1H, −CONH−).

#### **8a**, MC4768, (4-((7-Benzo[*d*]oxazol-2-yl-thio)-3*H*-[1,2,3]triazole[4,5-*d*]pyrimidin-3-yl)methyl)-*N*-(propyl)benzamide

Mp 162–165 °C, recrystallization solvent: toluene/acetonitrile; yield: 32.2%; ^1^H NMR (CDCl_3_, 400 MHz, δ; ppm): 0.90 (t, 3H, *J* = 8 Hz, −NH–CH_2_–CH_2_–C*H*_*3*_), 1.52–1.58 (m, 2H, NH–CH_2_–C*H*_*2*_–CH_3_), 3.31–3.36 (q, 2H, −NH–C*H*_*2*_–CH_2_–CH_3_), 5.80 (s, 2H, −CH_2_−), 5.96 (s, 1H, −N*H*–CH_2_–CH_2_–CH_3_), 7.35–7.39 (m, 4H, aromatic protons), 7.53 (dd, 1H, aromatic protons), 7.66 (d, 2H, *J* = 8.4 Hz, aromatic protons), 7.78 (dd, 1H, aromatic protons), 8.74 (s, 1H, pyrimidinic proton).

#### **9a**, MC4762, *N*-(4-((7-(Benzo[*d*]oxazol-2-ylthio)-3*H*-[1,2,3]triazolo[4,5-*d*]pyrimidin-3-yl)methyl)benzyl)-*N*-methylprop-2-yn-1-amine

Mp 102–105 °C, recrystallization solvent: cyclohexane/toluene; yield: 32.0%; ^1^H NMR (CDCl_3_, 400 MHz, δ; ppm): 2.29 (s, 1H, −CH_2_–C≡C*H*), 2.34 (s, 3H, −N–C*H*_*3*_), 3.30 (s, 2H, −C*H*_*2*_–C≡CH), 3.58 (s, 2H, −C*H*_*2*_–N–CH_3_), 5.84 (s, 2H, −N–C*H*_*2*_−), 7.35 (d, 2H, aromatic protons, *J* = 8 Hz), 7.42 (d, 2H, aromatic protons, *J* = 8 Hz), 7.46–7.51 (m, 2H, aromatic protons), 7.62 (d, 1H, aromatic protons, *J* = 7.2 Hz), 7.87 (d, 1H, aromatic protons, *J* = 8 Hz), 8.84 (s, 1H, pyrimidinic protons).

#### **9b**, MC4998, *N*-(4-((7-(Benzo[*d*]oxazol-2-ylthio)-3*H*-[1,2,3]triazolo[4,5-*d*]pyrimidin-3-yl)methyl)benzyl)-*N*-methylpropan-1-amine

Mp 154–155 °C, recrystallization solvent: toluene/acetonitrile; yield: 18.5%; ^1^H NMR (CDCl_3_, 400 MHz, δ; ppm): 1.68–1.76 (m, 2H, −CH_2_–C*H*_*2*_–CH_3_), 3.31 (s, 3H, −CH_3_), 3.76 (t, 3H, *J* = 7.6 Hz, −CH_2_–C*H*_*3*_), 4.21 (t, 2H, *J* = 7.6 Hz, −C*H*_*2*_–CH_2_–CH_3_), 4.44 (s, 2H, −C*H*_*2*_), 5.66 (s, 2H, −C*H*_*2*_−), 7.34 (m, 6H, aromatic protons), 7.53 (dd, 2H, aromatic protons), 8.35 (s, 1H, pyrimidine proton).

#### **9c**, MC4999, *N*-(4-((7-(Benzo[*d*]oxazol-2-ylthio)-3*H*-[1,2,3]triazolo[4,5-*d*]pyrimidin-3-yl)methyl)benzyl)-*N*-methyl-1-phenylmethanamine

Mp liquid, yield: 14.8%; ^1^H NMR (CDCl_3_, 400 MHz, δ; ppm): 1.98 (s, 2H, −*CH*_*2*_), 3.44 (s, 2H, −*CH*_*2*_), 3.46 (s, 3H, −*CH*_*3*_), 5.78 (s, 2H, −*CH*_*2*_), 7.33 (m, 8H, aromatic protons), 7.52 (t, 3H, *J* = 6 Hz, aromatic protons), 7.78 (m, 2H, aromatic protons), 8.73 (s, 1H, pyrimidine proton).

#### **9d**, MC5018, 2-((3-(4-(Piperidin-1-ylmethyl)benzyl)-3*H*-[1,2,3]triazolo[4,5-*d*]pyrimidin-7-yl)thio)benzo[*d*]oxazole

Mp 130–133 °C, recrystallization solvent: toluene; yield: 23.5%; ^1^H NMR (CDCl_3_, 400 MHz, δ; ppm): 1.20 (s, 2H, −CH_2_−), 1.36–1.47 (m, 2H, piperidine protons), 1.65–1.75 (m, 4H, piperidine protons), 2.49–2.55 (m, 2H, piperidine protons), 3.59–3.67 (m, 2H, piperidine protons), 5.75 (s, 2H, −C*H*_*2*_−), 7.35–7.42 (m, 6H, aromatic protons), 7.53 (d, 1H, *J* = 8.8 Hz, aromatic protons), 7.78 (d, 1H, *J* = 8.8 Hz, aromatic protons), 8.75 (s, 1H, pyrimidine protons).

#### **9e**, MC4982, 2-((3-(4-(Morpholinomethyl)benzyl)-3*H*-[1,2,3]triazolo[4,5-*d*]pyrimidin-7-yl)thio)benzo[*d*]oxazole

Mp 89–90 °C, recrystallization solvent: cyclohexane/toluene; yield: 89.9%; ^1^H NMR (CDCl_3_, 400 MHz, δ; ppm): 2.32 (s, 4H, morpholine protons), 3.38 (s, 2H, −C*H*_*2*_*–*) 3.61 (s, 4H, morpholine protons), 5.74 (s, 2H, −C*H*_*2*_−), 7.23 (d, 2H, *J* = 7.6 Hz, aromatic protons), 7.36 (d, 2H, *J* = 7.6 Hz, aromatic protons), 7.34–7.39 (m, 2H, aromatic protons), 7.42 (dd, 1H, aromatic protons), 7.78 (dd, 1H, aromatic protons), 8.74 (s, 1H, pyrimidine protons).

#### **9f**, MC5000, 2-((3-(4-((2,2-Difluoro-7-azaspiro[3.5]nonan-7-yl)methyl)benzyl)-3*H*-[1,2,3]triazolo[4,5-*d*]pyrimidin-7-yl)thio)benzo[*d*]oxazole

Mp liquid, yield: 11.0%; ^1^H NMR (CDCl_3_, 400 MHz, δ; ppm): 1.23 (s, 4H, cyclobutane protons), 2.23–2.31 (m, 8H, piperidine protons), 5.23 (s, 2H, −CH_2_−), 5.75 (s, 2H, −CH_2_−), 7.35–7.42 (m, 6H, aromatic protons), 7.52 (dd, 1H, aromatic protons), 7.79 (dd, 1H, aromatic proton), 8.74 (s, 1H, pyrimidine proton).

#### Synthesis of 1-(Bromomethyl)-4-(diethoxymethyl)benzene (**22**)

To a solution of the commercially available 4-(bromomethyl)benzaldehyde (160 mg, 0.80 mmol) in dry EtOH, triethyl orthoformate (1.4 mL, 8.19 mmol) and Dowex 50W X8 (152.4 mg) were added. The reaction was stirred at RT for 3 h. After this time, half of the EtOH was evaporated and 2 N sodium carbonate (152.4 mg) was added and stirred at RT for 20 min. Afterward, the solution was filtered to remove sodium carbonate and Dowex 50W X8, obtaining compound **22** as a yellow oil.

Mp liquid; yield: 95.5%; ^1^H NMR (DMSO, 400 MHz, δ; ppm): 1.11–1.17 (m, 6H, −CH–(O–CH_2_–C*H*_*3*_)_2_), 3.42–3.57 (m, 4H, −CH–(O–C*H*_*2*_–CH_3_)_2_), 5.42 (s, 2H, −CH_2_−), 5.45 (s, 1H, −C*H*–(O–CH_2_–CH_3_)_2_), 6.38 (s, 2H, −NH_2_), 7.21 (d, 2H, *J* = 8 Hz, aromatic protons), 7.38 (d, 2H, *J* = 8 Hz, aromatic protons), 6.38 (s, 2H, −NH_2_).

### General Procedure for the Synthesis of **27a**–**f** Derivatives

#### Example: Synthesis of 3-(4-((Methyl(prop-2-yn-1-yl)amino)methyl)benzyl)-3*H*-[1,2,3]triazolo[4,5-*d*]pyrimidin-7-ol (**27a**)

To a solution of 4-((7-hydroxy-3*H*-[1,2,3]triazole[4,5-*d*]pyrimid-3-yl)methyl)benzaldehyde (**26**) (20 mg, 0.08 mmol) in dry DCM, *N*-methyl-propargylamine (0.007 mL, 0.08 mmol) and sodium triacetoxyborohydride (21.6 mg, 0.18 mmol) were added, under a nitrogen atmosphere. The reaction was stirred at RT for 4 h. Afterward, the reaction was quenched with distilled water and extracted with DCM (3 × 20 mL). The organic extracts were collected, washed with brine, dried with sodium sulfate, and concentrated under reduced pressure. The resulting reaction mixture was purified by column chromatography (SiO_2_, eluted with ethyl chloroform/methanol 15:1) to provide pure white compound **27a**.

Mp 185–186 °C; recrystallization solvent : acetonitrile; yield = 66.2%; ^1^H NMR (DMSO, 400 MHz, δ; ppm): 2.23 (s, 3H, −N–CH_3_), 3.24 (s, 1H, −C≡C*H*), 3.30 (s, 2H, −C*H*_*2*_–C≡CH), 3.53 (s, 2H, −C*H*_*2*_–N–CH_3_), (s, 2H, −C*H*_*2*_–C≡CH), 5.80 (s, 2H, −N–C*H*_*2*_–benzyl), 7.32–7.37 (m, 4H, aromatic protons), 8.33 (s, 1H, pyrimidinic proton), 12.76 (s, 1H, −OH).

### Purity Control by HPLC of Compounds **4**, **7d**, and **9a**

The purity of selected compounds was analyzed by HPLC. The HPLC system consisted of a Dionex UltiMate 3000 UHPLC (Thermo Fisher) system equipped with an automatic injector and a column heater and coupled with a diode array detector DAD-3000 (Thermo Fisher). The analytical controls were performed on a Hypersil GOLD C18 Selectivity 5 μm (4.6 × 250 mm) HPLC column (Thermo Fisher) in gradient elution. Eluents: (A) H_2_O + 0.1% TFA; (B) CH_3_CN + 0.1% TFA. A 20 min linear gradient elution from 10 to 90% solvent B was followed by 5 min at 100% B. The flow rate was 1.0 mL/min, and the column was kept at a constant temperature of 30 °C. Samples were dissolved in solvent A at a concentration of 0.25 mg/mL, and the injection volume was 3 μL.

By analyzing the HPLC traces at both 254 and 280 nm, a chemical purity >95% was recorded for both wavelengths. Chromatographic traces acquired at 254 and 280 nm are reported in the Supporting Information.

### Biochemical and Cellular Reagents

Reagents FAD disodium salt hydrate (FAD-Na_2_), β-nicotinamide adenine dinucleotide 2′-phosphate reduced tetrasodium salt (NADPH), Roswell Park Memorial Institute (RPMI) 1640 medium with l-glutamine and sodium bicarbonate, phosphate-buffered saline (PBS), lithium dodecyl sulfate (LiDS), CaCl_2_, sodium chloride, HEPES buffer, sodium dihydrogen phosphate (NaH_2_PO_4_·H_2_O), phenylmethylsulfonyl fluoride, glycerol, pepstatin, leupeptin, SigmaFast protease inhibitor cocktail tablets EDTA-free, cytochrome c from equine heart, hemin, superoxide dismutase, Ampliflu Red (Amplex Red) and horseradish peroxidase, Triton X-100 reduced, phorbol myristate acetate (PMA), VAS2870 and VAS3947, kynuramine, and benzylamine were purchase from Sigma-Aldrich. MCLA was purchased from MedChemExpress. Cholesteryl hemisuccinate (CHS), dodecyl-β-d-maltopyranoside (DDM), β-octylglucoside (OG), and *n*-dodecyl-phosphocholine (Fos-Choline-12) were purchased from Anatrace. Fetal bovine serum (FBS), FreeStyle and DMEM (cat. no. 31966047) media, penicillin G, and streptomycin were purchased from Invitrogen (Carlsbad, CA).

### Protein Expression

Human MAOA and MAOB were overexpressed in *Pichia pastoris* as described.^58^ The N-terminally histidine-tagged wild-type dehydrogenase domain of *cs*NOX5 (residues 413–693) was expressed in *E. coli* as described.^10^ Human p67^phox^, p47^phox^, and Rac1 Q61L mutants were expressed in *E. coli* as described.^42^

The complementary DNAs (cDNAs) encoding for the human NOX1 (isoform 1), human NOX5 (isoform v/2), and human p22^phox^ were obtained from GenScript and cloned into a pEG BacMam vector (kindly gifted by E. Gouaux, Oregon Health and Science University, Portland). The cDNAs encoding for the human NOX4 (isoform 1) and human p22^phox^ were obtained from GeneWiz and cloned into pTT22 and pYD7 vectors, respectively (vectors obtained from Y. Durocher, NRC-BRI, Montreal, Canada.) All these constructs were expressed as reported^42^ with some variations. Briefly, HEK293 F cells were cultured in a FreeStyle medium and seeded at 5 × 10^5^ cells/mL, at 37 °C and 5% CO_2_, 24 h before transfection. NOX1-p22^phox^, NOX5, and NOX4-p22^phox^ were transiently transfected using polyethylenimine (NOX1 75% and p22 25%; NOX4 90% and p22 10%; NOX5 100%). After 48 h, cells were collected by centrifugation (1200*g*, 5 min, 4 °C). The cell pellets were frozen in liquid nitrogen and stored at −80 °C.

NOX2 was expressed as native protein in X-CGD PLB-985 cells transduced with the RD114 pseudotyped MFGS-NOX2 vector (PLB-985 cells from now on), a kind gift from H. Malech (National Institutes of Health), as described.^40^ In short, cells were cultured in RPMI-1640 with 10% FBS at 37 °C and 5% CO_2_. After cell collection by centrifugation at 1200*g* for 10 min, the pellet was frozen in liquid nitrogen and stored at −80 °C.

### Protein Purification

#### MAOA and MAOB

Human MAOB purified samples were obtained via ion exchange chromatography following established protocols.^58^ The flavin absorbance at 456 nm (ε_456_ = 12,000 M^–1^ cm^–1^) was measured with a NanoDrop ND-100 to assess the protein concentration. The purified protein sample for enzymatic assays was stored in a 50 mM potassium phosphate buffer (pH 7.5), 20% (w/v) glycerol, and 0.8% (w/v) β-octylglucoside. Human MAOA detergent-purified samples were obtained via immobilized metal affinity chromatography as previously reported.^58^ The protein concentration was assessed on the cofactor spectrum as described above. The purified protein sample for enzymatic assays was desalted with a 5 mL HiTrap desalting column (Cytiva) and stored in a 50 mM sodium phosphate buffer, pH 7.8, 300 mM sodium chloride, 20% (w/v) glycerol, and 0.05% (w/v) Fos-Choline-12.

#### *C. stagnale* NOX5 Dehydrogenase Domain

The N-terminally histidine-tagged wild-type protein (residues 413–693) was purified as described^10^ with some optimizations. Briefly, the cell pellet was resuspended in a lysis buffer (50 mM HEPES pH 7.5, 5% glycerol, and 300 mM NaCl) with protease inhibitors, disrupted by sonication, and centrifuged (56,000*g* for 45 min). The supernatant was purified on a Ni^2+^ column through immobilized metal affinity chromatography. The sample was run on a Superdex 75 column equilibrated in a lysis buffer.

#### Human NOX1 and NOX2 Cytosolic Partners

The full-length human p67^phox^, p47^phox^, and the constitutively active mutant Rac1 Q61L were purified as described.^40^ Briefly, the cell pellet was lysed in a buffer (20 mM sodium phosphate, 0.5 mM NaCl, and 20 mM imidazole, pH 7.4) with a protease inhibitor and 1% Triton X-100. The lysate was sonicated and centrifuged (34,000*g* for 30 min at 4 °C). Nickel-Sepharose beads were used for in-batch immobilized metal affinity chromatography, followed by size-exclusion chromatography on Superdex 200 and 75 columns (Cytiva) using FPLC. Purified proteins were supplemented with 10% glycerol and stored at −80 °C.

#### Human NOX Membrane Preparations

Membrane isolation of NOX-expressing human cells was performed as previously reported.^40−42^ Briefly, transfected HEK293 F cells or PLB-985 cell pellets were resuspended in a sonication buffer containing 10 mM HEPES at pH 7.4, 10 mM NaCl, and 100 mM KCl. The lysate was centrifuged (500*g* for 5 min at 4 °C), and the supernatant was collected. The cell pellet was resuspended in a sonication buffer, sonicated again, and then centrifuged (500*g* for 5 min at 4 °C), and the supernatant was collected. Both supernatants were ultracentrifuged (200,000*g* for 30 min at 4 °C) (Optima MAX-XP ultracentrifuge, Beckman Coulter).

#### CS9 Antibody Expression and Purification

The hybridoma cell line was a kind gift from A. Jesaitis (Montana State University, USA). The cell line was cultured for 7 days, and the antibody was precipitated with ammonium sulfate and purified with a HiTrap Protein G HP column (Cytiva).^40^

#### Immunoaffinity Purification of Human NOX2-p22^phox^

NOX2-p22^phox^ heterodimer purification was performed as described.^40^ In short, PLB-985 isolated membranes were supplemented with 1% (v/v) DDM and 0.2% (v/v) CHS for protein isolation. After centrifugation at 100,000*g,* the solubilized fraction was applied to a Protein G Sepharose 4 Fast Flow Resin (Cytiva), prepacked with the p22^phox^-specific mAb CS9, and equilibrated with a sonication buffer (10 mM HEPES pH 7.4, 10 mM NaCl, and 100 mM KCl) supplemented with 0.026% DDM (v/v) and CHS 0.0052% (v/v) and eluted with 200 μM p22^phox^ epitope peptide Ac-AEARKKPSEEEAA-NH2 (GenScript). Affinity chromatography was followed by gel filtration on a Superdex 200 5/150 increase 5/150 (Cytiva).

### Biochemical Assays

IC_50_ values were calculated as the concentrations at the point halfway between the bottom (no inhibition) and top (highest inhibition) plateaus of the inhibition curves.

#### Purified MAOs

IC_50_ values on human MAOA and human MAOB were determined using the Amplex Red/horseradish peroxidase coupled assay. Briefly, 70 nM MAOA or 10 nM MAOB was mixed in an assay buffer (43.3 mM HEPES pH 7.5 and 0.25% v/v Triton X-100-reduced) with 1 mM substrates, kynuramine or benzylamine, respectively, together with 0.02 U mL^–1^ horseradish peroxidase and 12.5 μM Amplex Red, and incubated for 30 min with increasing concentrations of the inhibitor. Measurements were performed using a ClarioStar plate reader (excitation 572 nm and emission 583 nm) (BMG Labtech). IC_50_ values were obtained by fitting the percentage of inhibition versus log(inhibitor concentration) using GraphPad software 7.0. The covalent modification of the flavin upon the reaction with the propargylamine-containing inhibitors was monitored spectrophotometrically using a diode array UV/visible spectrophotometer (Agilent Technologies). The reaction was started by adding a 5-fold molar excess of compound **9a** to 20 μM protein in a volume of 100 μL of a purification buffer. The cofactor covalent modification was monitored over time following the shift of the enzyme-bound flavin (peaks at 370 and 456 nm) to 410 nm (ε_410_ = 23,500 M^–1^ cm^–1^) (Figure S10).

#### *C. stagnale* NOX5 Dehydrogenase Domain

The NADPH depletion assay for IC_50_ determinations was performed as described^10^ with modifications for covalent inhibitors, which require longer incubation times. Briefly, the catalytic activity was measured by monitoring the absorbance at 340 nm with a Cary 100 UV–vis spectrophotometer (Varian). A 1 μM portion of purified protein was added in a final volume of 130 μL of 50 mM HEPES pH 7.5 and supplemented with 300 μM FAD. Inhibitors were added at varying concentrations and incubated for 1 h at 25 °C.

#### Human NOX1, NOX4, and NOX5 Isolated Membranes

Activity assays for IC_50_ determinations were performed as previously described^40,42^ with modifications for covalent inhibitors. Briefly, 25 μg (NOX5) and 100 μg (NOX1-p22^phox^ and NOX4-p22^phox^) of isolated membranes were added to a reaction mix containing PBS, 0.02 U/mL horseradish peroxidase, 12.5 μM Amplex Red, 40 μM FAD, 1.5 μM cytosolic proteins (p67^phox^, p47^phox^, and Rac1 Q61L for NOX1), and 1 mM CaCl_2_ for NOX5 in a 100 μL volume. The estimated final protein concentrations based on the heme’s absorbance (ε_414 nm_ = 131,000 M^–1^ cm^–1^) were 10–20 nM. Compounds were incubated for 1 h at 25 °C at varying concentrations, and reactions were initiated with 40 μM NADPH. Fluorescence was measured using a ClarioStar plate reader (excitation 572 nm/emission 583 nm).

#### Human NOX2-p22^phox^ Isolated Membranes

The strong activity of NOX2 enabled the use of the peroxide-independent MCLA and cytochrome c assays that probe superoxide formation. IC_50_ determinations were performed as described, with the same aforementioned modifications for covalent inhibition. Briefly, 20 μg of NOX2-p22^phox^ membranes was added to a reaction mix with a 65 mM sodium phosphate buffer (pH 7.0), 0.5 μM FAD, 130 μM LiDS, 160 nM p67^phox^, p47^phox^, Rac1 Q61L, and 1 μM MCLA or 200 μM bovine cytochrome c. The estimated final protein concentration based on the heme’s absorbance was 60 nM. Compounds were incubated for 1 h at 25 °C, at varying concentrations. The reaction started by adding 240 μM NADPH, and measurements were taken by using a GloMax plate reader (Promega; MCLA) or a Cary 100 UV–vis spectrophotometer (Varian; cytochrome c).

#### Purified Human NOX2-p22^phox^

The NADPH depletion assay on human NOX2 for IC_50_ determinations was performed as described^40,42^ with variations for covalent inhibition. Briefly, the catalytic activity was measured by monitoring the absorbance at 340 nm with a Cary 100 UV–vis spectrophotometer (Varian). A 200 nM purified NOX2-p22^phox^ heterodimer was added to a 200 μL reaction mix containing a 65 mM sodium phosphate buffer (pH 7.0), 50 μM FAD, 130 μM LiDS, and 1 μM recombinant p67^phox^, p47^phox^, and Rac1 Q61L. Inhibitors were added at varying concentrations (7 nM–200 μM) and incubated for 1 h at 25 °C. The reaction was started by the addition of 240 μM NADPH.

#### *In Cellulo* IC_50_ Determination on Human NOX2

Measurement of human NOX2 activity in cells was performed by a cytochrome c (200 μM) reduction assay. Superoxide production of intact PLB-985 cells (3 × 10^5^ cells) was measured after PMA stimulation (5 μM) as reported.^40,42^ The compounds (0.04–40 μM) were added to the prestimulated cells and incubated for 1 h at 37 °C and 5% CO_2_, 120 rpm (New Brunswick S41i incubator shaker, Eppendorf). The absorbance at 550 nm was followed at 37 °C over a time course of 20 min in a ClarioStar plate reader (BMG Labtech).

#### Control Assays

Control assays were conducted to evaluate potential interference of the compounds (ROS-scavenging and off-target effects, as shown in refs (42 and 43)) with Amplex Red, MCLA, NADPH consumption, and cytochrome c reduction assays, using inhibitors at a concentration of 10 μM. No or minimal interference was detected. Membranes from nontransfected cells (NOX4) or from nonactivated membranes (NOX1, NOX2, and NOX5) were used as a control for inhibitor evaluation and determination of the background signal (Figure S16).

### Biophysical Methods for Covalent Bond Determination

#### X-ray Crystallography on Human MAOB

Previously published protocols were applied to determine the MAOB/**9a** complex structure.^49^ Briefly, about 50 μM protein was incubated with 200 μM in a purification buffer at 4 °C for about 30 min. The flavin-inhibitor adduct formation was monitored spectrophotometrically with a NanoDrop ND-100, following the shift to 410 nm of the visible spectrum. Inhibited MAOB was gel-filtered in a 25 mM potassium phosphate buffer pH 7.2 supplemented with 8.5 mM Zwittergent 3-12, concentrated to about 1.5 mg mL^–1^, and crystallized by the sitting-drop method. Crystals were obtained at 4 °C in about 1 week, cryo-protected into a mother liquor supplemented with 18% (v/v) glycerol, and flash-cooled in liquid nitrogen. X-ray diffraction data were collected at the ID30A-1/MASSIF-1 beamline (ESRF, Grenoble, France) at 100 K. XDS^59^ and CCP4^60^ were used for data processing and scaling (Table S3). Coot^61^ was used for electron density map inspection and model building. REFMAC5^62^ was used for refinement. Structural data were deposited into the PDB (entry 9FJT).

#### Intact Protein Mass Spectrometry on *C. stagnale* NOX5 Dehydrogenase

Approximately 1 mg mL^–1^ protein was incubated with 300 μM FAD and 1 mM **9a** at 4 °C for 1 h. A negative control reaction was performed by adding an equal volume of DMSO. Protein was denatured by adding 1% (v/v) formic acid. Chromatographic separation and mass spectrometry analysis were carried out as previously reported.^10^ Analyses were carried out by ESI-qTOF-HRMS on an X500B qTOF system (Sciex) embedding a Twin Sprayer ESI probe and coupled with an ExcionLC system (Sciex) controlled by SciexOS software v. 3.0.0. The injection volume was 10 μL (10 μg), and chromatographic separation was carried out with a bioZen WidePore C4 column (a 100 mm length, a 2.1 mm diameter, and a 2.6 μm particle size; Phenomenex).

### Mass Spectrometric Analysis for GSH, BSA, and Myoglobin Reactivity

Reactivity between **9a** and GSH was measured via mass spectrometry on a Q Exactive UHMR hybrid quadrupole-Orbitrap mass spectrometer (Thermo Fisher) with positive polarity. The instrument parameters used for MS spectra collection were the following: capillary voltage, 1.2 kV; scan range from 350 to 2000 *m*/*z*; HCD collision voltage, 0 V; source fragmentation, 0 V; in-source trapping, 0 V. The ion transfer optics was set as follows: injection flatapole, 5 V; inner-flatapole lens, 4 V; bent flatapole, 2 V; transfer multipole, 0 V. The resolution of the instrument was 200,000 at *m*/*z* = 400, the nitrogen pressure in the HCD cell was maintained at approximately 9.8 × 10^–11^ mbar, and the source temperature was kept at 100 °C. Solutions for flow injection analysis were constituted by dissolving each compound in LC-MS-grade MeOH.

Protein samples were buffer-exchanged into 200 mM ammonium acetate (pH 7.5) through dialysis prior to native MS analyses. These samples were directly introduced into the mass spectrometer using gold-coated capillary needles prepared in-house.^63^ Data were collected on a Q Exactive UHMR hybrid quadrupole-Orbitrap mass spectrometer (Thermo Fisher) with positive polarity. The instrument parameters used for MS spectra collection were the following: capillary voltage, 1.2 kV; scan range from 1000 to 20,000 *m*/*z*; HCD collision energy, 0–100 V; source fragmentation, 0 V; in-source trapping, 0 V. The ion transfer optics was set as follows: injection flatapole, 5 V; interflatapole lens, 4 V; bent flatapole, 2 V; transfer multipole, 0 V. The resolution of the instrument was 17,500 at *m*/*z* = 200 (transient time of 64 ms), the nitrogen pressure in the HCD cell was maintained at approximately 4 × 10^–10^ mbar, and the source temperature was kept at 100 °C. The noise level was set at 3 rather than the default value of 4.64. Calibration of the instruments was performed using 10 mg/mL solution of cesium iodide in water. Data were analyzed using Xcalibur 3.0 (Thermo Scientific), NaViA,^64^ and UniDec software packages.^65^

### Evaluation of NOX2 Inhibition upon Preactivation with the Cytosolic Partners

The distinct effects of VAS2870 and of compound **9a** on NOX2 based on the accessibility of its active site upon preactivation by the cytosolic partners were evaluated by cytochrome c reduction assay *in vitro*. More in detail, two different mixtures were prepared simultaneously: a “nonpreactivated NOX2” mixture in the absence of cytosolic partners and a “preactivated NOX2” mixture in the presence of cytosolic partners. NOX2-containing membranes (3.9 mg/mL) were added to a sodium phosphate buffer (65 mM, pH 7.0), 50 μM FAD, and 130 μM LiDS, in the presence or absence of 160 nM p67^phox^, p47^phox^, and Rac1 Q61L in a final volume of 20 μL. Both mixes were incubated with DMSO (vehicle) or 1 mM of the tested compound for 60 min at 25 °C. The mixtures were then centrifuged (20,000*g* for 10 min) at 4 °C to gently remove the excess of the inhibitor and, if present, of the activators. The supernatants were discarded to eliminate the excess of the nonbound compound, and the pellets were resuspended in the same volume of the fresh buffer. Subsequently, the resuspended pellets were loaded into a spectrophotometric cuvette at a final concentration of 0.39 mg/mL and incubated with fresh cytosolic activators (160 nM), LiDS (130 μM), and cytochrome c (200 μM) for 10 min at 25 °C. The cytochrome c assay was chosen because of its higher sensitivity that permitted the measurement of the lower activities of the partially inhibited proteins. The reaction was then initiated with 240 μM NADPH and monitored at 550 nm with a Cary 100 UV–vis spectrophotometer (Varian).

### Biological Experiments in BV2 Cells

#### Microglial Cell Line

BV2 murine microglial cells were cultured in DMEM supplemented with 10% heat-inactivated FBS, 100 IU/mL penicillin G, 100 mg/mL streptomycin, and 2.5 mg/mL amphotericin B and grown at 37 °C in 5% CO_2_ and a humidified atmosphere. Cells were preincubated with 10 μM VAS 2870, MC4762, and MAOB inhibitor rasagiline. After 30 min, LPS 100 ng/mL and IFN-γ 20 ng/mL were added to the medium for an additional 48 h.

#### ROS Detection

After treatment, BV2 cells were incubated with a CellROX Deep Red reagent (cat. no. C10422) at 5 μM and incubated for 30 min at 37 °C as indicated by the manufacturer protocol. ROS levels were measured by a spectrofluorometer, taking as 100% the median fluorescence intensity of cells treated with the vehicle.

#### Viability Assay

Cell viability was determined by the MTT assay. Briefly, MTT (dissolved in PBS with a final concentration of 0.5 mg/mL) was added to the medium culture. After 2 h of incubation, the cells were mixed with DMSO and shaken for 10 min. Finally, the absorption of the samples was read by regulating the 570 nm filter as the main wavelength and the 630 nm filter as the referenced wavelength. The blank was subtracted from all samples to obtain pure cellular absorption. Results are expressed as the percentage of cell survival, taking 100% of the cells treated with the vehicle.

#### RNA Extraction, Reverse Transcription, and Real-Time PCR

BV2 cells were lysed in a Trizol reagent for isolation of RNA. A reverse transcription reaction was performed in a thermocycler (MJMini personal thermal cycler; Biorad) using an IScript Reverse Transcription Supermix (Biorad) according to the manufacturer’s protocol, under the following conditions: incubation at 25 ° C for 5 min, reverse transcription at 42 ° C for 30 min, and inactivation at 85 ° C for 5 min. Real-time PCR (rtPCR) was carried out in an I-Cycler IQ Multicolor rtPCR detection system (Biorad) using a SsoFast EvaGreen Supermix (Biorad) according to the manufacturer’s instructions. The PCR protocol consisted of 40 cycles of denaturation at 95 ° C for 30 s and annealing/extension at 60 ° C for 30 s. For quantification analysis, the comparative threshold cycle (Ct) method was used. The Ct values from each gene were normalized to the Ct value of GAPDH in the same RNA samples. Relative quantification was performed using the 2^–ΔΔCt^ method (Schmittgen and Livak, 2008) and expressed as a fold change in arbitrary values. Primers used are as follows:

*iNOS* fw: ACATCGACCCGTCCACAGTAT; rev: CAGAGGGGTAGGCTTGTCTC.
*IL-1*β fw: GCAACTGTTCCTGAACTCAACT; rev: ATCTTTTGGGGTCCGTCAACT.
*IL-6* fw: GATGGATGCTACCAAACTGGA; rev: TCTGAAGGACTCTGGCTTTG.

## Supporting Information Available

Chemical and physical properties for intermediate compounds **11**–**19**, **20a**–**b**, **21a**–**b**, **22**–**26**, **27a**–**f**, and **28a**–**f** (Table S1); elemental analysis for compounds **2**–**6**, **7a**–**d**, **8a**–**b**, and **9a**–**f** (Table S2); copies of the ^1^H NMR spectra; HPLC trace for compounds **4**, **7d**,and **9a**; mass spectrometry data for **7c** binding to *cs*NOX5 (Figure S1); mass spectrometry analysis for GSH, BSA, and myoglobin reactivity (Figures S12–S14); IC_50_ determination against NOX1, NOX2, NOX4, and NOX5 and against purified human NOX2 (Figures S2–S6); IC_50_ determination on NOX2-overexpressing cells (Figure S7); IC_50_ determination against MAOA and MAOB (Figures S8 and S9); UV spectrum of the MAOB-flavin/**9a** complex (Figure S10); hydrogen peroxide or superoxide production by NOXs and their respective controls (Figure S11); data collection and refinement statistics for the MAOB/**9a** complex (Table S3); qRT-PCR analysis (Figure S16); biochemical inhibition raw data (PDF)
Molecular formula strings (CSV)

## Accession Codes

Crystal structure of human MAOB bound to **9a** has been deposited under PDB ID 9FJT.

## Abbreviations

Aβ: amyloid β
*cs*DH-NOX5: DH domain of NOX5 from *Cylindrospermum stagnale*
DCM: dichloromethane
DH: dehydrogenase
DMF: *N*,*N*-dimethylformamide
DMSO: dimethylsufoxide
DUOX1–2: dual oxidases 1–2
EPAC: exchange protein activated by cAMP
EtOH: ethanol
EtONa: sodium ethoxide
ERK1/2: extracellular signal-related kinases 1 and 2
FAD: flavin adenine dinucleotide
FBS: fetal bovine serum
GAPDH: glyceraldehyde-3-phosphate dehydrogenase
IFN-γ: interferon-gamma
IL-1β: interleukin-1beta
IL-6: interleukin-6
iNOS: inducible nitric oxide synthase
NADPH: nicotinamide adenine dinucleotide phosphate
GTP: guanosine triphosphate
LPS: lipopolysaccharide
M41: Molport41
MAOA and B: monoamine oxidases A and B
MeOH: methanol
mp: melting point
MTT: 3-[4,5-dimethylthiazol-2-yl]-2,5 diphenyl tetrazolium bromide
NAXIB: NADPH oxidase inhibitors
NOXs: NADPH oxidases
PBC: primary biliary cholangitis
PMA: phorbol myristate acetate
PTSA: *p*-toluenesulfonic acid
PyBOP: benzotriazol-1-yloxytripyrrolidinophosphonium hexafluorophosphate
Rac1: Ras-related C3 botulinum toxin substrate 1
Ras: rat sarcoma virus
ROS: reactive oxygen species
rtPCR: real-time polymerase chain reaction
SAR: structure–activity relationship
TEA: triethylamine
THF: tetrahydrofuran
