# Supplementary material for: Design of Benzyl-triazolopyrimidine-Based NADPH Oxidase Inhibitors Leads to the Discovery of a Potent Dual Covalent NOX2/MAOB Inhibitor
Source: J Med Chem. 2025 Mar 5;68(6):6292–311. doi: 10.1021/acs.jmedchem.4c02644 (PMC11956017; doi:10.1021/acs.jmedchem.4c02644)
Supplement: Supplementary file 1 — jm4c02644_si_001.pdf [file jm4c02644_si_001.pdf]

Supporting Information

The Design of Benzyltriazolopyrimidine-based NADPH Oxidase Inhibitors Leads to the Discovery of a Potent Dual Covalent NOX2/MAOB Inhibitor

Beatrice Noce,[a,¶] Sara Marchese,[b,¶] Marta Massari,[b,¶] Chiara Lambona,[a,¶] Joana Reis,[b] Francesco Fiorentino,[a] Alessia Raucci,[a] Rossella Fioravanti,[a] Mariana Castelôa,[c] Alessandro Mormino,[d] Stefano Garofalo,[d] Cristina Limatola,[d] Lorenzo Basile,[b] Andrea Gottinger,[b] Claudia Binda,[b] Andrea Mattevi,[b,*] Antonello Mai,[a,*] and Sergio Valente,[a,*]

[a]*Department of Drug Chemistry and Technologies, Sapienza University of Rome, P.le Aldo Moro 5, 00185 Rome, Italy*

[b]*Department of Biology and Biotechnology Lazzaro Spallanzani, University of Pavia, Via Adolfo Ferrata 9A, 27100 Pavia, Italy*

[c]*CIQUP-IMS/Department of Chemistry and Biochemistry, Faculty of Sciences, University of Porto, Rua do Campo Alegre s/n, 4169-007 Porto, Portugal*

[d]*Department of Physiology and Pharmacology, Sapienza University of Rome, P.le Aldo Moro 5, 00185 Rome, Italy*

* Corresponding authors:

(A.Mattevi) E-mail address: andrea.mattevi@unipv.it, (A. Mai) E-mail address: antonello.mai@uniroma1.it, (S. Valente) E-mail address: sergio.valente@uniroma1.it

Table of contents

Table S1 .... S3

Table S2 .... S9

^{1}H-NMR OF FINAL COMPOUNDS S10

HPLC TRACES FOR COMPOUNDS **4**, **7d**, and **9a** S27

Figure S1 .... S30

Figure S2 .... S30

Figure S3 .... S31

Figure S4 .... S32

Figure S5 .... S33

Figure S6 .... S34

Figure S7 .... S35

Figure S8 .... S35

Figure S9 .... S36

Figure S10 .... S36

Figure S11 .... S38

Table S3 .... S39

Figure S12 .... S40

Figure S13 .... S41

Figure S14 .... S42

Figure S15 .... S43

Figure S16 .... S44

Table S1. Chemical and physical data for compounds **11-19**, **20a,b**, **21a,b**, **22-26**, **27a-f**, **28a-f**

Cmpd	Structure	mp, °C	Recryst. solvent[a]	Yield, %
**11**	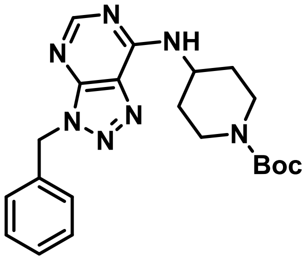	148-150	b	40.2
**12**	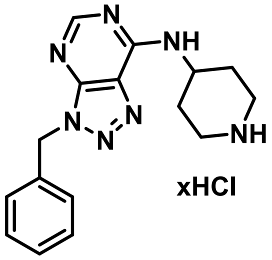	>250	f	77.9
**13**	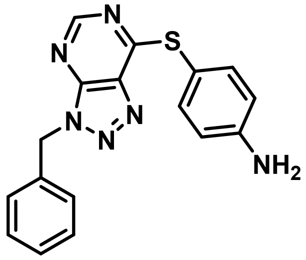	178-180	d	99.8
**14**	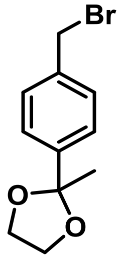	Oil	-	99.9
**15**	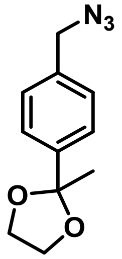	Oil	-	74.8
**16**	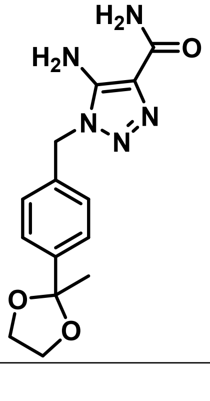	214-216	e	70.4

**17**	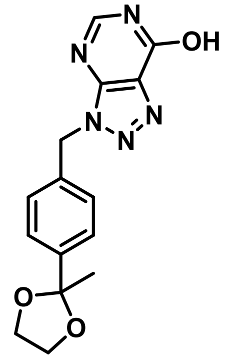	185-187	d	99.8
**18**	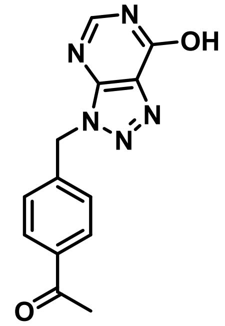	>250	f	72.2
**19**	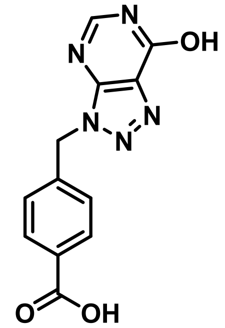	>250	f	45.0
**20a**	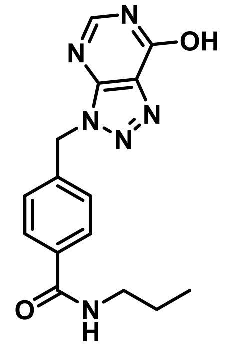	198-200	d	27.2
**20b**	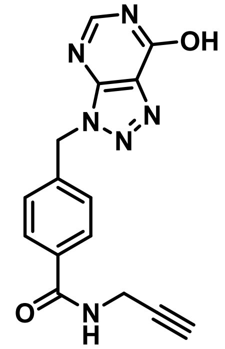	220-222	e	40.0

**21a**	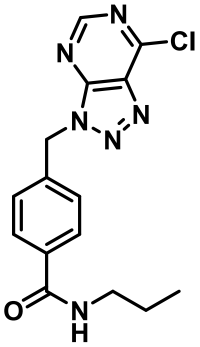	98-100	a	90.0
**21b**	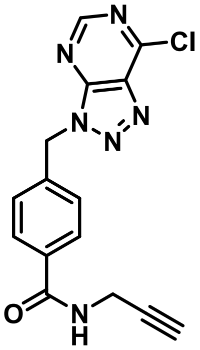	116-118	a	98.1
**22**	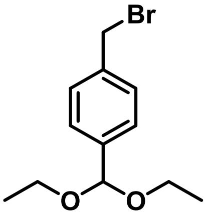	Oil	-	95.5
**23**	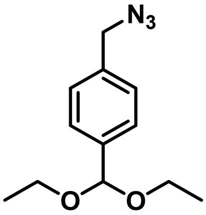	Oil	-	88.7
**24**	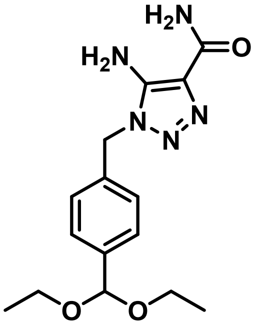	140-142	b	87.4
**25**	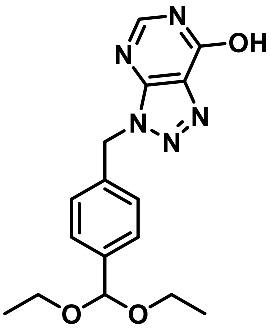	173-175	d	99.5

**26**	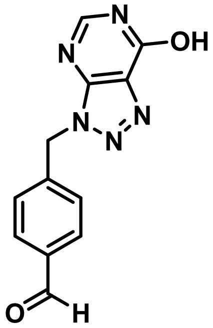	>250	f	97.7
**27a**	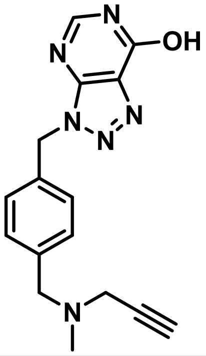	185-187	d	83.0
**27b**	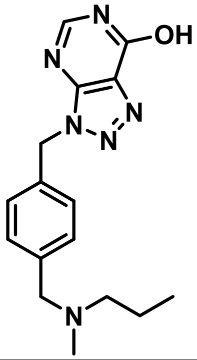	98-100	a	61.1
**27c**	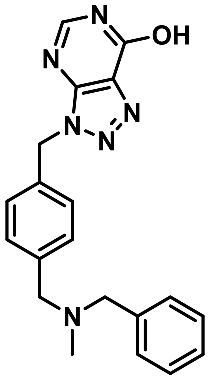	190-192	d	75.3
**27d**	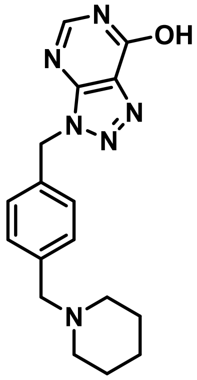	188-190	d	78.7

**27e**	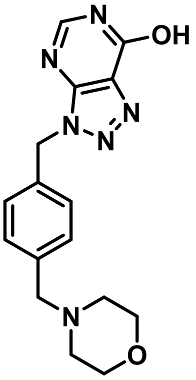	197-199	d	89.9
**27f**	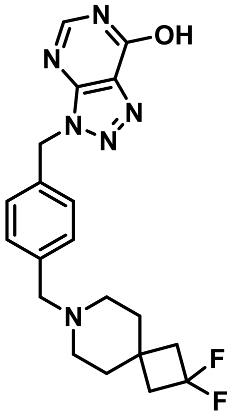	206-208	e	81.9
**28a**	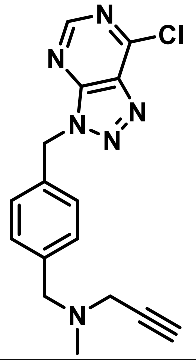	107-109	a	38.2
**28b**	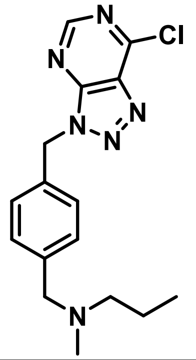	Oil	-	47.8

**28c**	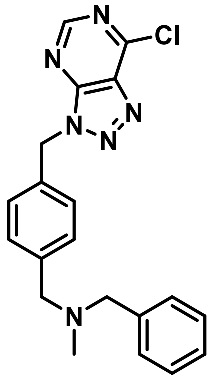	Oil	-	75.3
**28d**	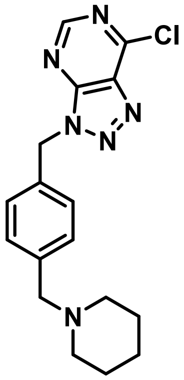	Oil	-	54.1
**28e**	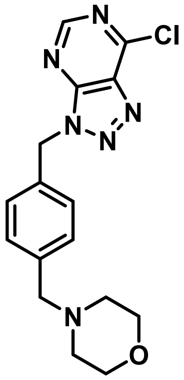	Oil	-	39.9
**28f**	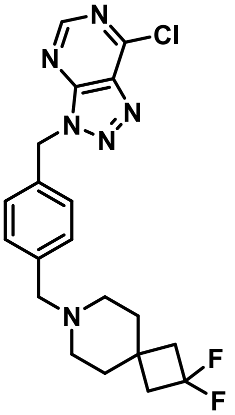	Oil	-	64.1
[a]a: cyclohexane/toluene; b: toluene; c: toluene/acetonitrile; d: acetonitrile; e: acetonitrile/methanol; f: methanol.				

**Table S2.** Elemental analysis for final compounds **2-6**, **7a-d**, **8a,b**, **9a-f**															
		calculated, %							found, %						
**No.**	**MW**	**C**	**N**	**O**	**H**	**Cl**	**S**	**F**	**C**	**N**	**O**	**H**	**Cl**	**S**	**F**
**2**	305.31	47.21	22.94	15.72	3.63		10.50		47.31	22.99	15.74	3.65		10.52	
**3**	245.67	53.78	28.51		3.28	14.43			53.86	28.47		3.29	14.41		
**4**	226.24	58.40	37.15		4.46				58.48	37.11		4.47			
**5**	395.47	60.74	24.79	8.09	6.37				60.84	24.74	8.05	6.38			
**6**	331.81	54.30	29.55		5.47	10.68			54.38	29.51		5.48	10.66		
**7a**	349.40	61.88	28.06	4.58	5.48				61.96	28.02	4.64	5.49			
**7b**	337.39	60.52	29.06	4.74	5.68				60.60	29.02	4.70	5.69			
**7c**	363.43	62.79	26.98	4.40	5.82				62.87	26.94	4.35	5.83			
**7d**	388.45	61.84	21.64	4.12	4.15		8.25		61.91	21.60	4.16	4.16		8.27	
**8a**	445.50	59.31	22.01	7.18	4.30		7.20		59.38	21.97	7.21	4.31		7.17	
**8b**	441.47	59.86	22.21	7.25	3.42		7.26		59.96	22.16	7.21	3.43		7.24	
**9a**	441.51	62.57	22.21	3.62	4.34		7.26		62.64	22.17	3.59	4.35		7.23	
**9b**	445.55	62.00	22.01	3.59	5.20		7.20		62.07	21.97	3.57	5.21		7.24	
**9c**	493.59	65.70	19.86	3.24	4.70		6.50		65.78	19.82	3.21	4.71		6.47	
**9d**	457.56	63.00	21.43	3.50	5.07		7.01		63.08	21.39	3.52	5.08		7.04	
**9e**	459.53	60.12	21.34	6.96	4.61		6.98		60.20	21.30	6.99	4.62		6.95	
**9f**	533.60	60.78	18.37	3.00	4.72		6.01	7.12	60.86	18.33	2.97	4.73		6.02	7.11

^{1}H-NMR OF FINAL COMPOUNDS (2-6, 7a-d, 8a,b, 9a-f) and HPLC for compound MC4762

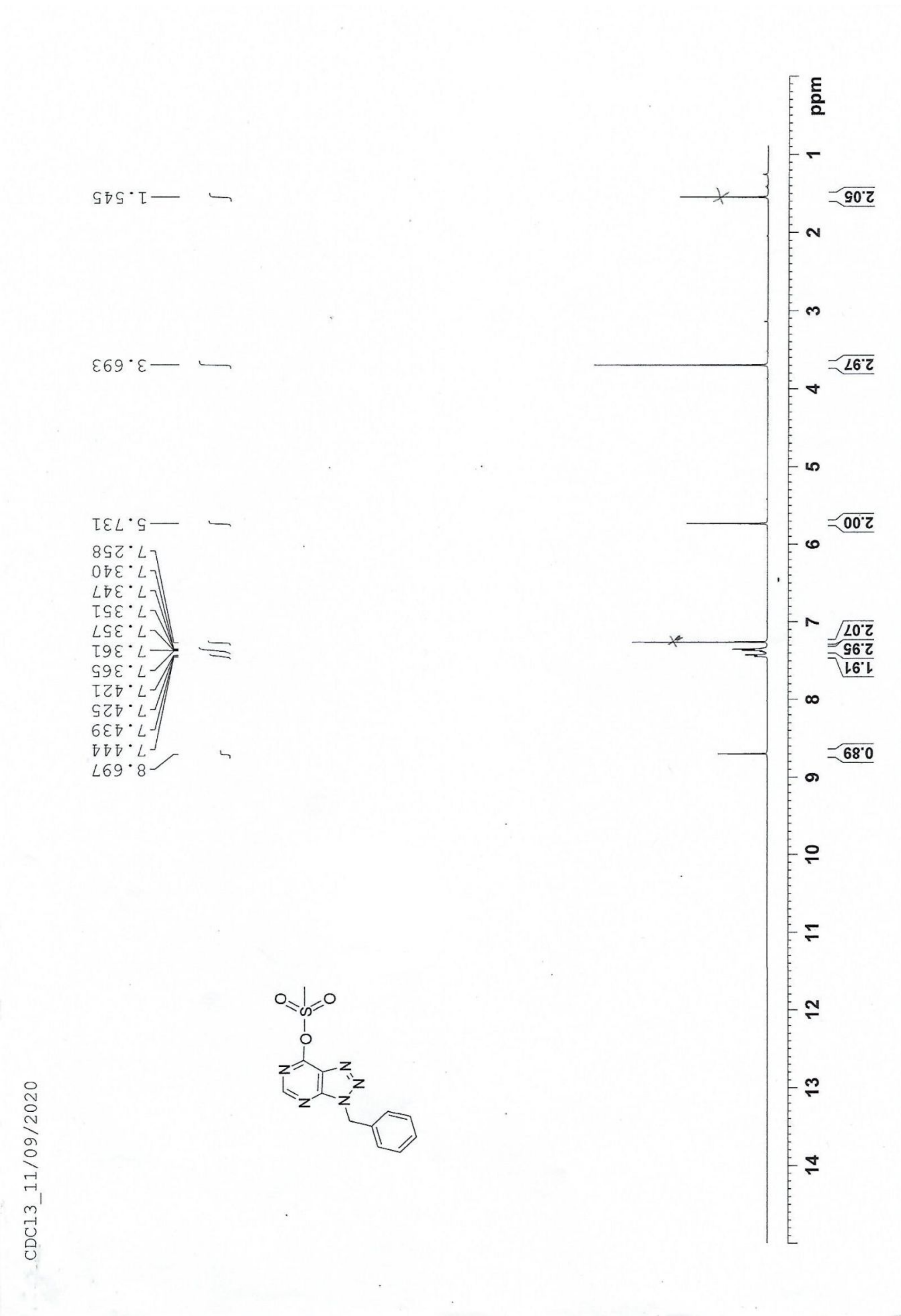

^{1}H-NMR of final compound 2 (MC4553)

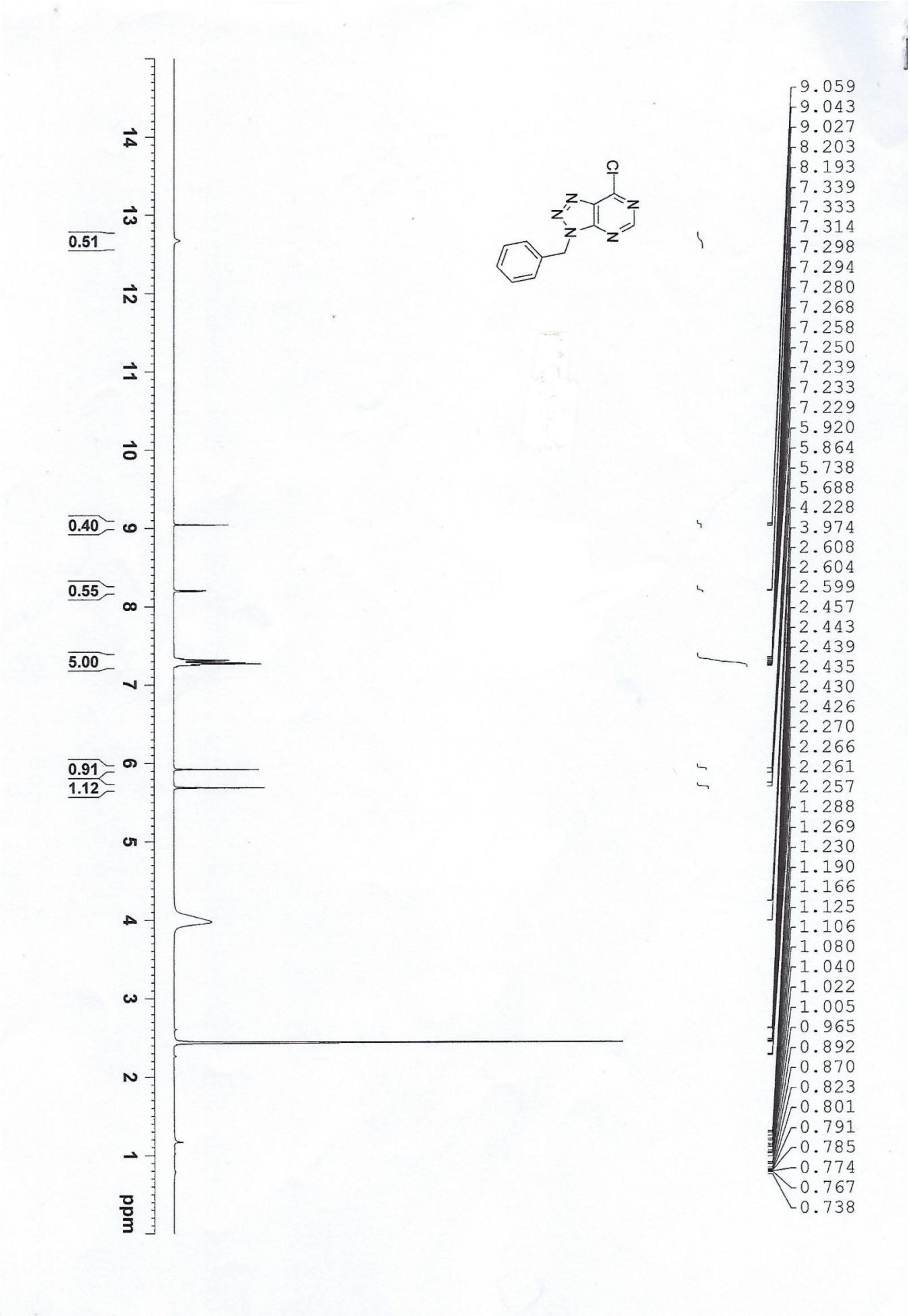

^{1}H-NMR of final compound 3 (MC4551)

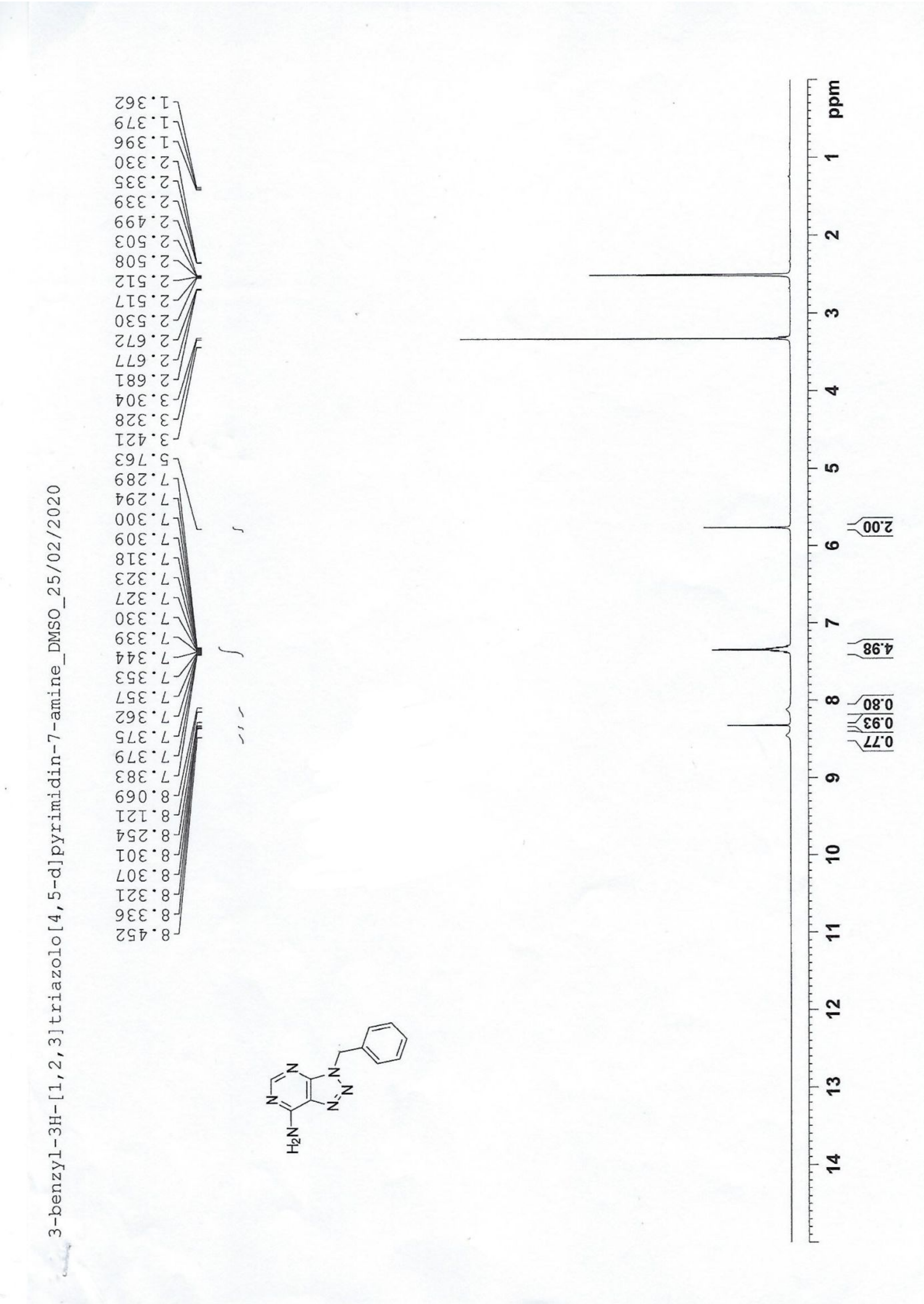

^{1}H-NMR of final compound 4 (MC4550)

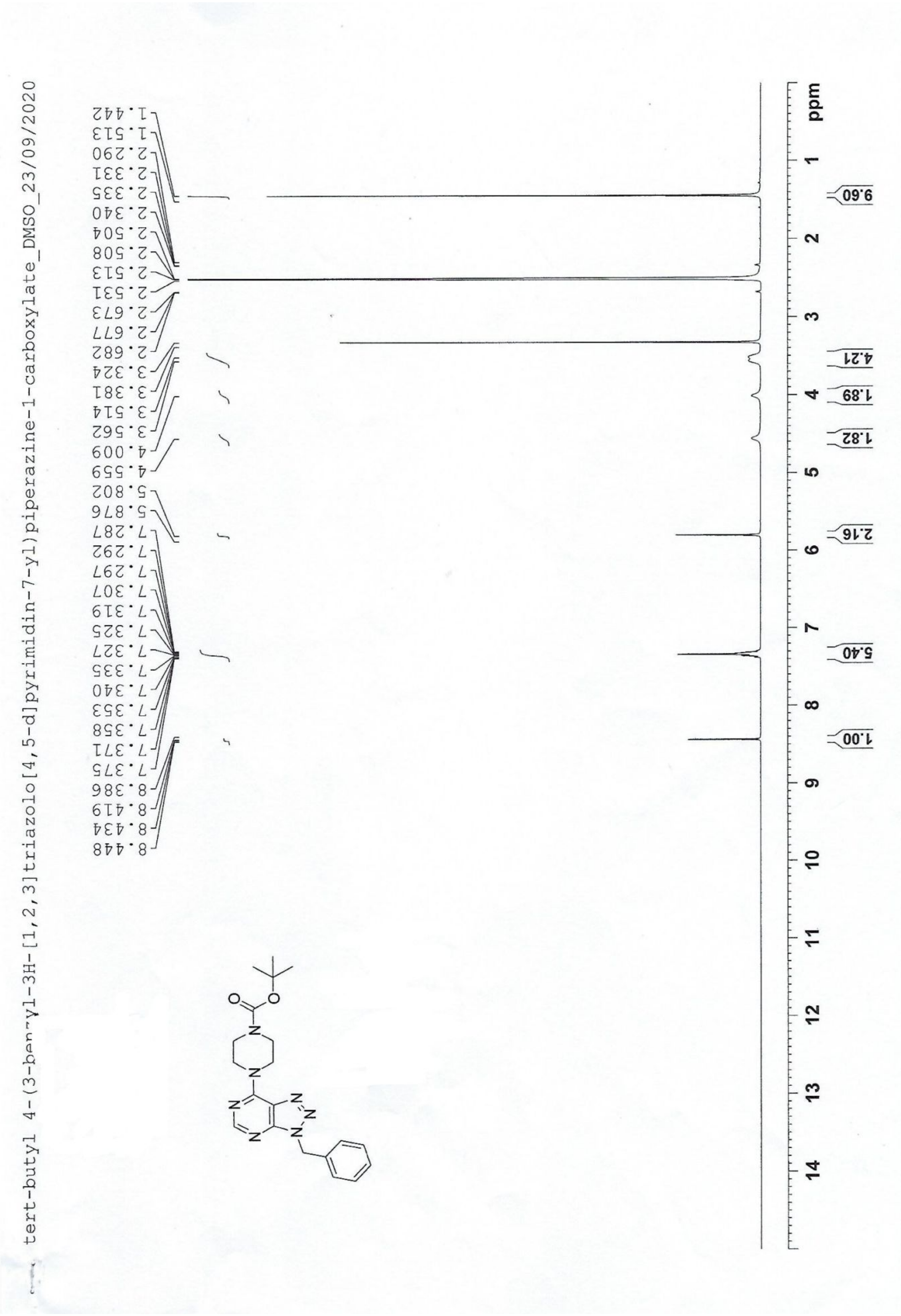

^{1}H-NMR of final compound 5 (MC4606)

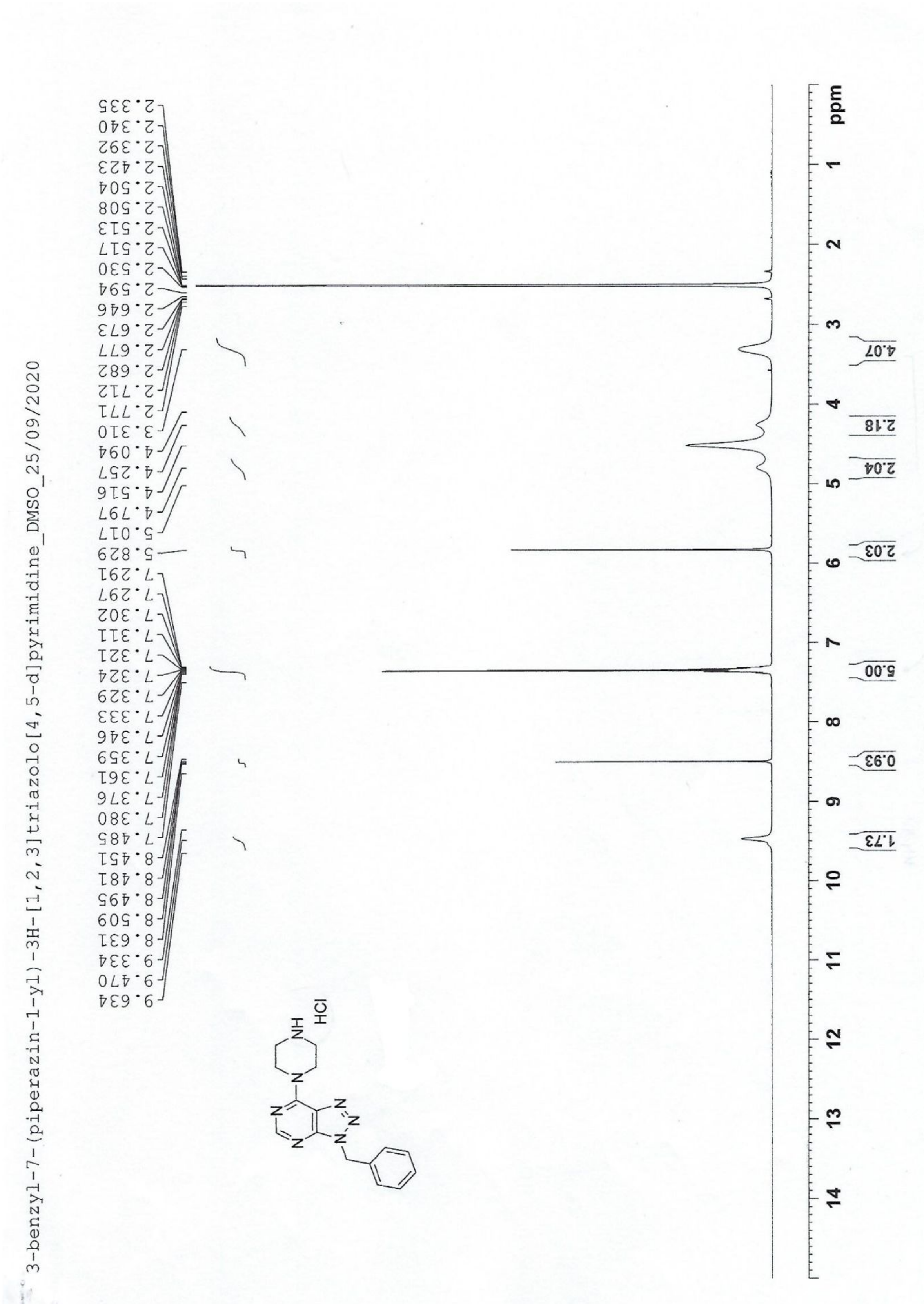

^{1}H-NMR of final compound 6 (MC4596)

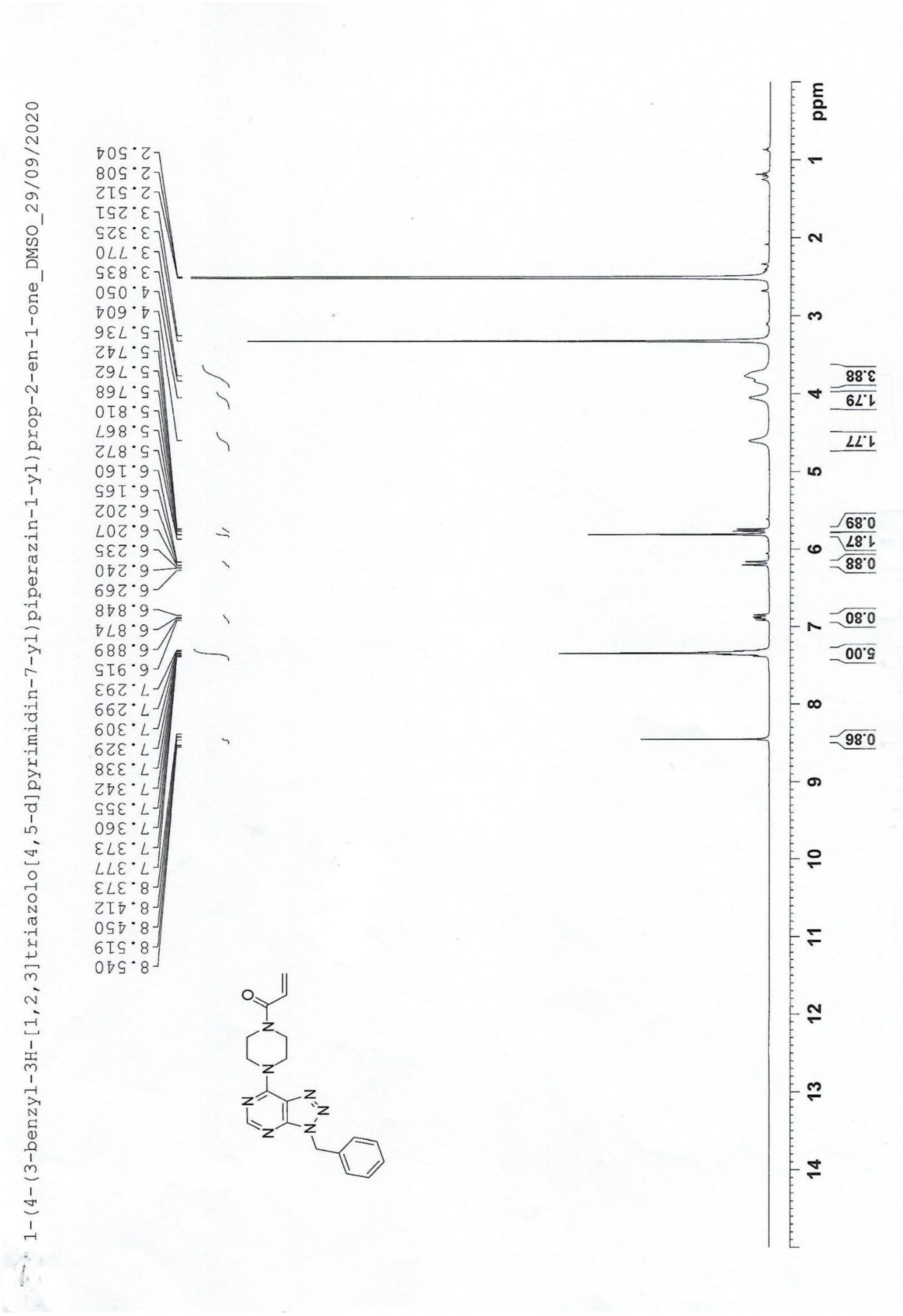

^{1}H-NMR of final compound 7a (MC4559)

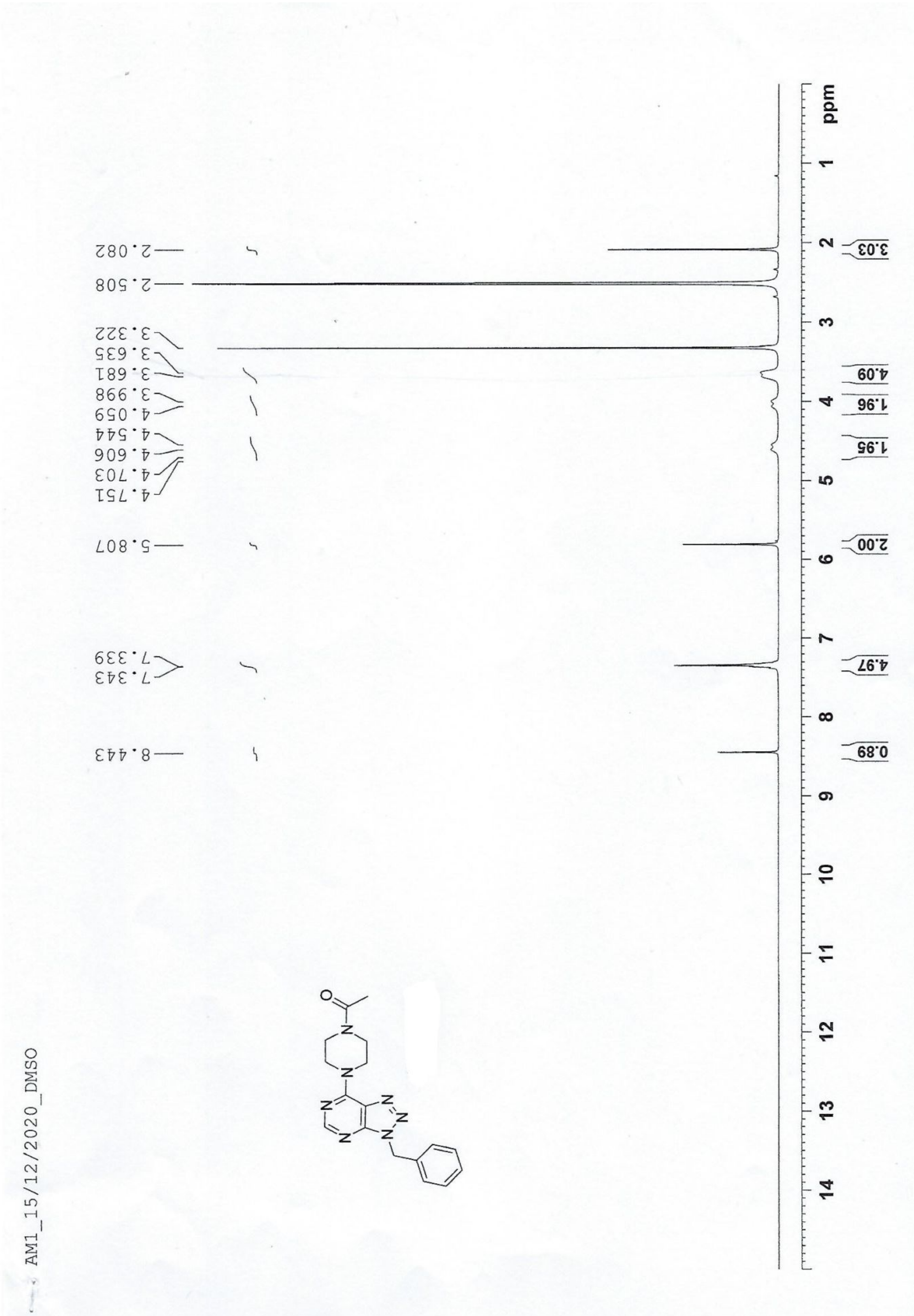

^{1}H-NMR of final compound 7b (MC4599)

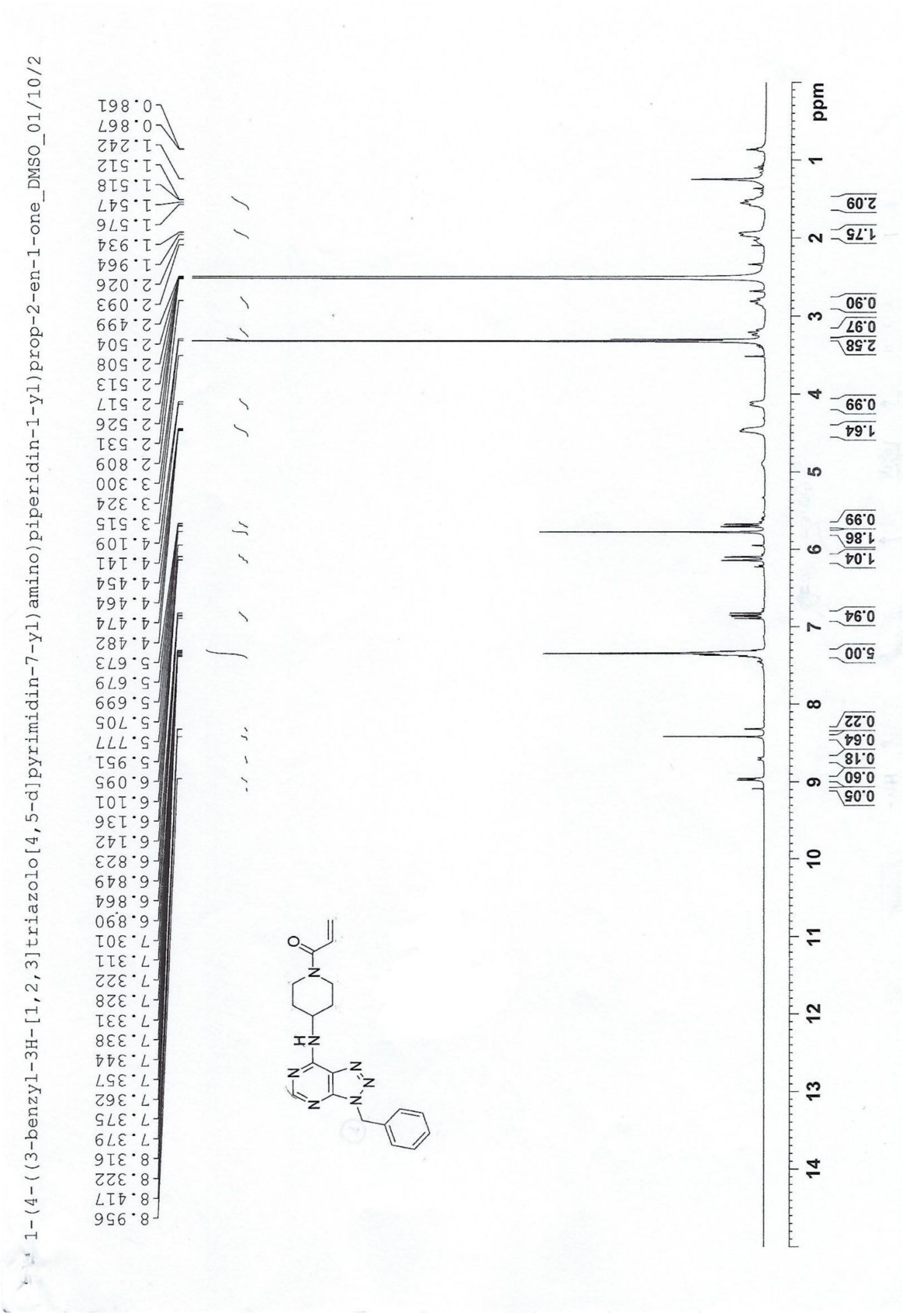

^{1}H-NMR of final compound 7c (MC4571)

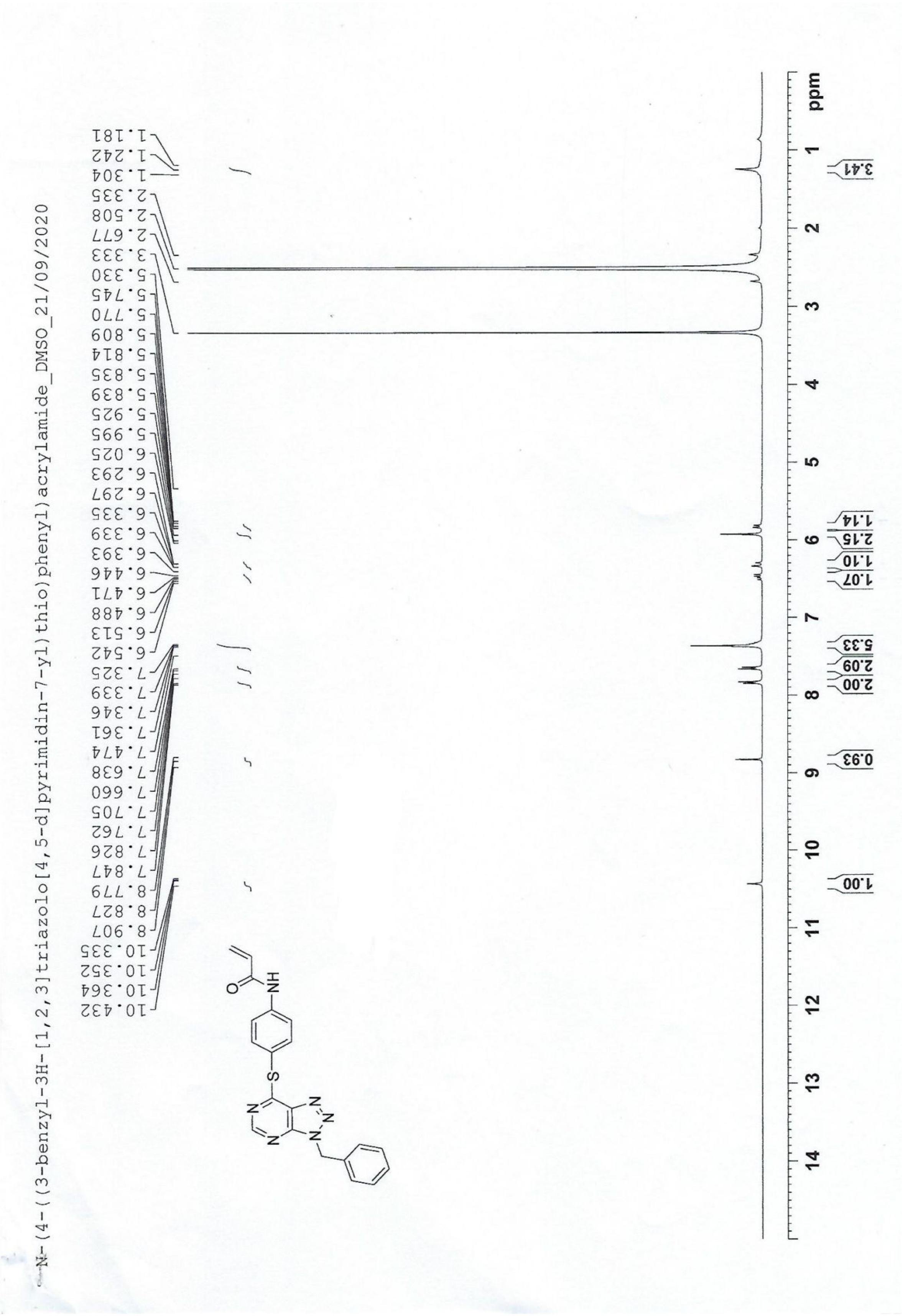

^{1}H-NMR of final compound 7d (MC4554)

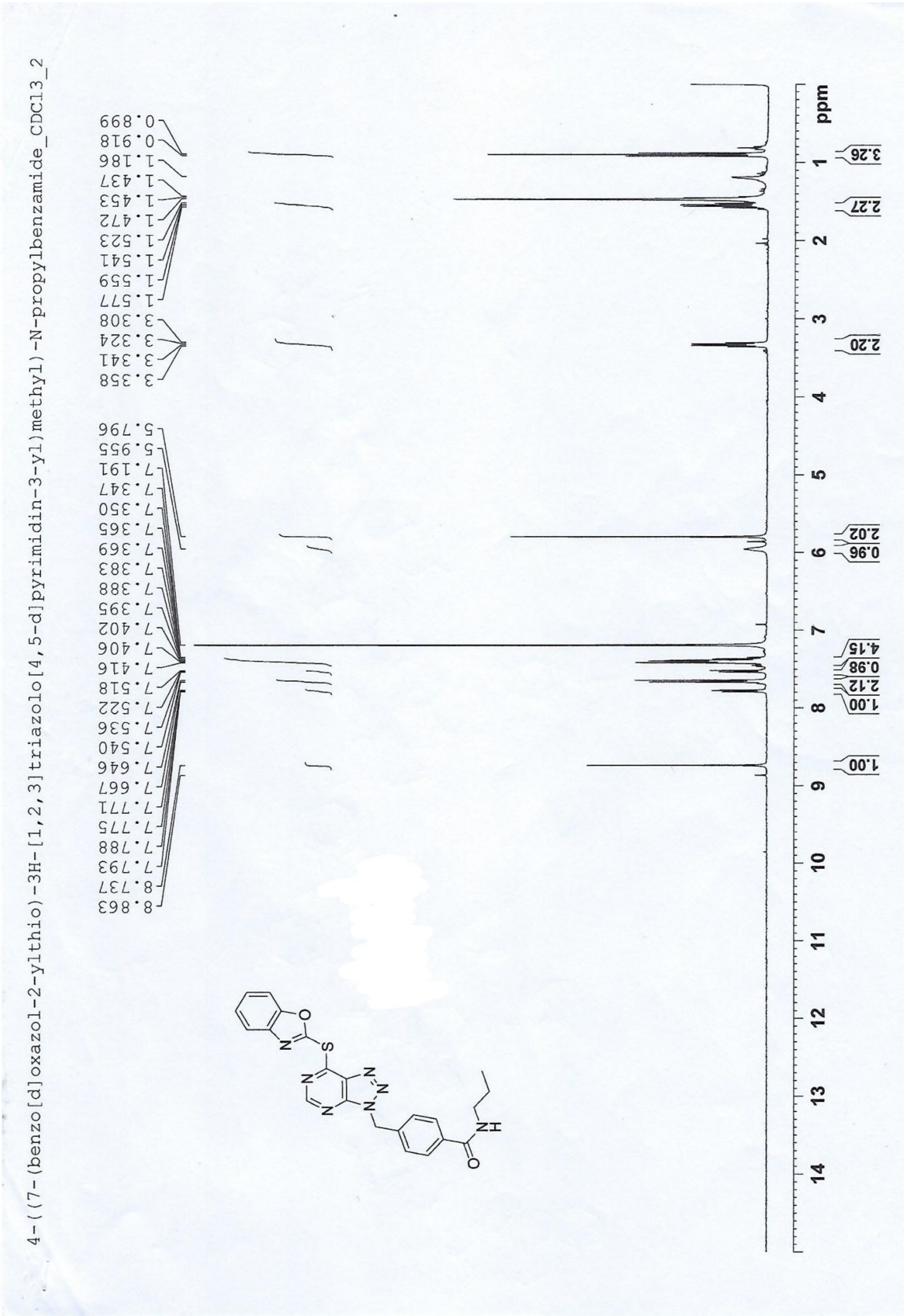

^{1}H-NMR of final compound 8a (MC4768)

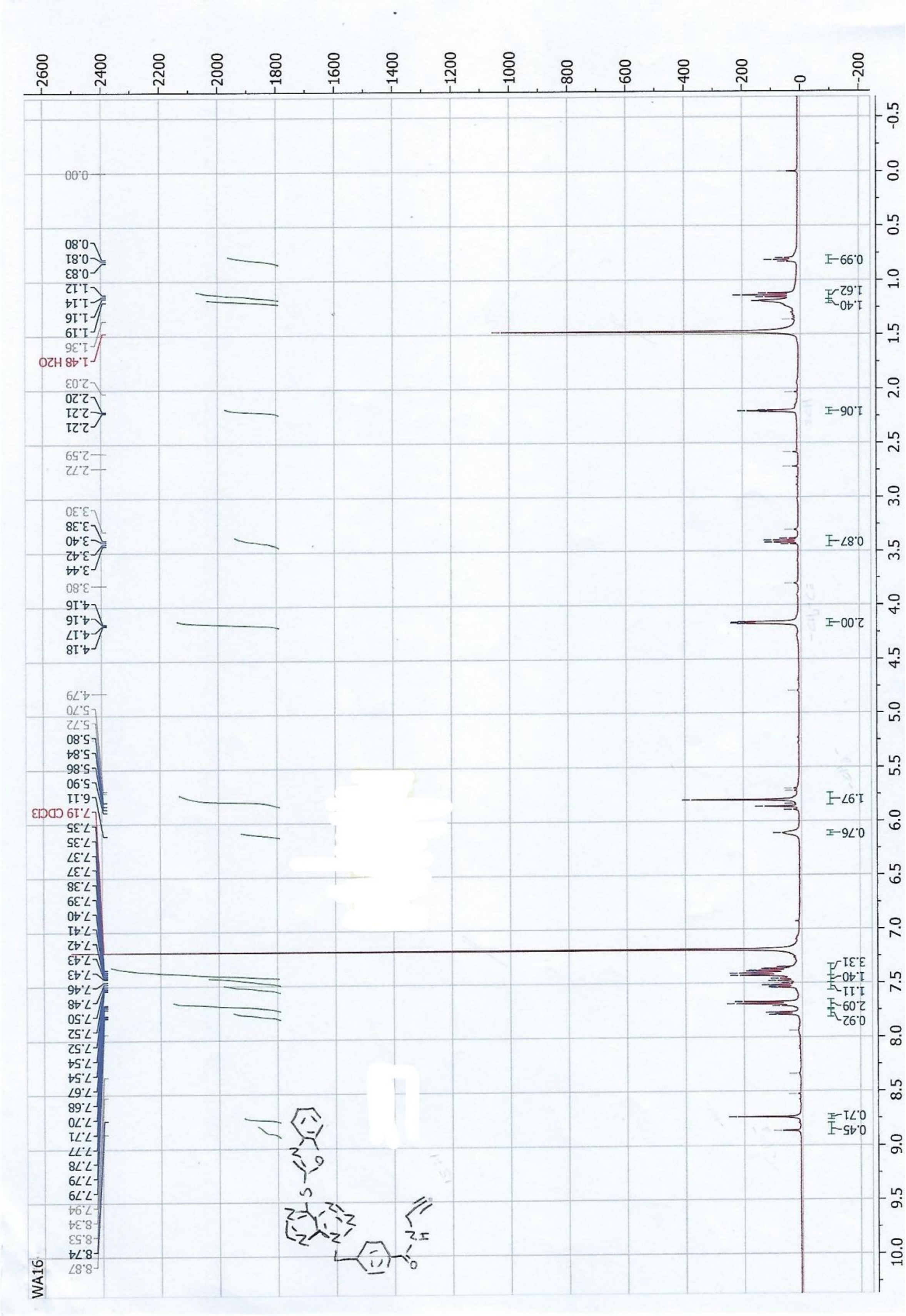

^{1}H-NMR of final compound 8b (MC4767)

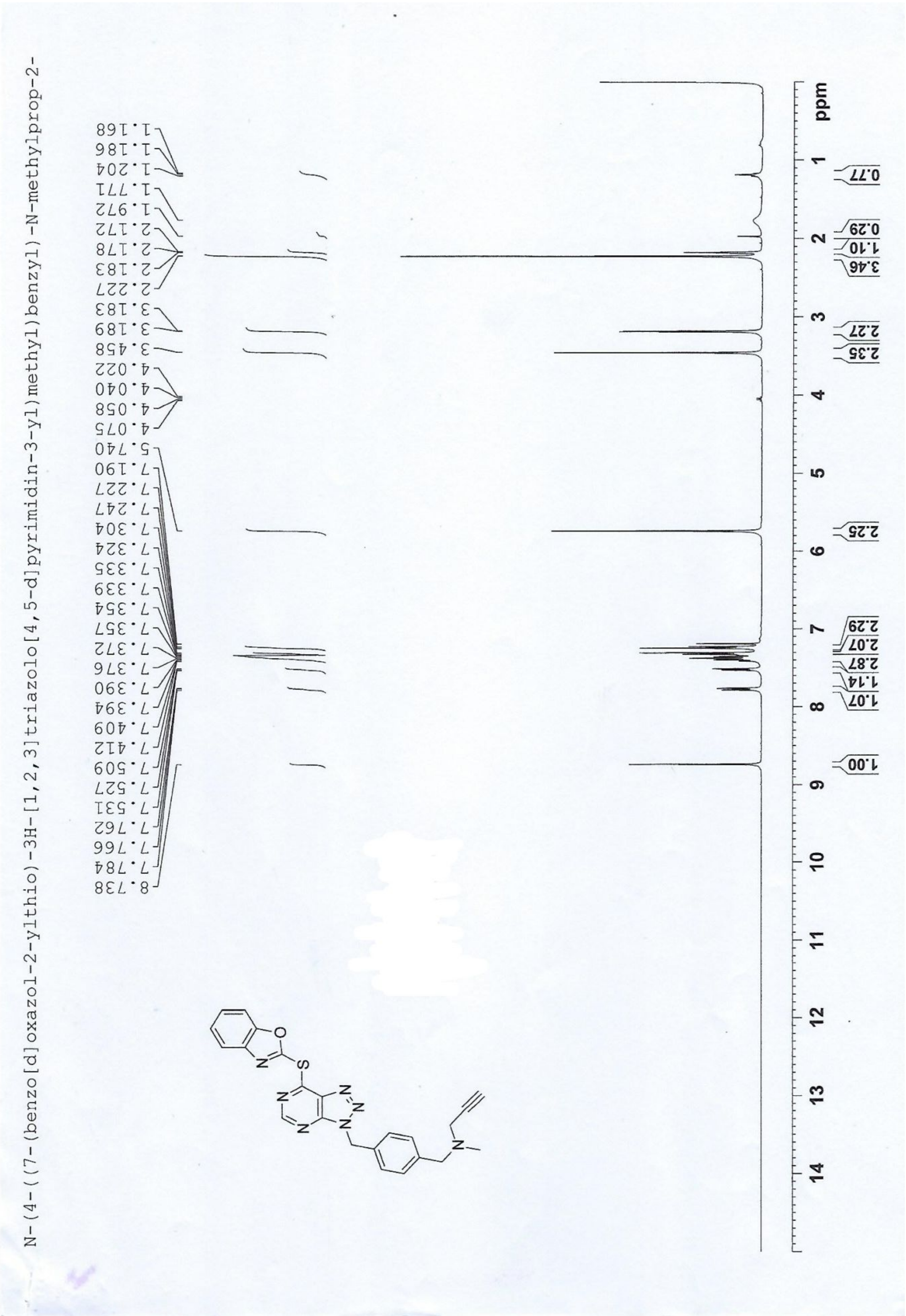

^{1}H-NMR of final compound 9a (MC4762)

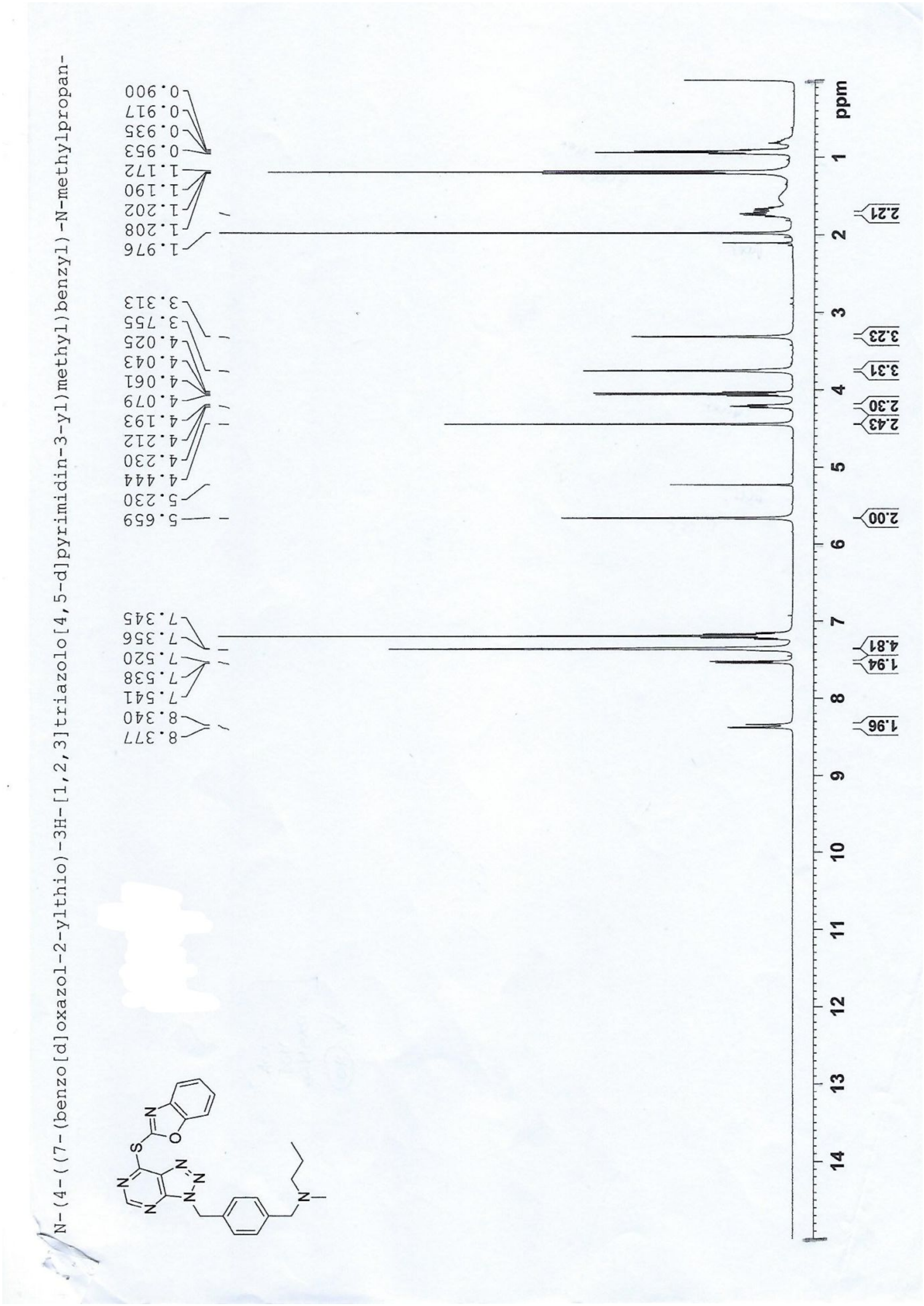

^{1}H-NMR of final compound 9b (MC4998)

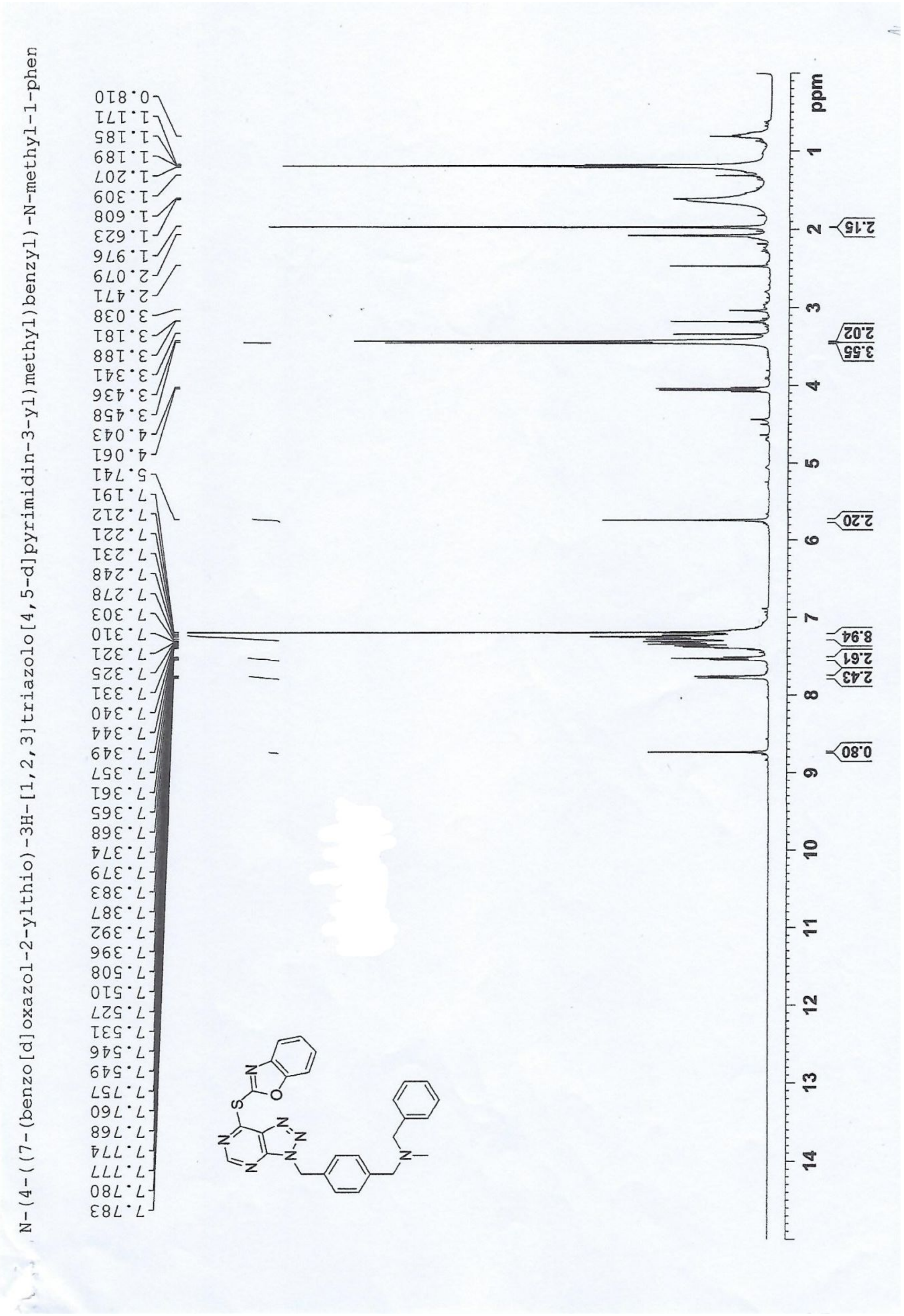

^{1}H-NMR of final compound 9c (MC4999)

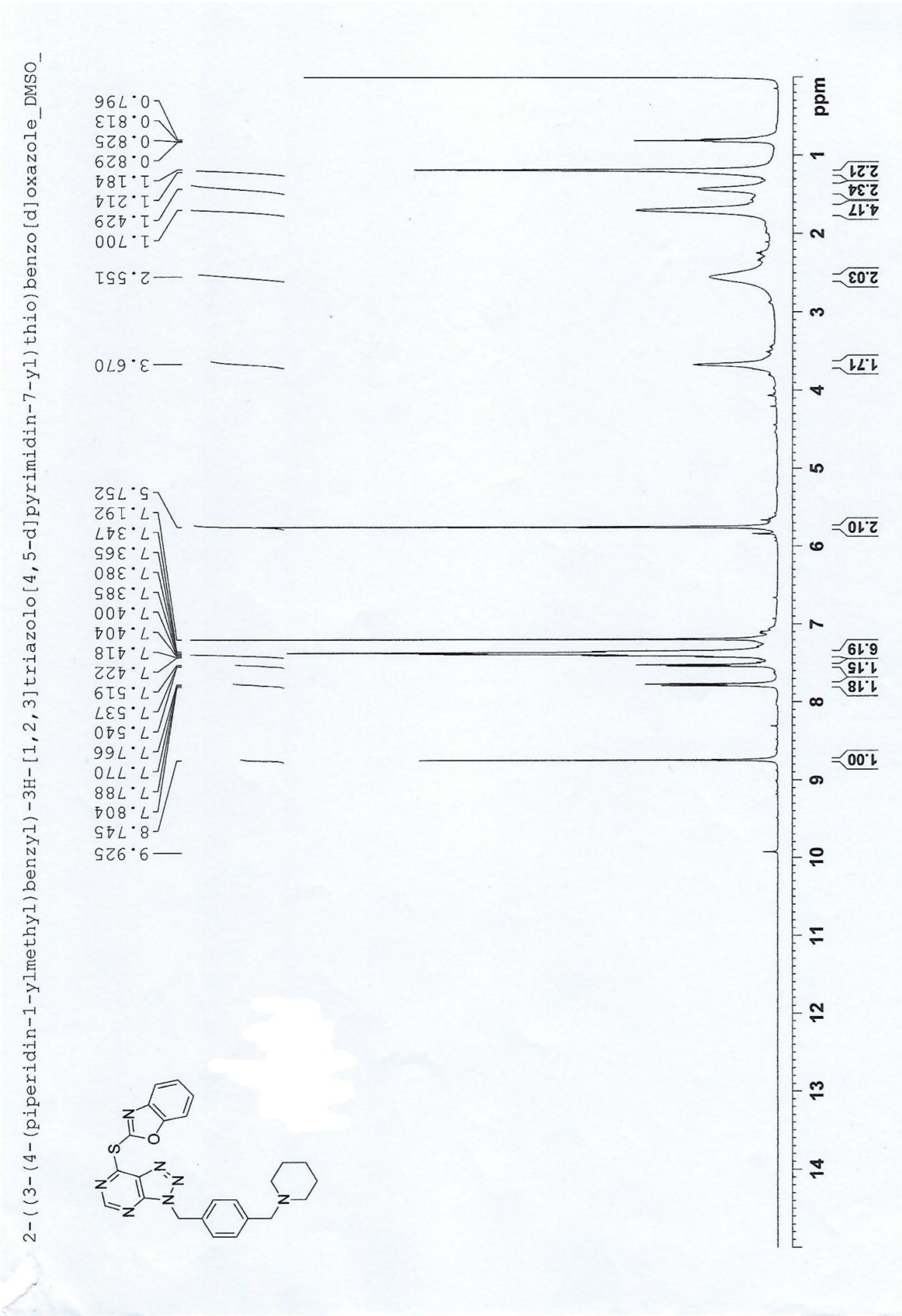

^{1}H-NMR of final compound 9d (MC5018)

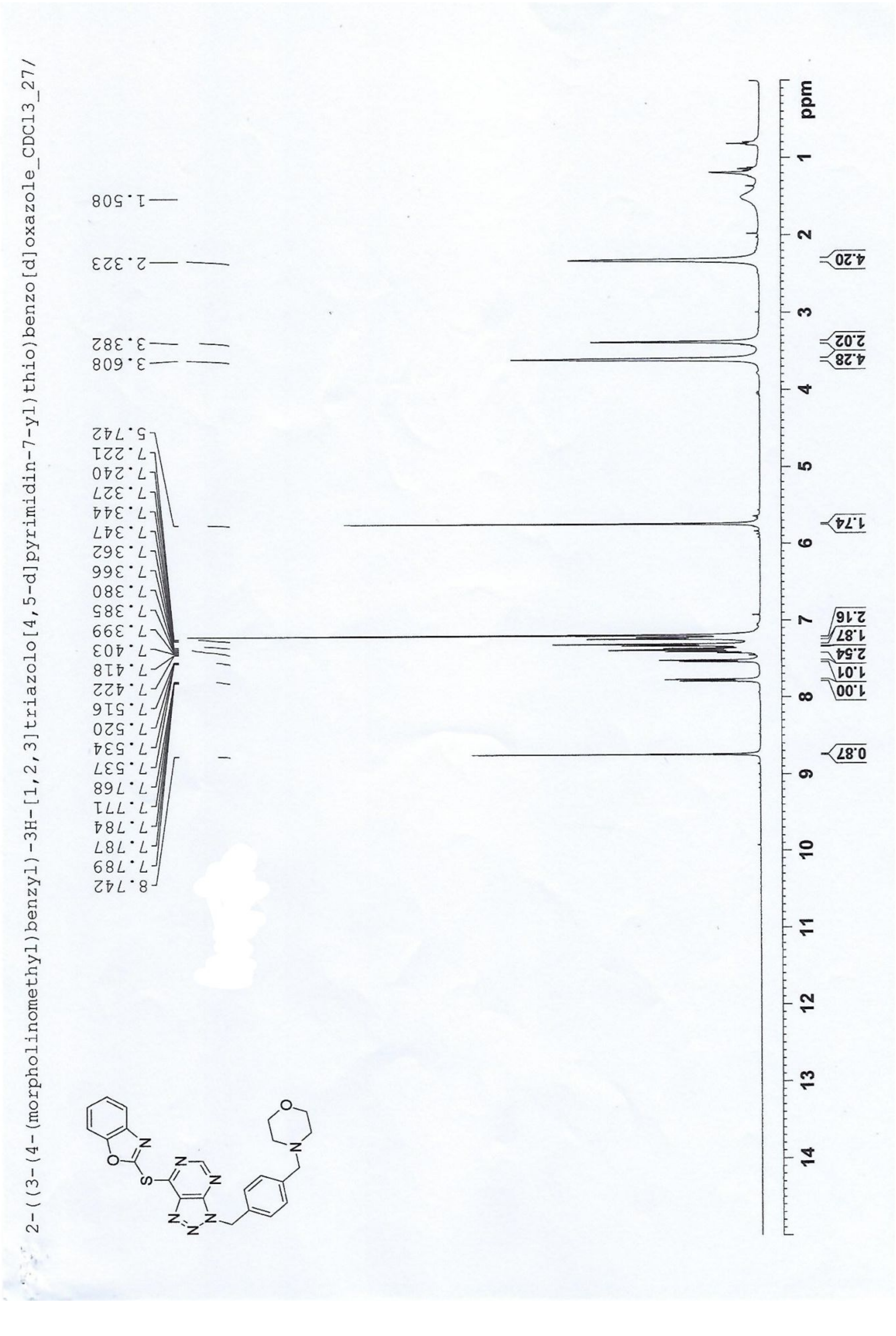

^{1}H-NMR of final compound 9e (MC4982)

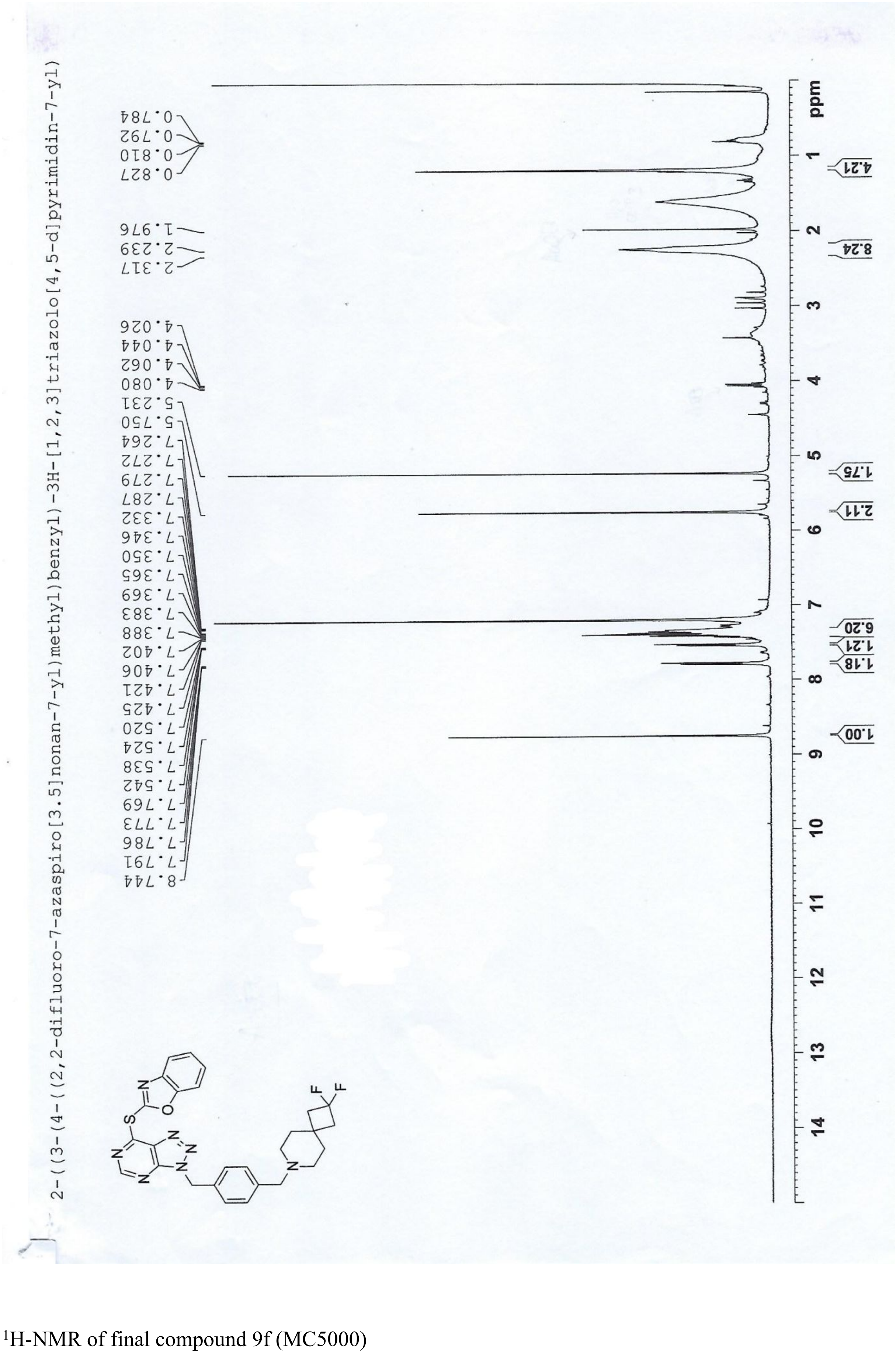

^{1}H-NMR of final compound 9f (MC5000)

HPLC TRACES FOR COMPOUNDS 4, 7d, and 9a

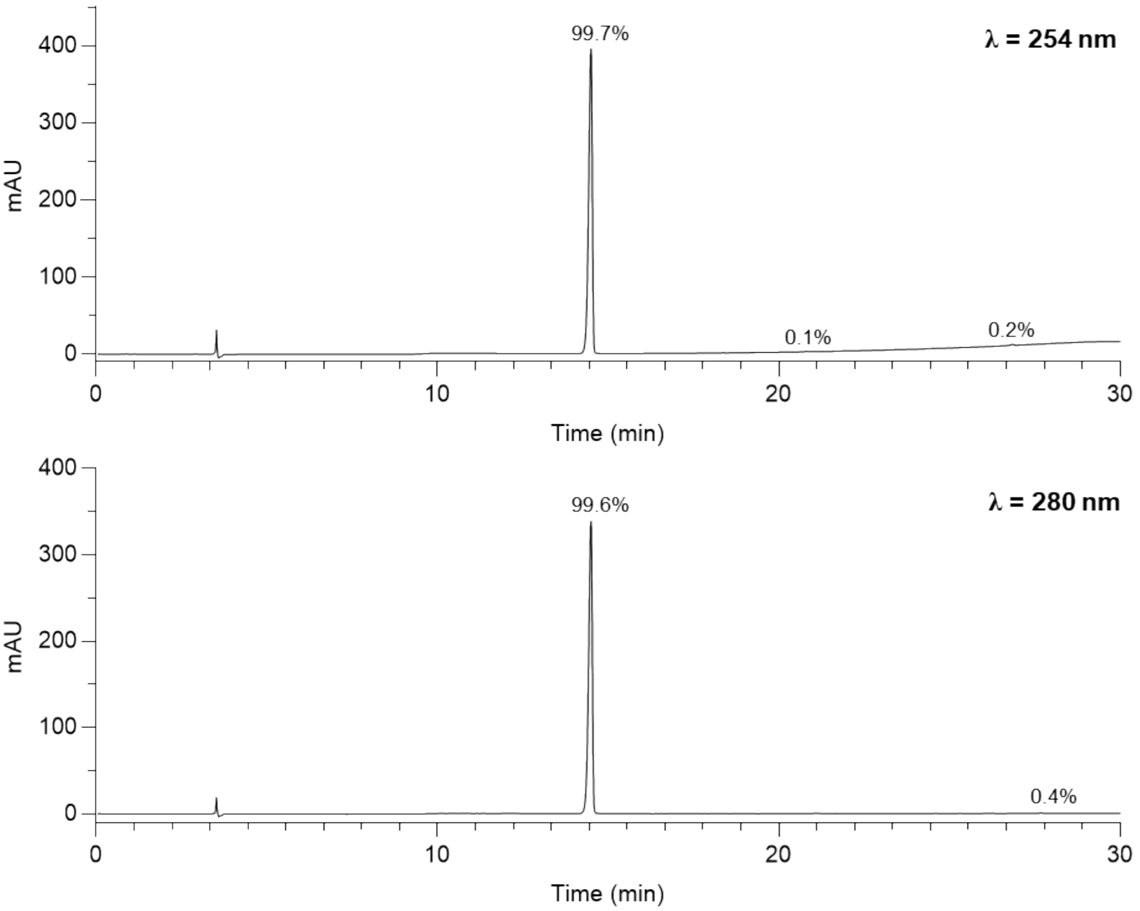

HPLC traces of final compound **4**.

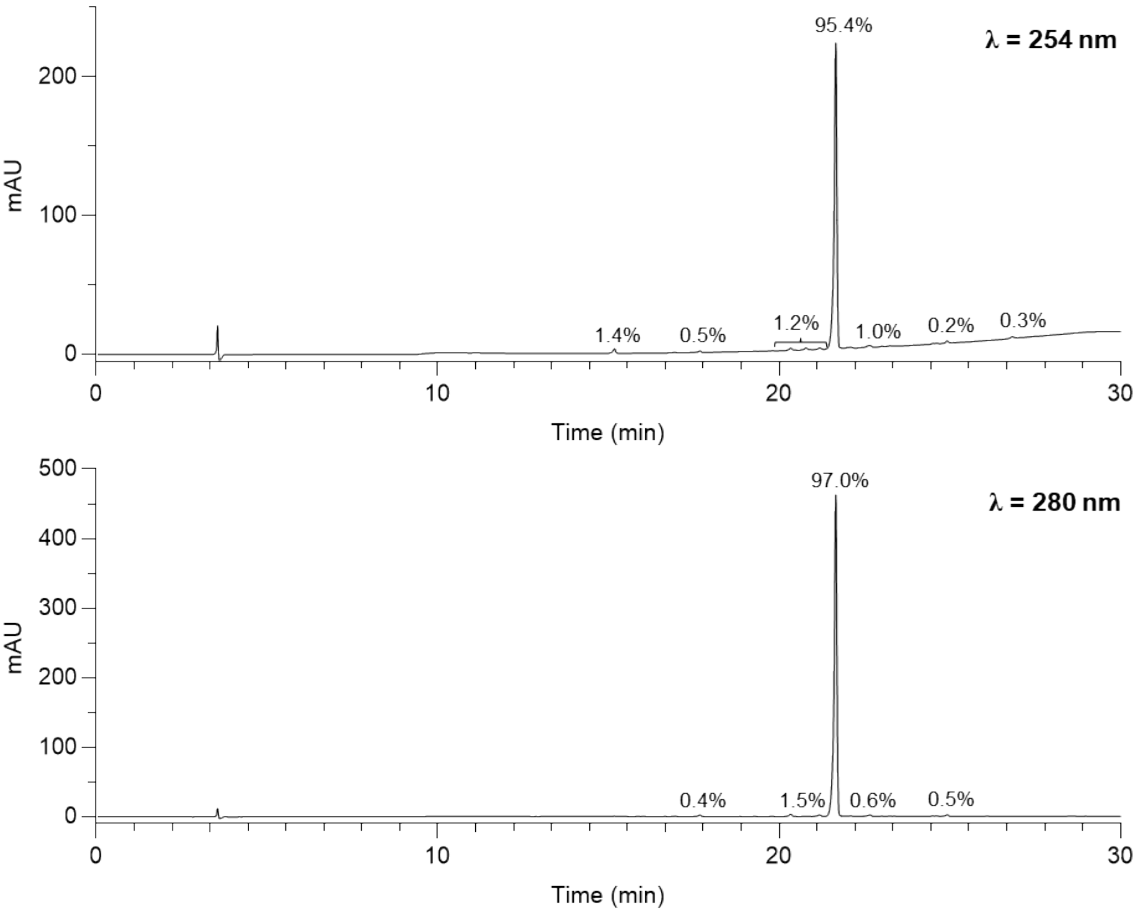

HPLC traces of final compound **7d**.

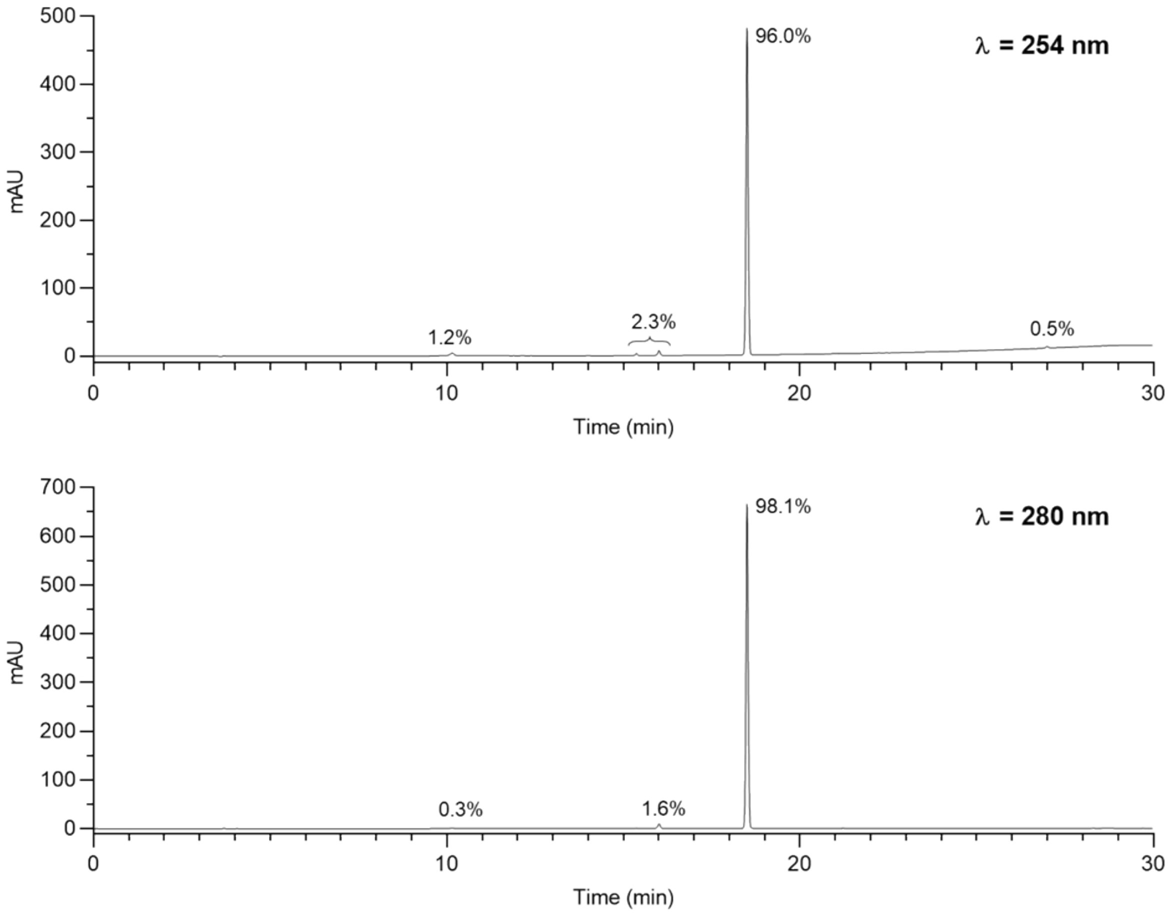

HPLC traces of final compound **9a**.

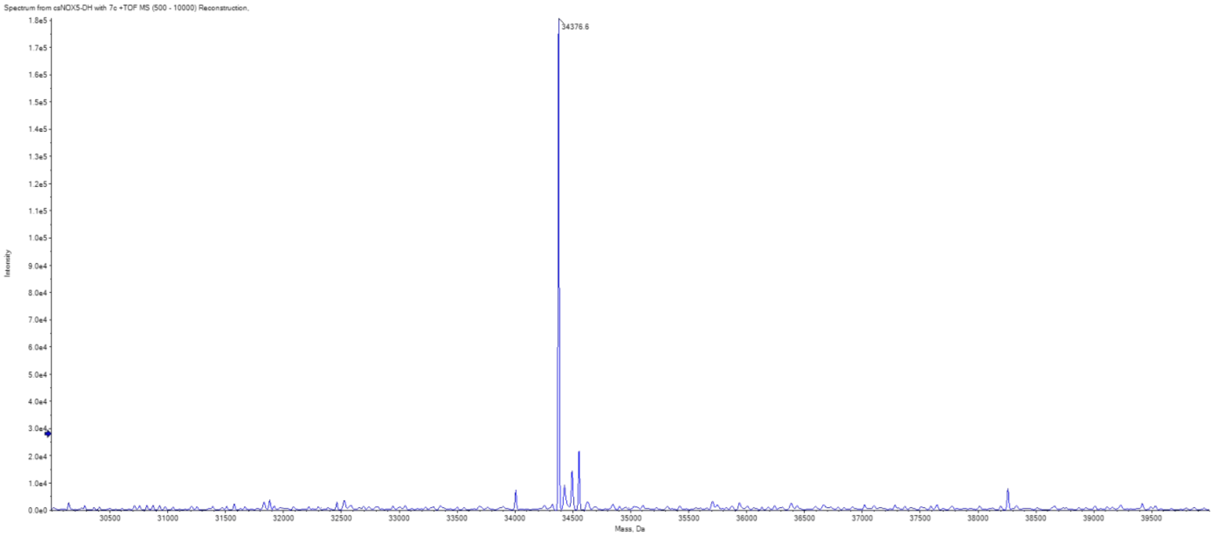

Figure S1. **Intact protein mass spectrometry of the *cs*NOX5-dehydrogenase after reaction with 7c**. The reference spectrum for the native protein is shown in Figure 3.

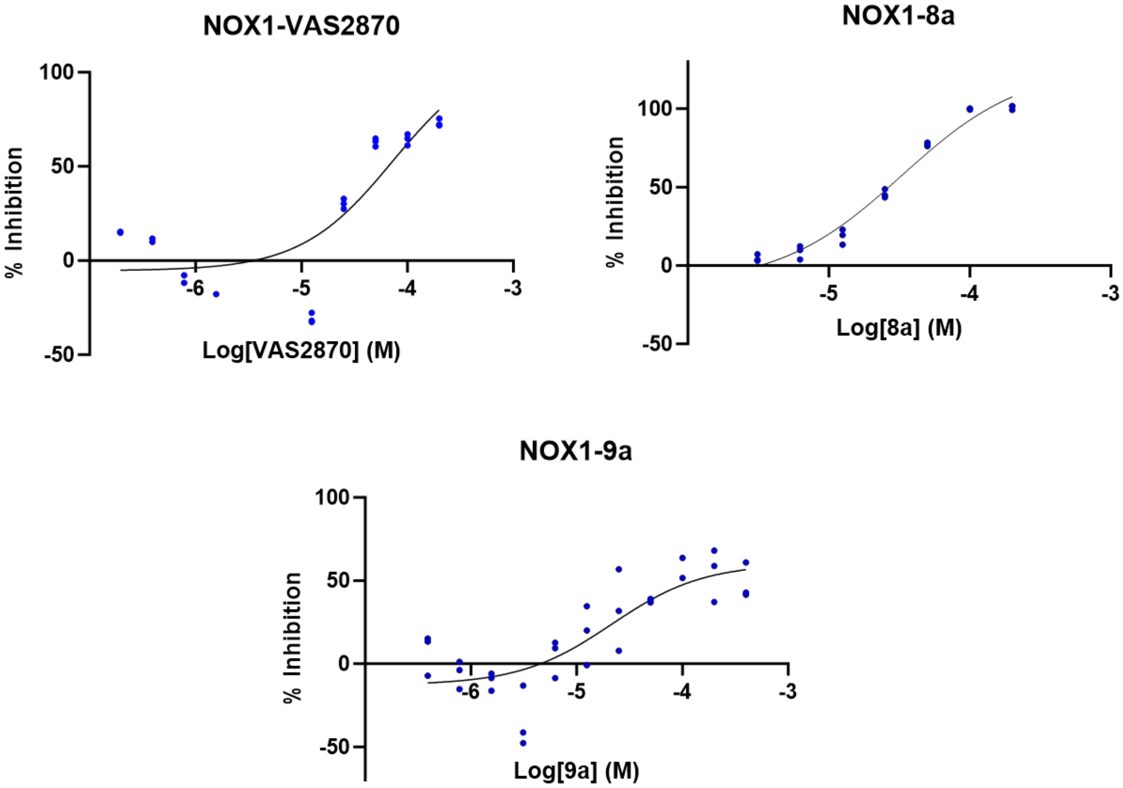

Figure S2. IC_{50} determination for human NOX1 isolated membranes. Progress curves were generated at a constant substrate concentration (40 µM) in the presence of varying inhibitor concentrations (70 nM to 200 µM), plotting inhibition (%) against the logarithm of inhibitor concentration. Reactions were followed using the Amplex Red/horseradish peroxidase assay. IC_{50} values were calculated using nonlinear regression analysis in GraphPad Prism 9.0 (mean ± s.d., n = 3 independent experiments).

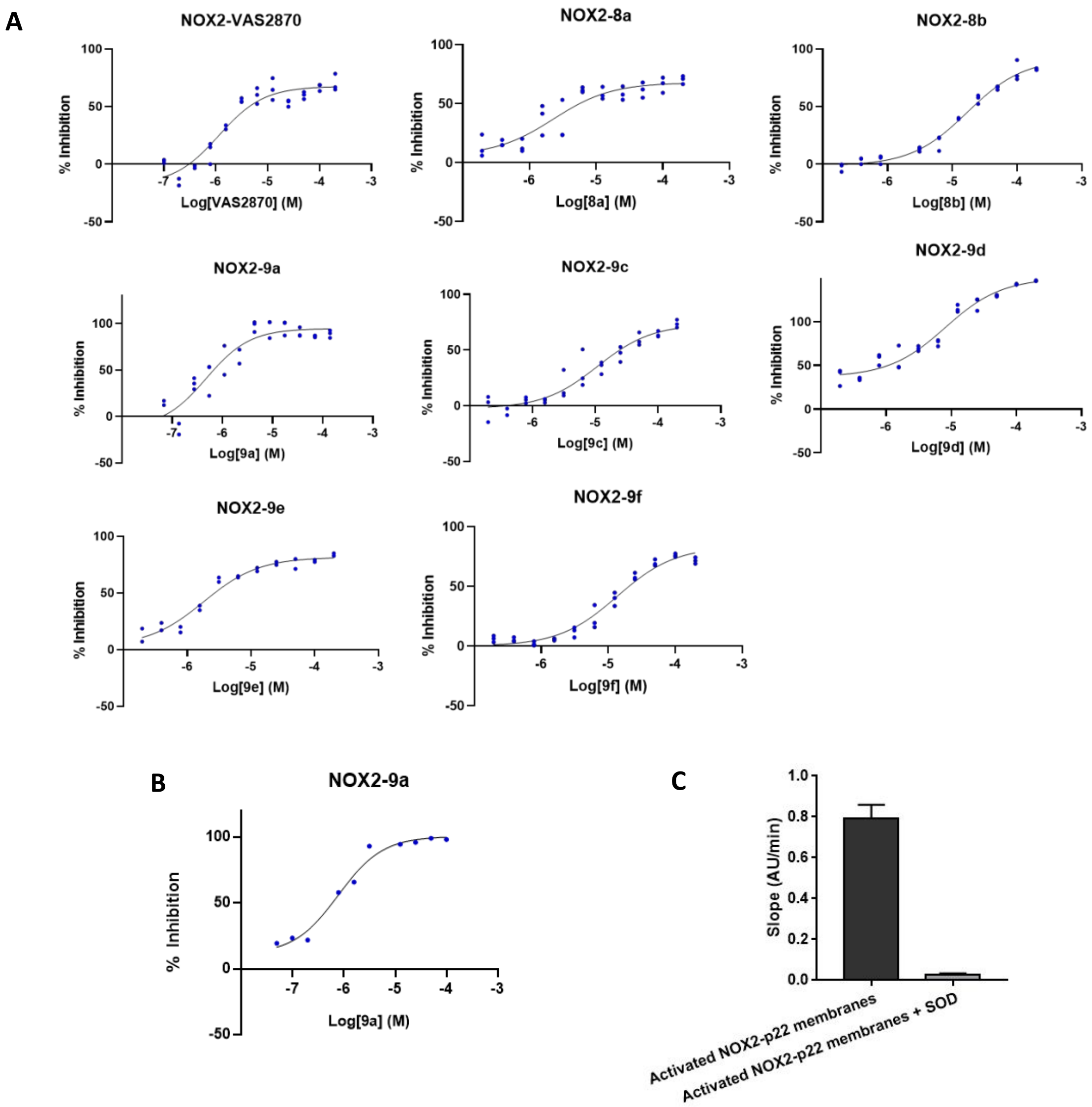

Figure S3. IC_{50} determination for human NOX2 isolated membranes. (**A**) MCLA assay. (**B**) Cytochrome c assay. (**C**) Cytochrome c control assay in the presence of superoxide dismutase (SOD). Progress curves were generated at a constant NADPH concentration (240 µM) in the presence of varying inhibitor concentrations (50 nM to 200 µM), plotting inhibition (%) against the logarithm of inhibitor concentration. IC_{50} values were calculated using nonlinear regression analysis in GraphPad Prism 9.0 (mean ± s.d., n = 3 independent experiments for MCLA assay and cytochrome c reduction control assay with SOD; n = 1 for cytochrome c assay).

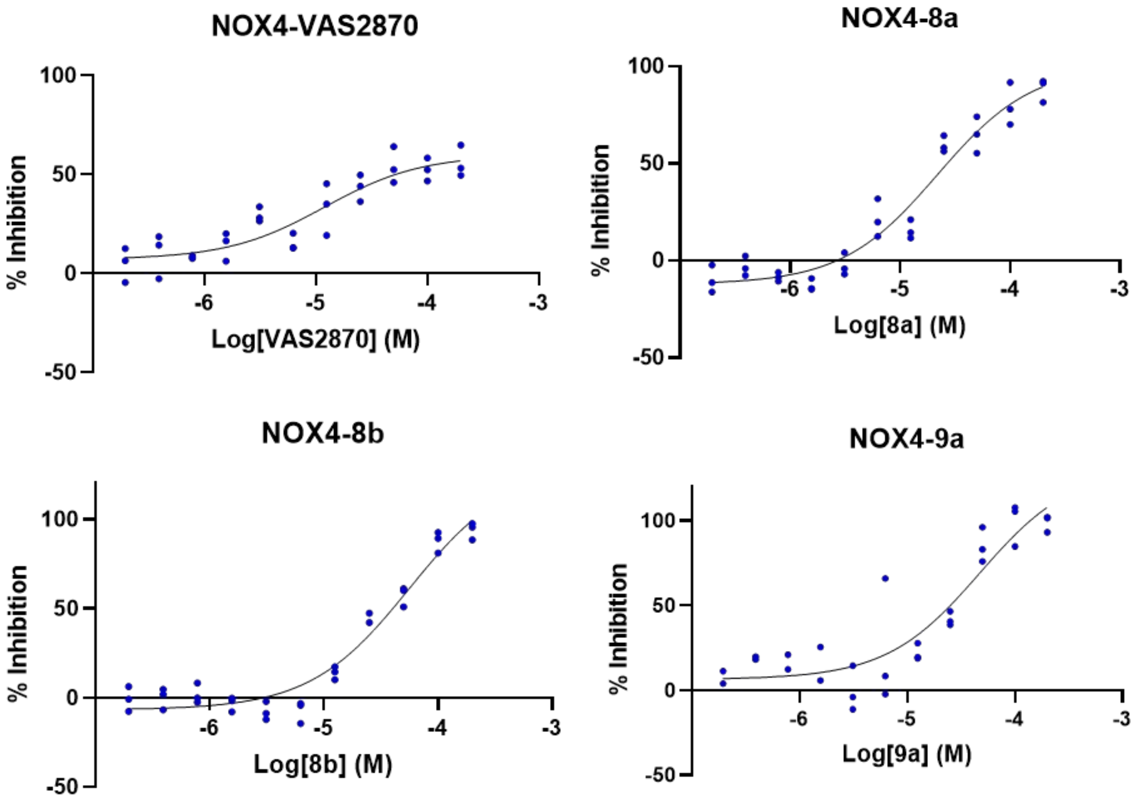

Figure S4. IC_{50} determinations for human NOX4 isolated membranes. Progress curves were generated at a constant NADPH concentration (40 µM) in the presence of varying inhibitor concentrations (70 nM to 200 µM), plotting inhibition (%) against the logarithm of inhibitor concentration. Reactions were followed using the Amplex Red/horseradish peroxidase assay. IC_{50} values were calculated using nonlinear regression analysis in GraphPad Prism 9.0 (mean ± s.d., n = 3 independent experiments).

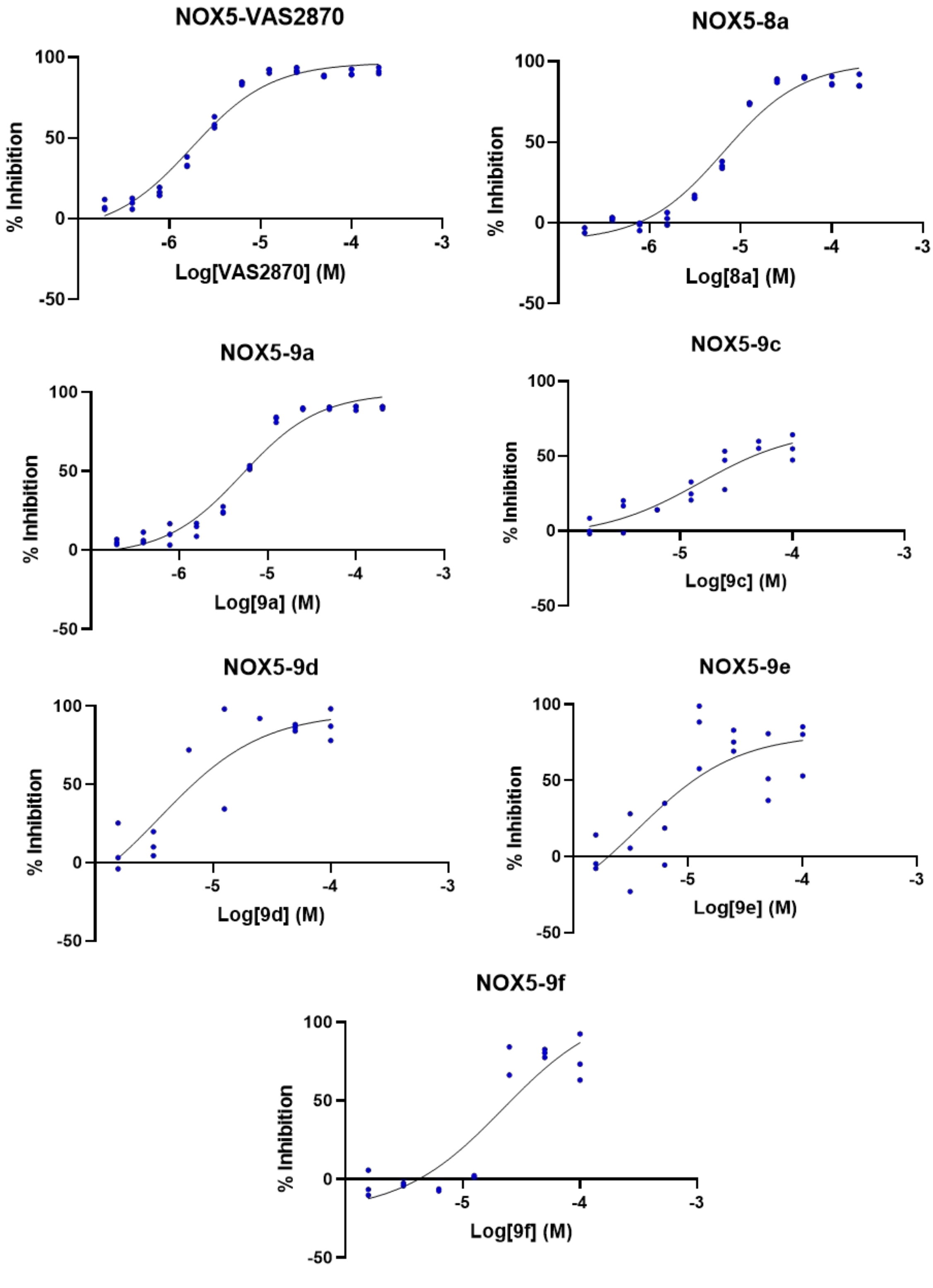

Figure S5. IC_{50} determinations for human NOX5 isolated membranes. Progress curves were generated at a constant NADPH concentration (40 µM) in the presence of varying inhibitor concentrations (70 nM to 200 µM), plotting inhibition (%) against the logarithm of inhibitor concentration. Reactions were followed using the Amplex Red/horseradish peroxidase assay. IC_{50} values were calculated using nonlinear regression analysis in GraphPad Prism 9.0 (mean ± s.d., n = 3 independent experiments).

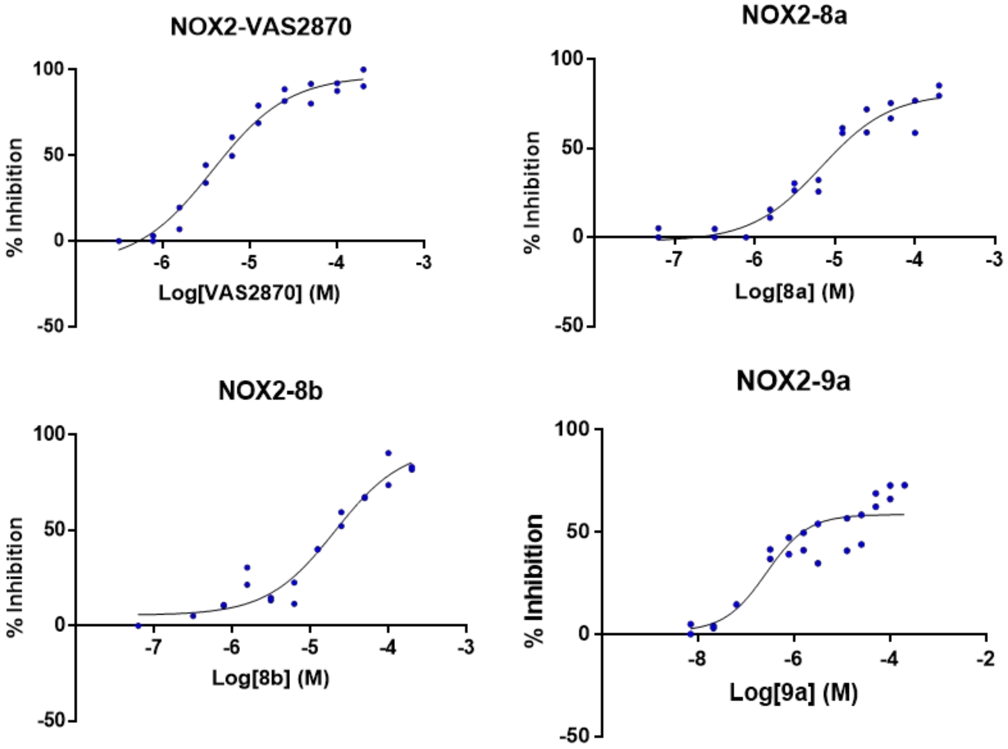

Figure S6. IC_{50} determination on purified human NOX2. Progress curves were generated at a constant NADPH concentration (240 µM) in the presence of varying inhibitor concentrations (7 nM to 200 µM), plotting inhibition (%) against the logarithm of inhibitor concentration. Reactions were followed using the NADPH-consumption assay. IC_{50} values were calculated using nonlinear regression analysis in GraphPad Prism 9.0 (mean ± s.d., n = 2 independent experiments).

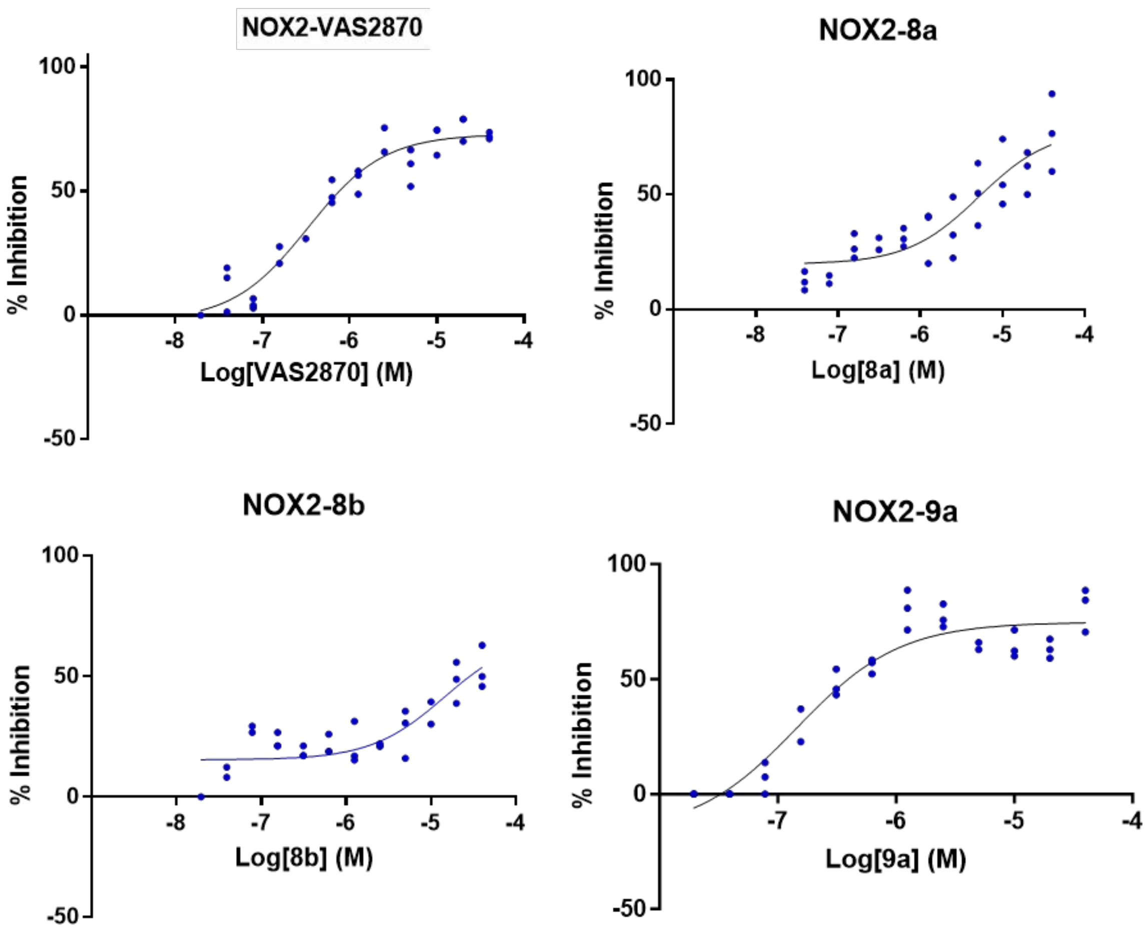

Figure S7. IC_{50} determination on NOX2-overexpressing cells. Progress curves were generated in the presence of varying inhibitor concentrations (40 nM to 40 μM), plotting inhibition (%) against the logarithm of inhibitor concentration. Reactions were followed using the cytochrome c assay. IC_{50} values were calculated using nonlinear regression analysis in GraphPad Prism 9.0 (mean ± s.d., n = 3 independent experiments).

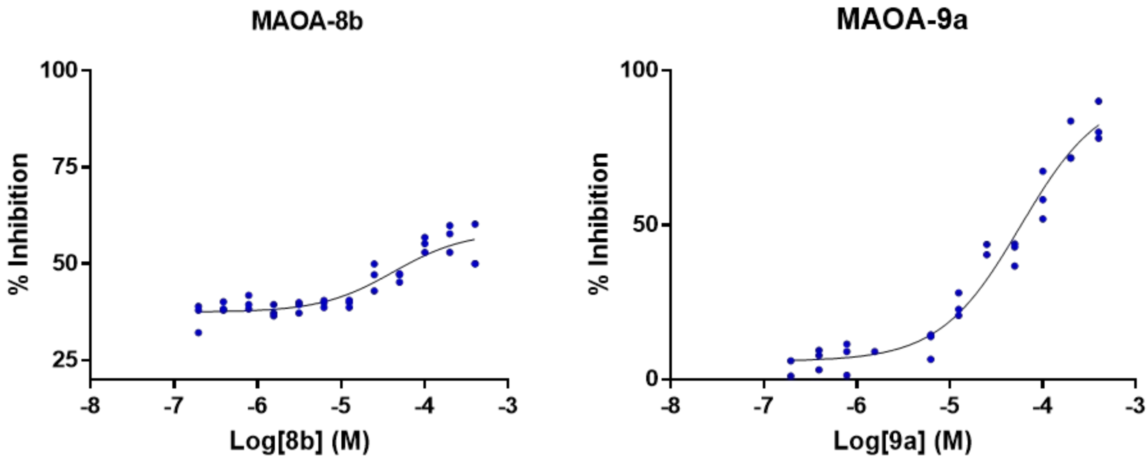

Figure S8. IC_{50} determination on purified human MAOA. Progress curves were generated in the presence of varying inhibitor concentrations (200 nM to 200 μM), plotting inhibition (%) against the logarithm of inhibitor concentration. IC_{50} values were calculated using nonlinear regression analysis in GraphPad Prism 9.0 (mean ± s.d., n = 2 independent experiments).

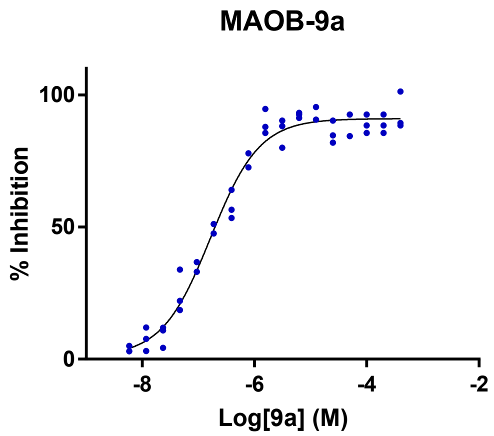

Figure S9. IC_{50} determination on purified human MAOB. Progress curves were generated in the presence of varying inhibitor concentrations (6 nM to 200 µM), plotting inhibition (%) against the logarithm of inhibitor concentration. IC_{50} values were calculated using nonlinear regression analysis in GraphPad Prism 9.0 (mean ± s.d., n = 2 independent experiments).

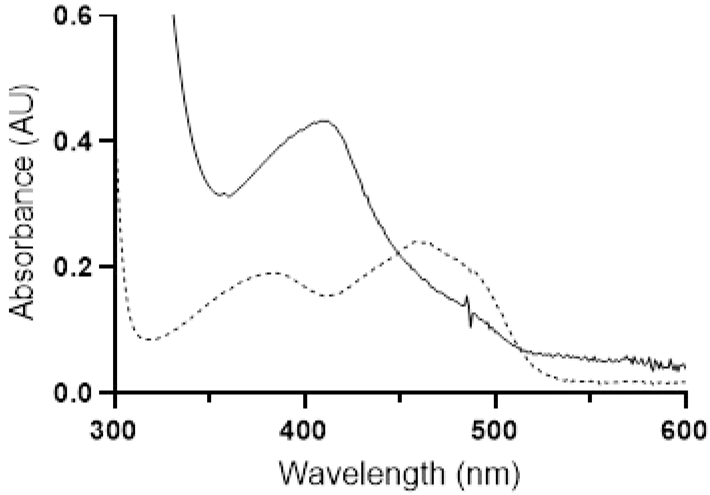

Figure S10. The spectrum of the flavin covalently bound to MAOB is modified upon incubation with 9a. The flavin spectrum recorded before starting the inhibition reaction is shown as a dashed line; the spectrum recorded after 30 min of incubation with **9a** is shown as a continuous line. At the final time point 18.4 µM MAOB cofactor was modified, with a calculated inhibited fraction equal to 91.8 %.

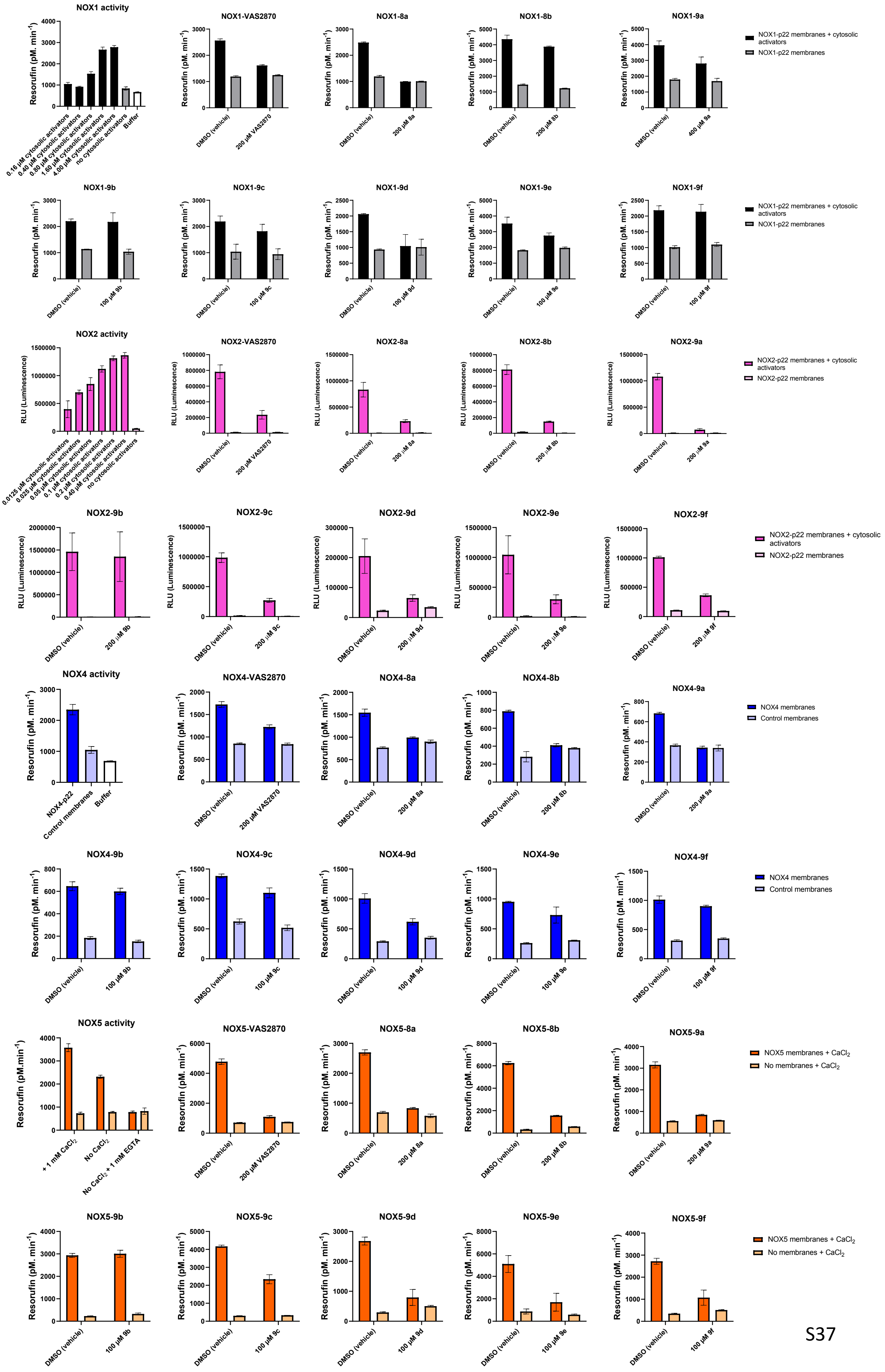
NOX1 activity
Resorufin (pM. min-1)
0.16 µM cytosolic activators
0.40 µM cytosolic activators
0.80 µM cytosolic activators
1.60 µM cytosolic activators
4.00 µM cytosolic activators
no cytosolic activators
Buffer
NOX1-VAS2870
NOX1-8a
NOX1-8b
NOX1-9a
DMSO (vehicle)
200 µM VAS2870
200 µM 8a
200 µM 8b
400 µM 9a
NOX1-p22 membranes + cytosolic activators
NOX1-p22 membranes
NOX1-9b
NOX1-9c
NOX1-9d
NOX1-9e
NOX1-9f
100 µM 9b
100 µM 9c
100 µM 9d
100 µM 9e
100 µM 9f
NOX2 activity
RLU (Luminescence)
0.0125 µM cytosolic activators
0.025 µM cytosolic activators
0.05 µM cytosolic activators
0.1 µM cytosolic activators
0.2 µM cytosolic activators
0.40 µM cytosolic activators
no cytosolic activators
NOX2-VAS2870
NOX2-8a
NOX2-8b
NOX2-9a
200 µM 9a
NOX2-p22 membranes + cytosolic activators
NOX2-p22 membranes
NOX2-9b
NOX2-9c
NOX2-9d
NOX2-9e
NOX2-9f
200 µM 9b
200 µM 9c
200 µM 9d
200 µM 9e
200 µM 9f
NOX4 activity
NOX4-p22
Control membranes
Buffer
NOX4-VAS2870
NOX4-8a
NOX4-8b
NOX4-9a
NOX4 membranes
Control membranes
NOX4-9b
NOX4-9c
NOX4-9d
NOX4-9e
NOX4-9f
NOX5 activity
Resorufin (pM.min-1)
+ 1 mM CaCl2
No CaCl2
No CaCl2 + 1 mM EGTA
NOX5-VAS2870
NOX5-8a
NOX5-8b
NOX5-9a
NOX5 membranes + CaCl2
No membranes + CaCl2
NOX5-9b
NOX5-9c
NOX5-9d
NOX5-9e
NOX5-9f

Figure S11. Hydrogen peroxide or superoxide production by NOXs and their respective controls, measured using the Amplex Red-HRP coupled assay (NOX1, NOX4 and NOX5) or MCLA assay (NOX2). For Amplex Red-HRP coupled assay, slopes (resorufin production expressed in pM min-1) were calculated based on a resorufin calibration performed at the same conditions of the assay. For MCLA assay, values are reported as endpoints (RLU = Relative Luminescence Unit). Data were plotted using GraphPad Prism 9.0 (mean ± s.d., n = 3 independent experiments). Control reactions were performed using non-activated NOX1 (NOX1-p22 membranes), non-activated NOX2 (NOX2-p22 membranes), non-transfected cell membranes (control membranes), and the reaction mixture with no membranes. None of the inhibitors showed any prominent interference since the control reactions are not affected by the addition of the inhibitors.

Table S3. Data Collection and Refinement Statistics for the human MAOB Crystal Structure in Complex with **9a**

	**9a**
Space group	C222
Unit cell axes (Å)	*a* = 130.92<br>*b* = 222.03<br>*c* = 85.88
Resolution (Å)	1.4
PDB code	9FJT
$R_{sym}^{a,b}$ (%)	5.6 (67.1)
$CC_{1/2}^{b}$ (%)	99.8 (62.7)
Completeness^{b} (%)	99.8 (100)
Unique reflections	243,584
Redundancy^{b}	6.4 (6.8)
I/σ^{b}	12.9 (1.7)
N. of non-hydrogen atoms<br>protein/FAD<br>inhibitor<br>detergent^{c}<br>water	9,144<br>7984/2x53<br>23 (ch. A) 32 (ch.B)<br>26<br>973
Average B value for protein/ligands atoms ($Å^2$)	15.2/18.6
$R_{cryst}^{b,d}$ (%)	16.7 (26.6)
$R_{free}^{b,d}$ (%)	18.9 (27.1)
Rms bond length (Å)	0.016
Rms bond angles (°)	1.93

^{a} $R_{sym}=\sum|I_i-\langle I\rangle|/\sum I_i$, where I_i is the intensity of ith observation and <I> is the mean intensity of the reflection.

^{b} Values in parentheses are for reflections in the highest resolution shell.

^{c} As in previous human MAOB structures, one molecule of the Zwittergent 3-12 detergent (used in crystallization experiments) is partly visible in the electron density of each of the two protein monomers present in the asymmetric unit

^{d} $R_{cryst}=\sum|F_{obs}-F_{calc}|/\sum|F_{obs}|$ where F_{obs} and F_{calc} are the observed and calculated structure factor amplitudes, respectively. R_{cryst} and R_{free} were calculated using the working and test sets, respectively.

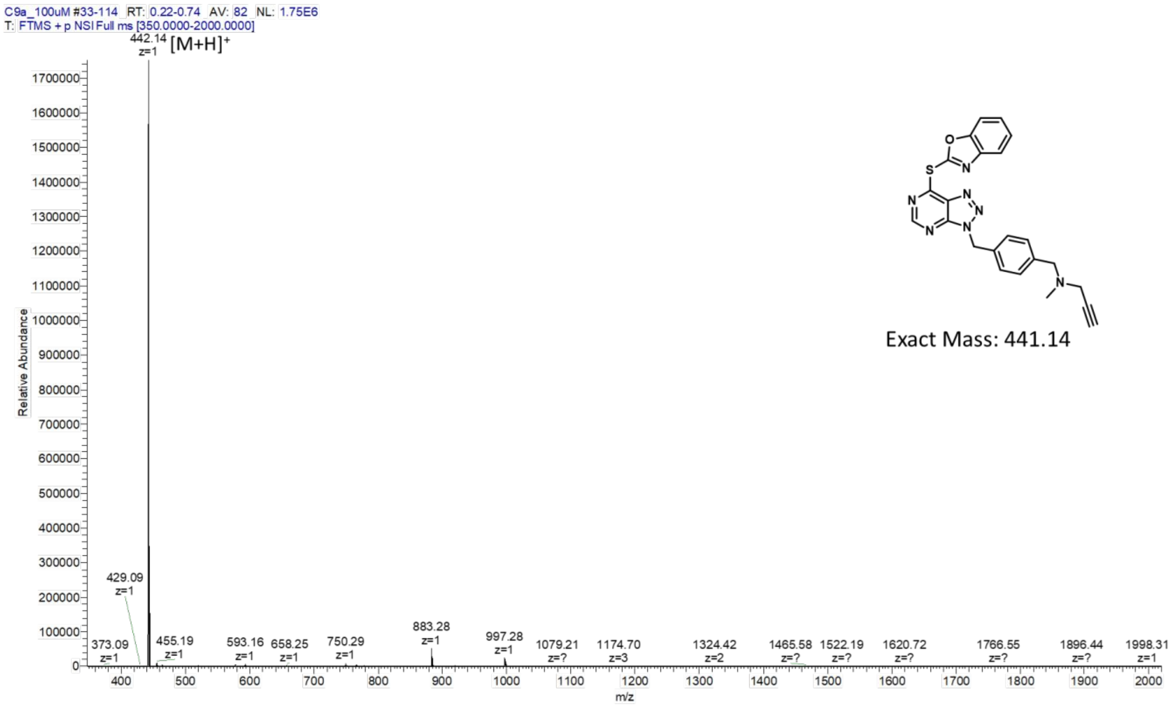

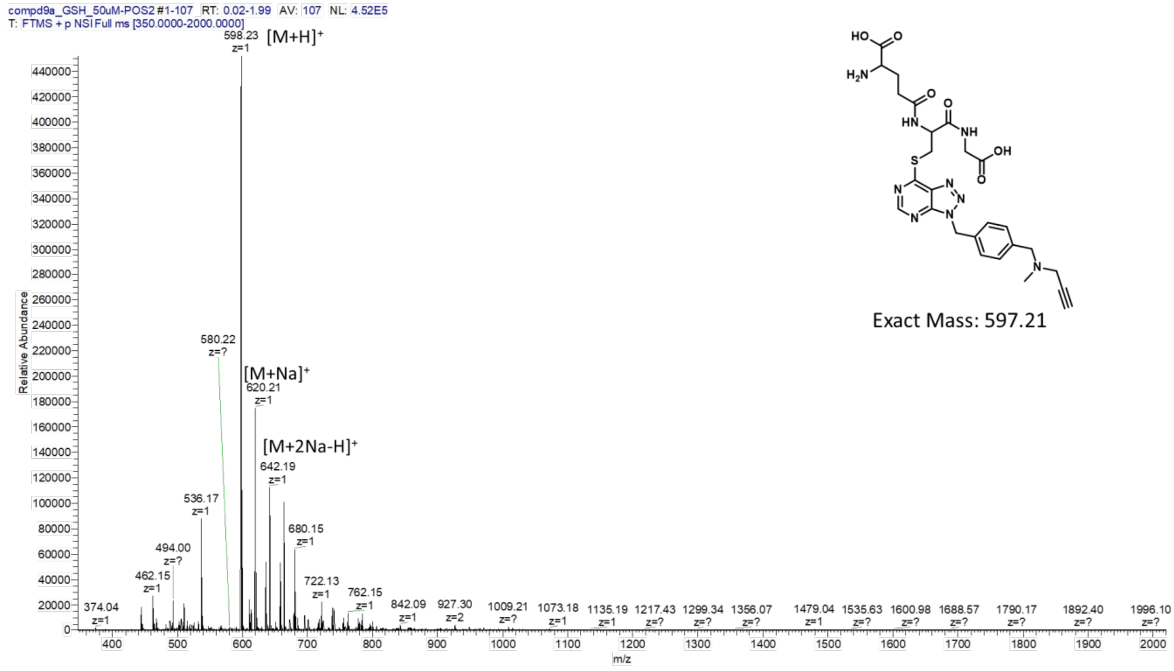

Figure S12. Mass Spectra of 9a and 9a + GSH. The reaction was performed between GSH (10 μM) and 9a (10 μM) (1:1) in ammonium acetate solution (200 mM, pH 7.4). GSH was previously solubilized in H_2O (suitable for LC-MS analysis) while 9a was first solubilised in DMSO (stock solution of 10 mM). Compound **9a** readily reacted with GSH to form the corresponding adduct, consistent with previous reports on structurally similar compounds, such as VAS2870.

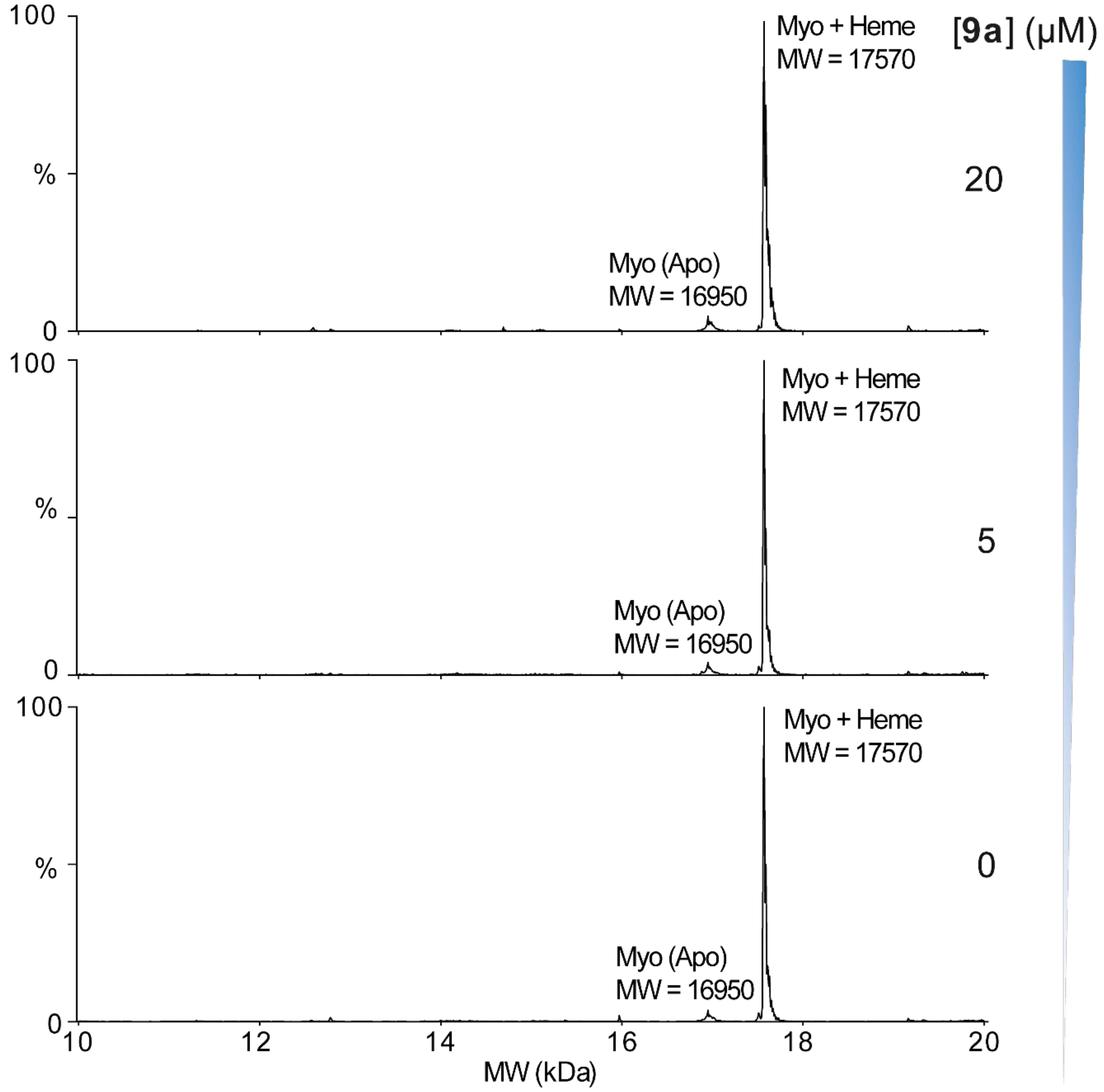

Figure S13. Deconvoluted mass spectra of myoglobin (1 µM) incubated with 9a at concentrations of 5 µM and 20 µM in ammonium acetate solution (200 mM, pH 7.5) for 30 minutes. The reactions were analyzed using native mass spectrometry. In these conditions, no adduct formation was detected between **9a** and myoglobin. Myoglobin (human cardiac, Sigma Aldrich, M6036) was used for this study.

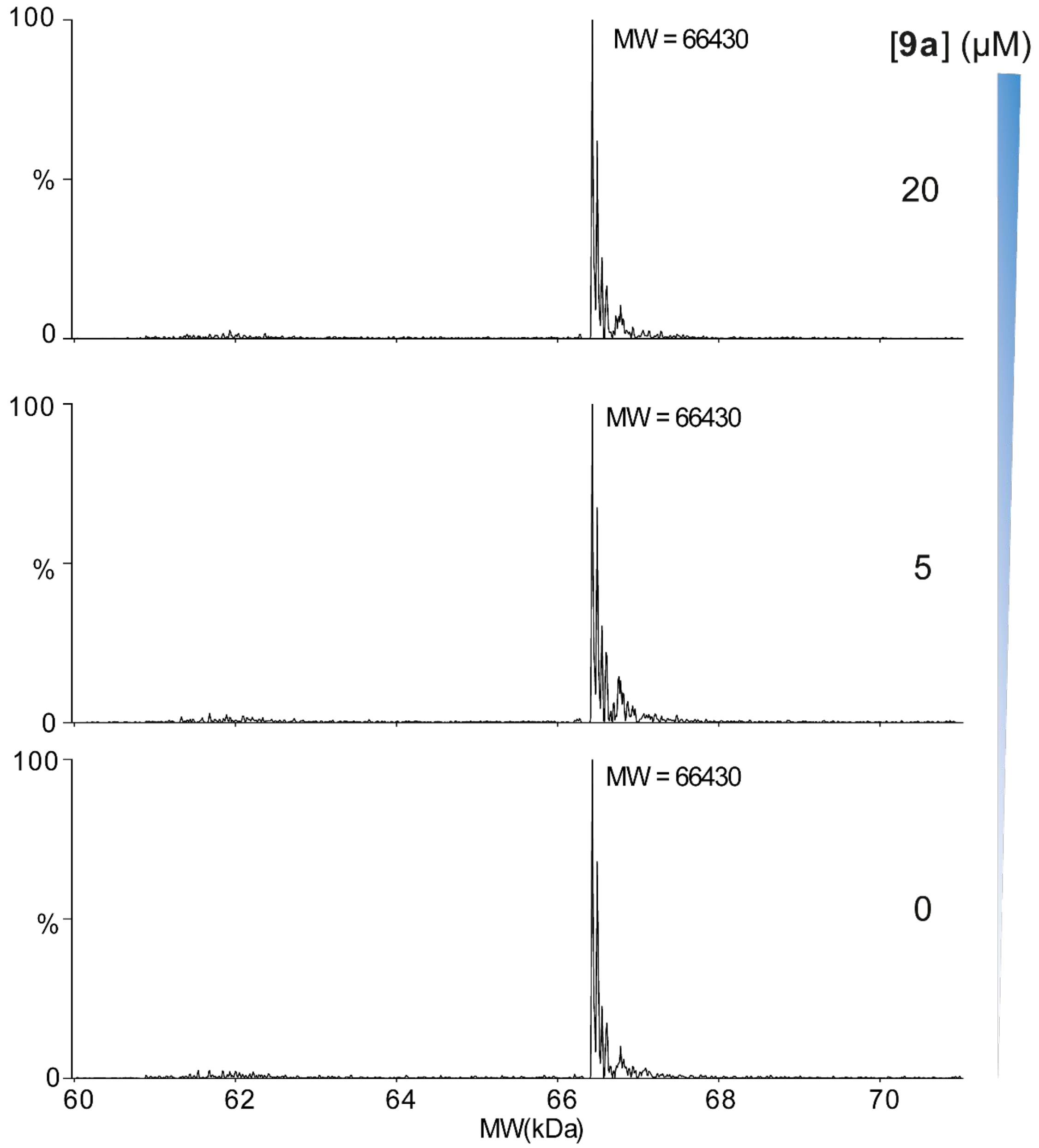

Figure S14. Deconvoluted mass spectra of Bovine Serum Albumin (BSA, 1 µM) incubated with 9a at concentrations of 5 µM and 20 µM in ammonium acetate solution (200 mM, pH 7.5) for 30 minutes. The reactions were analyzed using native mass spectrometry. In these conditions, no adduct formation was detected between **9a** and BSA. Bovine serum albumin (BSA, Sigma Aldrich, A7030) was used for this study.

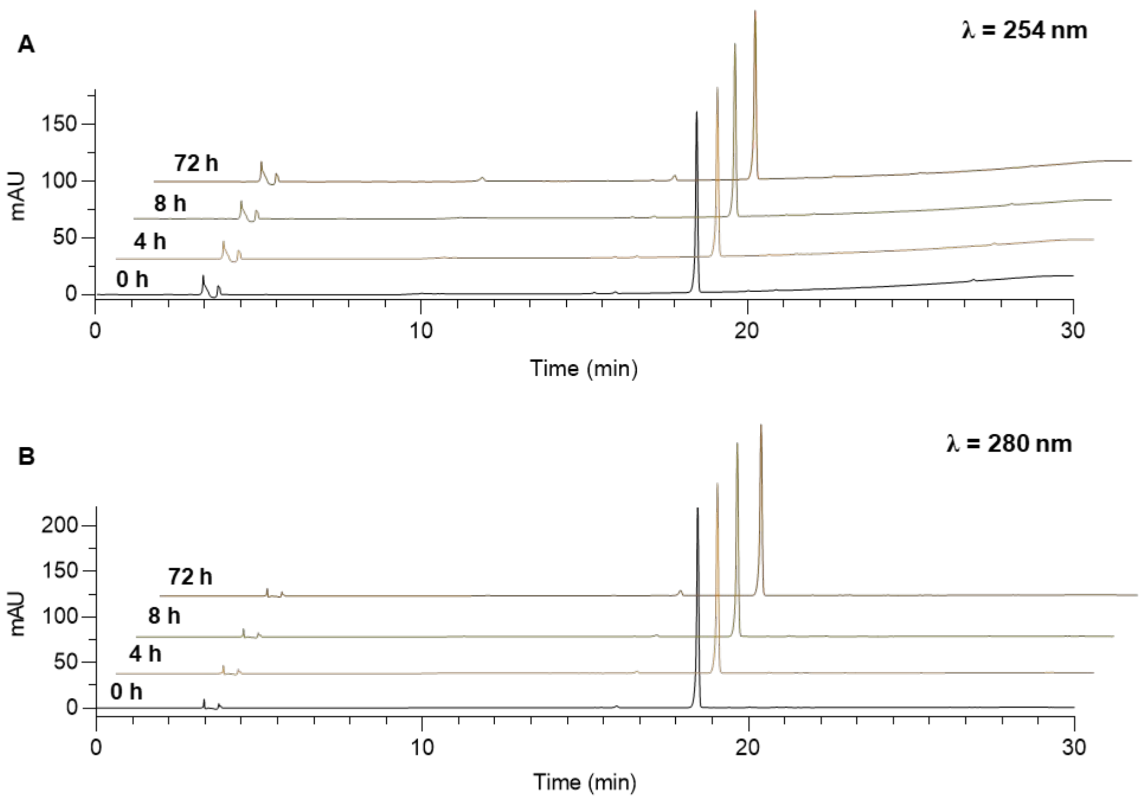

Figure S15. HPLC traces of compound 9a incubated in H_2O for 0, 4, 8, and 72h. Compound 9a was diluted in H_2O (final concentration = 0.1 mM) from a 10 mM stock in DMSO. The solution was incubated at rt and then analyzed through analytical HPLC under the same conditions described in the Experimental Section. HPLC runs were performed after 0, 4, 8, and 72 h of incubation and acquired at two wavelengths: 254 nm (A), and 280 nm (B).

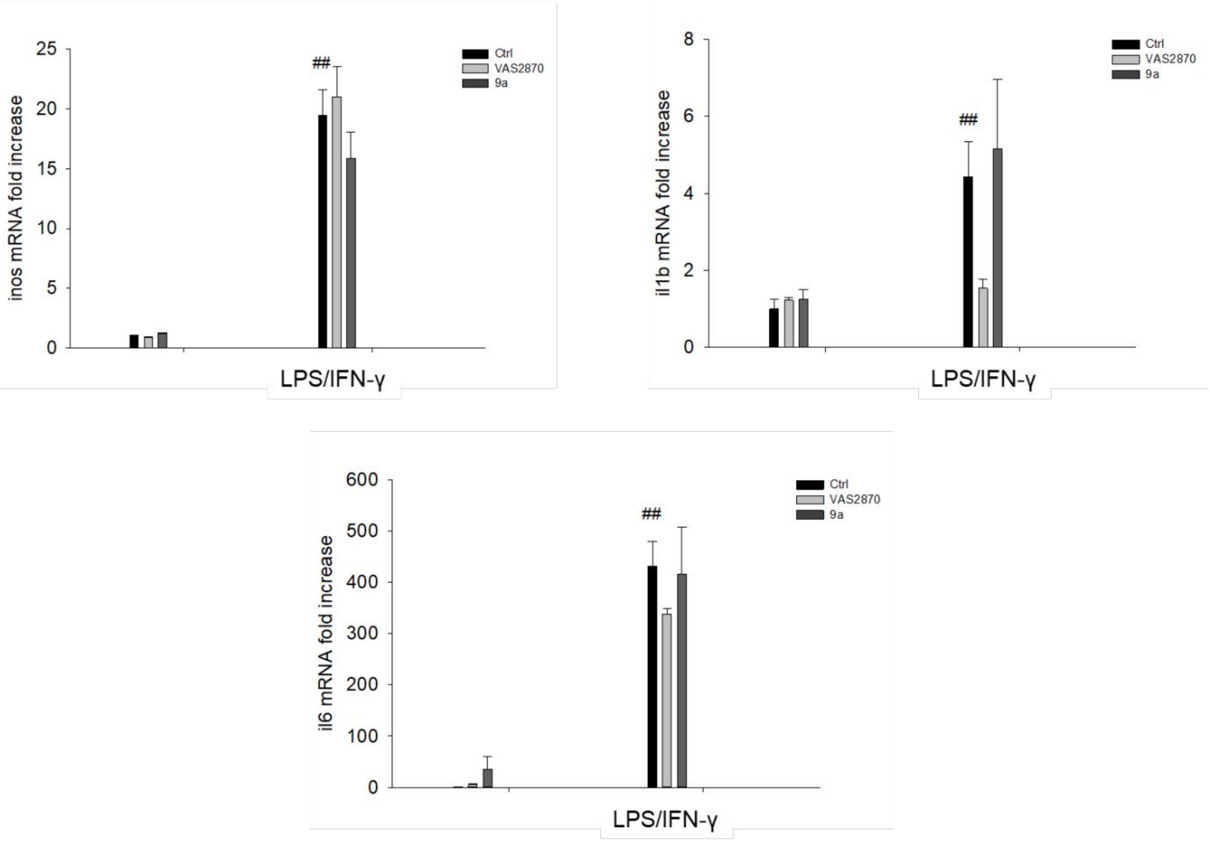

Figure S16. qRT-PCR analysis for the indicated transcripts in BV2 cells, pre-stimulated with LPS/IFN-γ for 24h and then treated with the compounds at the concentration of 10 µM for 24 h. CTR (control) represent the cells treated with the vehicle (DMSO). The values are expressed as fold of expression versus the control (arbitrary value = 1) and shown as mean ± SD. Statistically significant differences are reported ($^{\#}$, $p<0.05$; $^{\#\#}$, $p<0.01$) for three independent experiments.
